# Methylation across the central dogma in health and diseases: new therapeutic strategies

**DOI:** 10.1038/s41392-023-01528-y

**Published:** 2023-08-25

**Authors:** Ruochen Liu, Erhu Zhao, Huijuan Yu, Chaoyu Yuan, Muhammad Nadeem Abbas, Hongjuan Cui

**Affiliations:** 1 State Key Laboratory of Resource Insects, Medical Research Institute, Southwest University, Chongqing, 400715 China; 2 Jinfeng Laboratory, Chongqing, 401329 China; 3 Chongqing Engineering and Technology Research Center for Silk Biomaterials and Regenerative Medicine, Chongqing, 400716 China; 4 Engineering Research Center for Cancer Biomedical and Translational Medicine, Southwest University, Chongqing, 400715 China

**Keywords:** Molecular medicine, Epigenetics

## Abstract

The proper transfer of genetic information from DNA to RNA to protein is essential for cell-fate control, development, and health. Methylation of DNA, RNAs, histones, and non-histone proteins is a reversible post-synthesis modification that finetunes gene expression and function in diverse physiological processes. Aberrant methylation caused by genetic mutations or environmental stimuli promotes various diseases and accelerates aging, necessitating the development of therapies to correct the disease-driver methylation imbalance. In this Review, we summarize the operating system of methylation across the central dogma, which includes writers, erasers, readers, and reader-independent outputs. We then discuss how dysregulation of the system contributes to neurological disorders, cancer, and aging. Current small-molecule compounds that target the modifiers show modest success in certain cancers. The methylome-wide action and lack of specificity lead to undesirable biological effects and cytotoxicity, limiting their therapeutic application, especially for diseases with a monogenic cause or different directions of methylation changes. Emerging tools capable of site-specific methylation manipulation hold great promise to solve this dilemma. With the refinement of delivery vehicles, these new tools are well positioned to advance the basic research and clinical translation of the methylation field.

## Introduction

The three protagonists of the central dogma of molecular biology, DNA, RNA, and protein, are subjected to various post-synthesis chemical modifications. The flow of genetic information from DNA to RNA to protein and consequent protein load and function are strictly regulated by post-synthesis modifications, which gives rise to phenotypic variations in cells/organisms with the same/similar genetic origins. One of the most prevalent modifications is methylation which uses S-adenosylmethionine (SAM) as the donor of a methyl group and replaces a hydrogen atom. As a result, the physicochemical properties of the methylated substrates are altered, including stability and affinity for binding partners or methyl-binding proteins (“readers”). The existence of demethylases (“erasers”) that remove methylation installed by methyltransferases (“writers”) at DNA, RNAs, and proteins indicates the dynamic property of the different methylation pathways. This dynamic nature is consistent with its key regulatory roles in health, and the disturbance of the dynamics is associated with various diseases and constitutes a rationale for therapeutic remedies.^1^

In mammalian genomes, DNA methylation has been identified at the carbon-5 position of cytosine and recently at the nitrogen-6 position of adenosine, generating *C*^5^-methylcytosine (5mC) and *N*^6^-methyladenosine (6 mA), respectively. 5mC is the predominant DNA modification and occurs almost exclusively at the symmetric CpG dinucleotides in most somatic cells or tissues; specifically, 60–80% of the 32.28 million CpG dinucleotides in the human genome are methylated.^2,3^ The majority of remaining unmethylated CpG dinucleotides are located near the transcription start sites in dense clusters known as CpG islands. Besides, non-CpG methylation, namely CpH methylation (where H = A, T or C), is prevalent in human embryonic stem cells and brain.^2,4^ Recently, A DNA cytosine methylation atlas of normal human cell types has been determined by deep whole-genome bisulfite sequencing, providing a key resource for the investigation of gene regulation and disease-associated variation, and abundant tissue-specific biomarkers for liquid biopsies.^5^ The effect of DNA cytosine methylation is context-dependent; for example, its presence on gene regulatory sequences (promoters or enhancers) usually causes transcriptional silence, whereas it is not associated with repression and may promote transcription when present on gene bodies.^6^ DNA adenine methylation, in contrast to DNA cytosine methylation, is a relatively new type of epigenetic modification. Although its existence, genomic distribution pattern, and biological functions in more recently evolved eukaryotes are still being debated mainly due to the low abundance of 6 mA, multiple studies have reported that 6 mA is implicated in regulating transcription, transposon activity, disease, and other functions.^7–9^

The six billion bases of the human genome are wrapped around ~30 million histone octamers (H2A, H2B, H3, and H4) termed chromatin. Histone methylation, primarily on the side chains of lysine (Lys) and arginine (Arg) residues, either upregulates or downregulates transcription depending on the location within histone proteins and the degree of methylation. For example, histone Lys residues can be mono-, di- or tri-methylated; mono-methylation at H3K27 and di- and tri-methylation at H3K4, H3K36, and H3K79 are generally associated with gene activation, while tri-methylation at H3K27 and H3K9 with gene repression.^10,11^ Histone Arg residues can be mono-, symmetrically, or asymmetrically di-methylated (me1, me2s, or me2as, respectively); H3R2me2s, H3R17me2as, and H4R3me2as generally act as activation marks, while H3R2me2as, H3R8me2s, and H4R3me2s are repressive marks.^12–15^ There is extensive crosstalk between histone methylation and DNA methylation. Together with histone acetylation which is often interdependent or mutually exclusive with certain types of histone methylation and DNA methylation, they form the fundamental mechanism of epigenetic regulation that assures the somatic inheritance of gene expression patterns.

Beyond epigenetic regulation, methylation of RNAs and non-histone proteins provides two additional layers for governing gene expression and function. All types of RNAs including messenger RNA (mRNA), ribosomal RNA (rRNA), transfer RNA (tRNA), micro RNA (miRNA), and long non-coding RNA (lncRNA) are substrates for methylation reaction. More than 70 types of RNA methylation have been identified, such as *N*^7^-methylguanosine (m^7^G), *N*^6^-methyladenosine (m^6^A), *C*^5^-methylcytosine (m^5^C; not to be confused with DNA *N*^6^-methyladenosine (6 mA) and *C*^5^-methylcytosine (5mC)), and 2′-*O*-methyl (N_m_) (where N = A, U, G, or C). Broad interest in RNA methylation biology has been re-inspired by the discovery of the significant level and function of mRNA internal modifications, primarily m^6^A which is the most abundant one and regulates splicing, localization, translation, and stability of mRNAs. Nonhistone protein methylation also mainly occurs at Lys and Arg residues and shares the common set of catalytic enzymes with histone methylation to regulate the activity, stability, and subcellular localization of methylated proteins. As histones are just a subset of the thousands of proteins targeted for methylation, the interpretation of the mechanisms of protein methylation writers, erasers, and readers in health and diseases is challenging. Moreover, there is extensive crosstalk among protein, RNA, and DNA methylation in various biological processes, generating a sophisticated regulatory network. In this Review, we summarize the operating system of methylation across the central dogma, which involves writers, erasers, readers, and reader-independent outputs. We then discuss how dysregulation of the system contributes to neurological disorders, cancer, and aging, and the present and emerging therapeutic strategies.

## Mechanism and function of DNA/RNA/protein methylation

### Writers and erasers of methylation

**Fig. 1:**
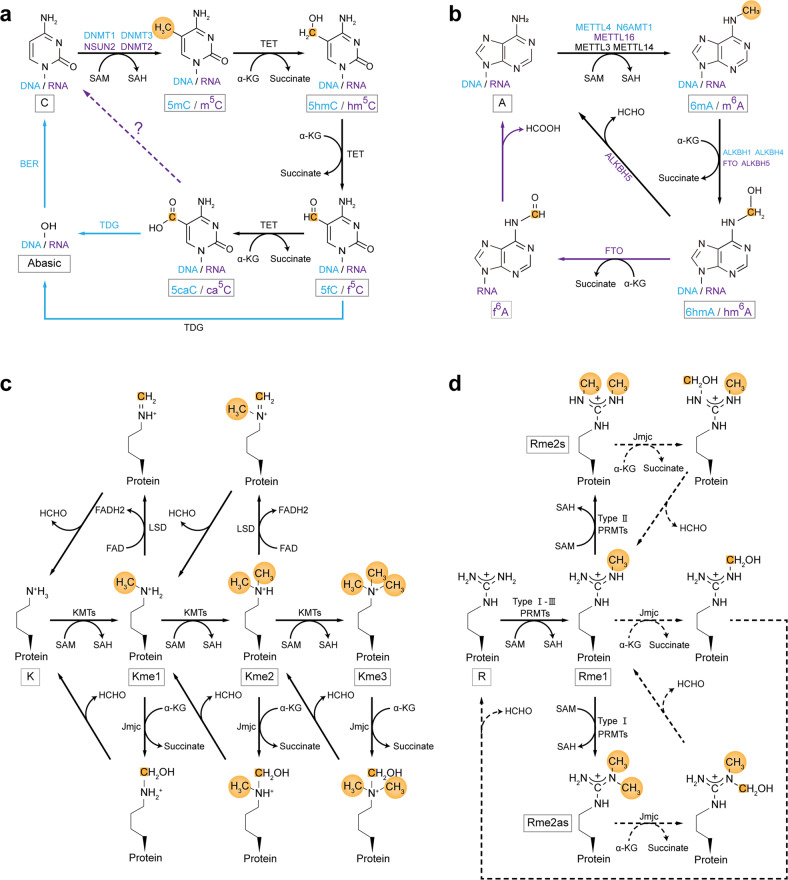
Biochemical processes of reversible DNA/RNA/protein methylation. **a** *C*^5^-cytosine methylation and demethylation in DNA and RNAs. Blue fonts and arrows represent components of the DNA methylation pathway, purple fonts and arrows represent components of the RNA methylation pathway, black fonts and arrows represent common components, and dashed arrows indicate potential steps. Methyl groups and carbon atoms are highlighted in gold. **b** *N*^6^-adenosine methylation and demethylation in DNA and RNAs. The rule of color usage is the same as that of in (**a**). **c** Protein lysine methylation and demethylation. **d** Protein arginine methylation and demethylation. Dashed arrows indicate potential steps

The effectors in DNA, RNA, and protein methylation pathways are categorized into three groups: writers, erasers, and readers, which add, remove, and recognize methyl signals, respectively (Fig. 1). There are ~200 genes in the human genome encoding known or putative SAM-dependent methyltransferases, which have been grouped according to distinct conserved structures. The seven-β-strand domain (7βS) superfamily is the largest group with roughly 130 members, containing DNA methyltransferase (DNMT), Nol1/Nop2/Sun (NSUN), MT-A70, and protein Arg methyltransferase (PRMT) subfamilies, and catalyzes a wide range of substrates including nucleic acids, proteins, and metabolites. For instance, *C*^5^-cytosine methylation in DNA is catalyzed by three active writers of the DNMT family: DNMT3A, DNMT3B, and DNMT1. DNMT3A and DNMT3B are mainly responsible for de novo DNA methylation and DNMT1 for the maintenance of the established DNA methylation pattern during cell division. Two other members of the DNMT family, DNMT2 (also known as tRNA aspartic acid methyltransferase 1) and DNMT3L, are not catalytically active DNA methyltransferases. DNMT2 functions as a tRNA methyltransferase, and DNMT3L acts as a de novo DNA methyltransferase cofactor that stimulates their activity specifically in the germline.^16^ *C*^5^-cytosine methylation in mRNA is primarily catalyzed by NSUN2 and NSUN6 of the NSUN family which contains a conserved SUN domain with enzymatic activity. NSUN2 catalyzes the m^5^C sites that locate at the 5′ ends of hairpin structures and have a 3′ G-rich triplet motif, while NSUN6 acts on the m^5^C sites that locate at the loops of hairpin structures and have a 3′ UCCA motif.^17–21^ Another writer that installs m^5^C in mRNA is DNMT2, especially at the DNA damage sites.^22,23^ For DNA *N*^6^-adenosine methylation, three putative *N*^6^-adenosine methyltransferases have been reported, i.e., methyltransferase-like 4 (METTL4), METTL3- METTL14 complex of MT-A70 family, and N-6 adenine-specific DNA Methyltransferase 1 (N6AMT1) (also known as KMT9).^24–26^ Bewilderingly, METTL4 can catalyze *N*^6^-methylation of 2′-*O*-methyladenosine (Am) to generate *N*^6^,2′-*O*-dimethyladenosine (m^6^Am) in U2 small nuclear RNA (snRNA); METTL3- METTL14 complex is well established as RNA m^6^A writer; N6AMT1 has been reported to be involved in protein methylation at glutamine (Gln) and Lys residues; thus further study is needed to confirm the specificity and physiological relevance of these putative *N*^6^-adenosine methyltransferases.^7,9^ The majority of m^6^A in mRNAs are catalyzed by METTL3- METTL14 complex that prefers the sequence motif RRACH (R = A or G; H = A, C, or U), of which METTL3 is the catalytic subunit and METTL14 is an allosteric adaptor.^27^ Additional adaptors for this writer complex are Wilms’ tumor 1-associated protein (WTAP), Vir like m^6^A methyltransferase associated protein, zinc finger CCCH domain-containing protein 13 (ZC3H13), RNA binding motif protein 15/15B (RBM15/15B), and HAKAI (also known as CBLL1). The remaining small number of m^6^A in mRNAs are catalyzed by METTL16 which prefers the UAC(m6A)GAGAA sequence presented as a loop in a hairpin structure. Histone and nonhistone protein methylation at Arg residue is performed by the PRMT family, of which nine members have been identified in the human genome. They are categorized into three types: PRMT1,2,3,4 (also known as CARM1),6, and 8 are type I enzymes that perform mono- and asymmetric di-methylation; PRMT5 and 9 are type II enzymes that mediate mono- and symmetric di-methylation; PRMT7, the only type III PRMT, only be able to catalyze mono-methylation of Arg. Most PRMTs methylate Gly–Arg-rich motifs within their nonhistone substrates, while PRMT4 methylates Pro-Gly-Met-rich motifs and PRMT5 can di-methylate both motifs. Protein Lys methylation is primarily catalyzed by the SET (Su(var)3–9, Enhancer-of-zeste, Trithorax) domain family which is the second largest group with roughly 50 members. Based on sequence similarities surrounding the SET domain, these Lys methyltransferases (KMTs) are classified into seven main subfamilies, i.e., SUV39, SET1, SET2, EZ, SMYD, SUV4-20, and RIZ (PRDM).^28^ Several additional 7βS superfamily KMTs with no SET domain have been identified, including DOT1L,^29^ METTL13,^30^ VCPKMT,^31^ and the above-mentioned N6AMT1.^32^ KMTs exhibit high specificity with regard to the location within histone proteins and the degree of methylation. For example, SUV39H1 of SUV39 family, the first identified human KMT, catalyzes trimethylation of H3K9 (H3K9me3);^33,34^ mixed-lineage leukemia 3 (MLL3) and MLL4 of SET1 family catalyze monomethylation and dimethylation of H3K4 (H3K4me1 and H3K4me2);^35^ DOTL1 can mono-, di- or tri-methylate H3K79 in a non-processive manner to generate H3K79me1/2/3.^36,37^

The methyl groups on DNA, RNAs, and proteins can be removed by Fe(II) and α-ketoglutarate (α-KG) dependent dioxygenase superfamily, including ten-eleven translocation (TET), AlkB, and Jumonji C (JmjC) subfamilies. DNA 5mC demethylation is mediated by TET family members (TET1, 2, and 3), in which 5mC is iteratively oxidized to 5-hydroxymethylcytosine (5hmC), 5-formylcytosine (5fC), and 5-carboxylcytosine (5caC). All these oxidized derivatives are unable to be recognized by DNMT1, are passively lost during DNA replication, and are replaced with unmethylated cytosine. Alternatively, 5fC and 5caC can be actively reverted to unmethylated cytosine in a DNA replication-independent manner by thymine DNA glycosylase (TDG)-mediated base excision repair. Interestingly, TET enzymes can also mediate the stepwise oxidation of m^5^C in mRNA, resulting in hm^5^C, f^5^C, and ca^5^C.^38–40^ Whether and how f^5^C and ca^5^C, like their DNA counterparts, contribute to methylation reversibility remains unknown. Although decarboxylation of ca^5^C in mRNA provides a possible pathway to restore unmethylated cytosine,^41^ evidence of these steps still lacks. Nine AlkB members are identified in the human genome, including ALKBH1-8 and FTO (fat mass and obesity-associated protein). It has been proposed ALKBH1 and 4 demethylate 6 mA of DNA, while ALKBH5 and FTO demethylate m^6^A of mRNAs. Oxidization of DNA 6 mA by ALKBH1 or 4 generates an unstable intermediate 6-hydroxymethyladenine (6hmA) that undergoes spontaneous loss of the methyl group as formaldehyde and regenerates an unmethylated adenine. Similarly, FTO can successively oxidize RNA m^6^A to *N*^6^-hydroxymethyladenosine(hm^6^A) and *N*^6^-formyladenosine (f^6^A) which undergo spontaneous hydrolyzation to generate unmethylated adenine and formaldehyde (from hm^6^A) or formic acid (from f^6^A). Unlike FTO, ALKBH1, and 4, ALKBH5 can efficiently catalyze the fragmentation of the hemiaminal intermediate to generate formaldehyde and unmethylated adenine directly.^42^ JmjC family with more than 30 members constitutes the largest class of demethylases, and roughly 20 members of the family have been assigned as lysine demethylases (KDMs) that can demethylate mono-, di-, and tri-methylated Lys using a strategy similar to the demethylation of *N*^6^-methyladenosine by AlkB family.^43^ Based on the domain architecture of the full-length proteins, the family members are classified into seven groups: JHDM1, JHDM2, PHF2/PHF8, JARID1/JARID2, JHDM3/JMJD2, UTX/UTY, and JmjC-domain-only groups. Like KMTs, KDMs exhibit specificity with regard to the site within histone proteins and the degree of methylation. For example, JHDM1A of the JHDM1 group, the first identified JmjC domain-containing demethylase, specifically demethylates H3K36me1/2.^44^ Besides Fe(II) and α-KG-dependent dioxygenases, two members of the superfamily of the flavin adenine dinucleotide (FAD)-dependent amine oxidases were characterized as KDMs, lysine-specific demethylase 1 (LSD1, also known as KDM1A) and LSD2 (also known as KDM1B). They oxidize the methylamine to generate a labile intermediate, imine, which is hydrolyzed to give formaldehyde and demethylated substrate by a non-enzymatic process. The LSD enzymes only demethylate mono- and di-methylated Lys residues, not tri-methylated ones, due to the limitations of the imine-forming catalytic mechanism.^45^ LSD1, the first histone demethylase identified, can catalyze the demethylation of H3K4me1/2, H3K9me1/2, and nonhistone substrates (e.g., DNMT1 and p53), while LSD2 has only been shown activity on H3K4me1/2.^41,46^ Although a dedicated methylarginine demethylases (RDMs) is yet to be identified, some members of the JmjC family have been proposed as RDM candidates, including JMJD6, JMJD1B, and JMJD2A.^47–49^ JMJD6 was the first reported RDM that specifically demethylates H3R2me2 and H4R3me1/2,^48^ however, multiple studies showed that JMJD6 functions as a lysyl hydroxylase rather than a RDM.^50–52^ JMJD1B, a KDM of the JmjC family for H3K9me2 demethylation, also catalyzes the demethylation of H4R3me2s and H4R3me1.^47^ Additional multiple KDMs of the JmjC family, including JMJD2A, have been reported to possess RDM activity in vitro, but their RDM activities and functions in vivo have not been reported.^49^ The biochemical processes of writing and erasing methyl signals at DNA/RNAs/proteins are summarized in Fig. 1.

### Functional interpretation of methylation: readers and beyond

#### Readers

**Fig. 2:**
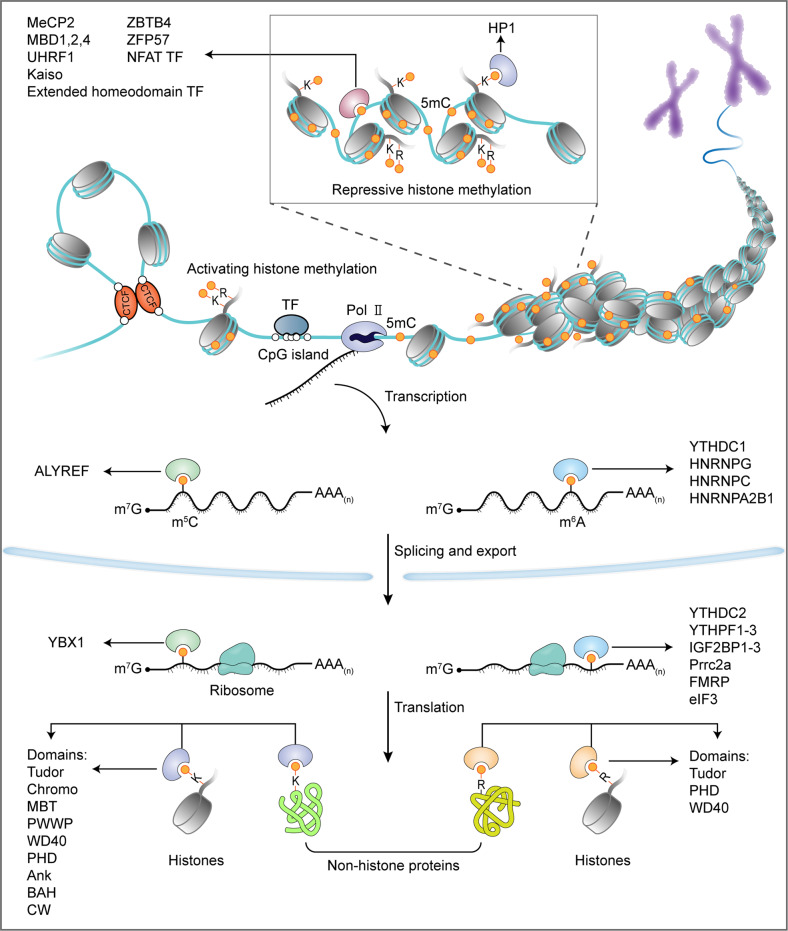
Methylation of DNA/RNAs/proteins regulates the flow of genetic information. In the central dogma of molecular biology, genetic information is transmitted from DNA to RNA to protein. DNA and histone methylation has essential roles in regulating chromatin opening (involving activating histone methylation, e.g., H3K4me3, H3K36me3, and H3R2me2s) for gene transcription or compaction (involving DNA cytosine methylation and repressive histone methylation, e.g., H3K9me3, H3K27me3, and H3R8me2s) for gene silencing. The methylation of mRNAs (m^6^A and m^5^C) regulates the splicing, localization, translation, and stability of the mRNAs. Nonhistone protein methylation influences the activity, stability, and subcellular localization of translated proteins. Collectively, methylation of the different macromolecules constitutes a multilayer dynamic regulation of biological processes. A large array of readers that contain conserved methyl-group binding domains are involved in interpreting these post-synthesis chemical modifications

The functional consequences of DNA/RNA/protein methylation depend on the site and/or the degree of methylation. Methylation substantially alters the hydration, hydrophobicity, and hydrogen-bonding capacity of the methylated residues, which in turn directly or indirectly influence the local structure, interacting proteins, stability, localization, and activity of the methylated macromolecules. The most widely studied mechanism of functional interpretation of methylation is the recruitment of effector proteins (also termed “readers”) at the methylated sites, which triggers downstream cellular processes (Fig. 2). Canonical direct and robust methyl signal readers are those that contain conserved methyl-group binding domains.

Three types of domains can bind 5mC of DNA, i.e., methyl-CpG-binding domain (MBD) (represented by MeCP2, MBD1, MBD2, and MBD4 proteins), Set and RING-associated (SRA) domain (including UHRF1 and UHRF2), and methyl-CpG binding Cys_2_His_2_ Zinc finger (C2H2 ZF) motifs (represented by Kaiso, ZBTB4, and ZFP57)^53^ (Fig. 2). Structural studies reveal that these domains use distinct physicochemical mechanisms to specifically recognize methylated CpG (mCpG) dinucleotides: the interaction between the MBD domain of MeCP2 and methylated CpG is driven by hydration of the major groove of methylated DNA rather than cytosine methylation itself;^54^ SRA domain of UHRF1 flips 5mC out of the DNA helix and accommodates it in a binding pocket with planar stacking, hydrogen bonding, and van der Waals interactions;^55–57^ ZF motifs of Kaiso recognize mCpG sites through hydrophobic and methyl CH···O hydrogen-bonding interactions.^58,59^ Functionally, MeCP2, MBD1, or MBD2 recognizes methylated CpG-island promoters and subsequently recruits histone deacetylases (HDACs) and histone H3K9 methyltransferases (SUV39H1 and SETDB1) through transcription repression domains (TRDs), resulting in transcriptional gene silence and heterochromatin formation.^60^ Additionally, MeCP2 is implicated in the translation of CpG methylation in gene-body regions into alternative splicing,^61^ and can specifically recognize hydroxymethylated CA repeats to prevent nucleosome deposition and regulate the transcription of CA repeat–enriched genes.^62^ MBD4 has a unique C-terminal glycosylase domain capable of correcting the mC→T mutation which is one of the primary sources of somatic mutation caused by spontaneous deamination of 5mC.^63–65^ UHRF1 (Ubiquitin-like with plant homeodomain and RING finger domains 1) recognizes hemimethylated DNA and catalyzes ubiquitylation of histone H3 lysine 18 (H3K18) and/or H3K23, providing a docking site for DNMT1 that faithfully propagates the DNA methylation patterns following replication.^66,67^ Intriguingly, UHRF2 preferentially binds to 5hmC via its SRA domain, and subsequent allosteric activation of its E3 ligase activity by 5hmC catalyzes K33-linked polyubiquitination of X-ray repair cross-complementing protein 1, which in turn instructs completion of DNA demethylation by TDG-mediated base excision repair.^68,69^ Kaiso and ZBTB4 are members of the BTB/POZ transcription factor family and can attract corepressor complexes, such as NCoR, SMRT, and Sin3/HDAC, via BTB/POZ domain to repress gene transcription.^70–72^ ZFP57 possesses a KRAB domain able to interact with KRAB-associated protein 1 (KAP1; also known as TRIM28) co-repressor complex and functions as a master regulator of genomic imprinting to regulate allelic expression of the imprinted genes.^73,74^

YT521-B homology (YTH) domain can read m^6^A of mRNA, including YTH domain family 1–3 (YTHDF1-3) and YTH domain-containing 1–2 (YTHDC1-2) proteins^75^ (Fig. 2). Biophysical studies of the YTH domains of YTHDF2 and YTHDC1 shows that aromatic cages (formed by Trp486, Trp432, and Trp491 in YTHDF2; Trp377 and Trp428 in YTHDC1) contribute to m^6^A recognition and binding through the cation–π interactions between the N6-methyl moiety and the side chains of the aromatic residues.^76–78^ All of the five YTH proteins except YTHDC2 contain intrinsically disordered regions (IDRs) and undergo liquid-liquid phase separation (LLPS) in the presence of mRNAs with multiple m^6^A signals, forming nuclear and cytoplasmic condensates (e.g., nuclear YTHDC1-m^6^A condensates (nYACs); cytosolic P-bodies, stress granules, and neuronal RNA granules), which is crucial in the control of fate and function of the m^6^A-modified mRNAs.^79,80^ YTHDC1 is a nuclear m^6^A reader that controls alternative splicing, alternative polyadenylation, nuclear export, and stability of m^6^A-modified mRNAs.^79,81^ In addition, YTHDC1 is implicated in the regulation of gene transcription and transposon silence by readout m^6^A signal of chromatin-associated noncoding regulatory RNAs (e.g., long non-coding RNA X-inactive specific transcript and enhancer RNAs) and transposon-derived RNAs (e.g., intracisternal A-type particle, ERVK and LINE1 RNAs).^81–84^ YTHDC2 possesses RNA helicase activity that can promote translation and degradation of m^6^A-modified mRNAs by resolving secondary structures and cooperating with the 5ʹ→3ʹ exoribonuclease XRN1, respectively.^85–87^ Unlike other members of the YTH family that preferentially bind to m^6^A sites, YTHDC2 weakly binds to m^6^A and possesses other RNA-binding domains besides the YTH domain,^88^ and recent studies argue that the role of YTHDC2 in germ cell development is independent of m^6^A recognition,^89,90^ thus raising doubt about the biological relevance of its m^6^A-reading activity. Earlier studies proposed that each YTHDF protein mediates different effects on m^6^A-modified mRNAs: YTHDF1 stimulates translation through interacting with the translation initiation factor eIF3;^91^ YTHDF2 promotes degradation by recruiting CCR4–NOT deadenylase complex and subsequent deadenylation,^92^ or by facilitating RNase P/MRP complex-mediated endoribonucleolytic cleavage when the m^6^A-modified mRNAs contain HRSP12-binding site (an adaptor) and RNase P/MRP cleavage site,^93^ or by interacting with UPF1 to promote decapping and subsequent 5ʹ→3ʹ exoribonucleolytic cleavage;^94^ YTHDF3 has both translation and decay effects via cooperating with YTHDF1 and YTHDF2.^95^ In addition, YTHDF1 and YTHDF3 (but not YTHDF2) promote m^6^A-mediated stress granule formation in osteosarcoma (U2OS) cells,^96^ while YTHDF2 and YTHDF3 (but not YTHDF1) mediate the localization of the m^6^A-modified mRNAs to neurites.^97^ However, earlier and especially two later studies challenged the view of distinct function, and proposed that all three YTHDF proteins function similarly and act redundantly to accelerate the decay of m^6^A-modified mRNAs, with no direct effect on translation.^92,98–100^ This is consistent with the fact that the three YTHDF paralogs show high sequence identity. The role of YTHDF1 in the regulation of mRNA stability is confirmed by multiple studies, and the effect of YTHDF3 is linked to the other two YTHDF proteins, therefore, one focus of the debate is the translation-stimulating function of YTHDF1. Further exploration and more data are required to clarify whether YTHDF proteins function in similar or distinct ways or a unified explanation will be found to reconcile the contrasting observations in the future.

Similar to the role of aromatic cages in YTH domains, variant aromatic cages consisting of two to four aromatic residues (Phe, Tyr, or Trp, and occasionally His) are involved in the specific interactions with methyl-lysine motifs of proteins through the cation-π interactions between the methylated ammonium group and the aromatic cage.^101,102^ There are nine types of aromatic-cage-containing domains capable to recognize methylated lysines, i.e., Tudor, chromo, malignant brain tumor (MBT), proline-tryptophan-tryptophan-proline, tryptophan-aspartate 40 (WD40), plant homeodomain (PHD), ankyrin repeats, bromo-adjacent homology, and cysteine-tryptophan^103^ (Fig. 2). Among these, Tudor, PHD, and WD40 domains are also capable of accommodating methyl-arginine motifs.^104^ The effects of histone and nonhistone protein methylations are versatile and context-dependent, and different readers with these domains mediate different biological outputs. For example, TAF3, a subunit of the basal transcription factor TFIID, utilizes PHD domain to bind H3K4me3 at gene promoters and stimulate RNA polymerase II-mediated transcription.^105^ Heterochromatin protein 1 (HP1) recognizes H3K9me2/3 via the chromo domain to instruct heterochromatin formation, contributing to gene transcriptional silence and stabilization of H3K9 methyltransferases and demethylases.^106,107^ Di-methylation of p53 at K370 (K370me2) can be recognized by the Tudor domains of 53BP1 or PHF20, promoting transcriptional activity and stability of p53, respectively;^108,109^ whereas mono-methylation at K382 (K382me1) is read by the triple MBT repeats of the chromatin compaction factor L3MBTL1, inhibiting p53-mediated transcriptional activation.^110^

Currently, such conserved methyl-group binding domains dedicatedly for 6 mA in DNA and m^5^C in mRNAs have yet to be identified. Interestingly, the YTH domain of YTHDC1 can efficiently bind to 6 mA in single-stranded and lesion-containing double-stranded DNAs in vitro.^111,112^ As YTHDC1 is recruited by m^6^A in RNA hybridized with DNA at DSB sites and stimulates homologous recombination-mediated repair of DSBs by stabilizing DNA:RNA hybrids, it is tempting to hypothesize that YTHDC1 may be recruited to DNA damage sites by 6 mA in DNA in vivo and play a role in the damage repair and maintenance of genome stability.^111,113^ However, no evidence was found that YTHDC1 could localize to ultraviolet-induced damage sites, making the hypothesis suspicious.^114^ Aly/REF export factor (ALYREF), a reader of m^5^C in mRNA, promotes nuclear export of the modified mRNAs.^21^ Although no apparent methyl-group binding domain was found in ALYREF, sequence alignment analysis using MBD and YTH family proteins as references along with experimental validation identified a conserved amino acid (K171) crucial for the specific binding,^21^ suggesting a potential conserved methyl-group binding domain might exist when more readers of m^5^C are available.

A different group of methyl signal readers uses common DNA or RNA binding domains to preferentially bind to methylated DNA or RNA in a sequence-dependent manner, such as Rel-homology domains (RHDs) and homeodomains for DNA, K homology (KH) domains, Arg-Gly-Gly repeat (RGG) domains, and cold shock domains (CSD) for RNA. NFAT (RHD) transcription factors and many members of the extended homeodomain (e.g., homeodomain, POU, and NKX) transcription factor family prefer to bind to CpG-methylated DNA sequences through direct hydrophobic interactions between the homeodomains and the *C*^5^-methyl group.^115^ Insulin-like growth factor 2 mRNA-binding proteins (IGF2BPs; including IGF2BP1-3) use KH domains to recognize m^6^A-modified mRNAs, which promotes mRNA stability by preventing degradation in the P-body or boosting storage in stress granules under stress conditions and facilitates mRNA translation by shuttling to ribosome fractions during recovery from stress.^116^ Proline-rich coiled-coil 2A (Prrc2a) utilizes the GRE domain (enriched in glycine, arginine, and glutamic acid) to compete for binding of m^6^A-modified mRNAs with YTHDF2 and stabilizes the *Olig2* transcripts which are involved in oligodendrocyte specification and myelination.^117^ Fragile X mental retardation protein (FMRP) has three KH and one RGG domains and prefers m^6^A-modified mRNAs, which modulates the nuclear export, translation, and stability of the targets by interacting with CRM1, YTHDF1, and YTHDF2, respectively.^118–121^ FMRP can also act as an m^5^C reader that preferentially binds to DNA:RNA hybrids containing m^5^C-modified mRNAs at DSB sites, and the KH RNA binding domain of FMRP is required for the functional readout of the methyl signal, in which FMRP promotes completion of homologous recombination repair by facilitating TET1-mediated demethylation of m^5^C.^22^ Similarly, RAD52 recognizes m^5^C mRNA in DNA:RNA hybrids at DNA damage sites and promotes homologous recombination-mediated DSB repair by recruiting RAD51.^23^ The potential domain of RAD52 responsible for m^5^C recognition has yet to be identified. Y-box binding protein 1 (YBX1) uses a CSD domain to bind m^5^C-modified mRNAs through CH–π interactions between the indole ring of Trp65 and the methyl group of m^5^C, which stabilizes the mRNAs by recruiting an mRNA-stability maintainer ELAVL1.^122^ Interestingly, YBX1 plays a role in regulating the stability of m^6^A-modified mRNA targets via interaction between its CSD domain and IGF2BPs.^123^ These studies point out the dual roles of certain common RNA-binding proteins (e.g., FMRP and YBX1) in the functional interpretation of both m^5^C and m^6^A signals in mRNAs.

A distinct subgroup of readers (also called indirect readers) binds methylated substrates using common domains upon methylation-induced structural shift and exposure of the specific binding motifs, which is best demonstrated in the RNA field known as “m^6^A structural switch”. Several nuclear ribonucleoproteins (HNRNPs) including HNRNPC, HNRNPG, and HNRNPA2B1 belong to this subgroup, and function in transcript processing, including splicing.^124–126^ They use RNA recognition motifs or RGG domains to bind exposed recognition sites due to destabilized hairpin stem around the m^6^A:U pair or other unknown physicochemical mechanisms.^125–128^ Although IGF2BPs can directly recognize m^6^A via a GGAC motif, there is evidence that they can bind different RNA targets through the “m^6^A structural switch” mechanism.^129^ It is conceivable that any RNA-binding protein could benefit from an m^6^A structural switch when its binding motifs are near or overlapping with m^6^A sites. However, it is often difficult to clearly distinguish between direct binding and RNA-structure-dependent binding, as both mechanisms have been seen for proteins including HNRNPA2B1 and IGF2BPs.^126,129,130^ Since methylation can alter the local structure of DNA and proteins, such a structural switch mechanism might also be applied to potential indirect readers of DNA and protein methylation.

#### Beyond readers

Methylation of DNA, RNAs, or proteins can exert biological effects independent of readers. In contrast to readers attracted by methylation, methylation can directly repel binding proteins that prefer unmethylated targets. The most important protein repelled by DNA cytosine methylation is a C2H2 ZF protein, CCCTC-binding factor (CTCF). CTCF is implicated in a variety of regulatory processes, including chromatin architecture, transcriptional activity, alternative splicing, and alternative polyadenylation.^131–135^ The binding of most major classes of transcription factors, including bHLH-, bZIP-, and ETS-families, is inhibited by DNA methylation-mediated steric hindrance.^115^ ZF-CxxC domain-containing proteins, such as CXXC finger protein 1, histone lysine transferase MLLs, and histone lysine demethylase JHDM1A/B (KDM2A/B), recognize unmethylated CpG dinucleotides to regulate epigenetic modification, while methylation blocks their binding to DNA due to a steric clash between the methyl group and the protein backbones.^136–139^ The structural and binding analysis identified some RNA-binding proteins repelled by m^6^A in mRNAs, including stress granule proteins G3BP1/2, pluripotency regulator LIN-28 homolog A (LIN28A), and EW RNA binding protein 1 (EWSR1), and the further study confirmed that m^6^A can modulate mRNA stability and turnover by repelling G3BP1.^118,129^ m^6^A deposited by nematode METT-10 (the ortholog of mammalian METTL16) at the 3′ splice site represses proper splicing and protein production of the targeted mRNAs through physically blocking the binding of the essential splicing factor U2AF35, and this mechanism of splicing regulation is conserved in mammals.^140^ Although in vivo mRNA targets of mammalian METTL16 remain to be characterized, the finding highlights the biological significance of m^6^A-mediated direct inhibition of protein binding. Recognition and binding of unmodified lysine or arginine of histone proteins including H3K4 and H3R2 are performed by a separate group of PHD domains, including those of BRAF35–HDAC complex protein (BHC80), autoimmune regulator, tripartite motif-containing protein 24 (TRIM24) and DNMT3L, KDM5A, UHRF1, and DPF3b. These PHD domains replace the aromatic cages with a combination of acidic and hydrophobic residues, facilitating hydrogen-bonding interactions with the unmethylated H3K4 or H3R2.^141^ In contrast, methylation of these sites decreases hydrogen-bonding capacity and disrupts binding by the PHD domain-containing proteins. Along with proteins repelled by DNA methylation, factors repelled by histone methylation are important components of the chromatin-based regulation network of gene transcription. Methylation of nonhistone proteins can directly repress their interaction with other proteins, playing a role in the regulation of signaling transduction and gene expression. For example, methylation of MAPK kinase kinase 2 (MAP3K2) at Lys260 prevents the binding of protein phosphatase 2A complex (a key negative regulator of the MAPK pathway), resulting in elevated MAP3K2 signaling and promotion of Ras-driven cancer.^142^ Mono-methylation of a crucial lysine within the nuclear export signal sequence of YAP, a key effector of the Hippo pathway, blocks its interaction with the nuclear exporter CRM1, which results in the retention of YAP in the nucleus and stimulates YAP-mediated transcription activity and tumorigenesis^143^ (Fig. 4b). Methylation of transcriptional coactivator bromodomain-containing protein 4 (BRD4) at Lys99 compromises its interaction with transcription factor E2F1, leading to reduced expression of translation-related genes and decreased total mRNA translation.^144^

Furthermore, if the nonhistone proteins are nucleic acid-binding proteins, methylation can directly affect their DNA/RNA binding affinity positively or negatively. For instance, methylation of two lysines of p65 (a subunit of NF-κB) enhances the binding of p65 to targeted DNA sites by producing new hydrophobic contacts, resulting in the activation of downstream genes.^145^ Methylation of the RGG3 domain of Ewing’s sarcoma abolishes its interaction with the substrate DNA containing G-quadruplex structure, while retaining its ability to bind the mutant counterpart lacking the G-quadruplex structure.^146^ Methylation of the coiled-coil domain of p54^nrb^, a subunit of paraspeckle-associated protein complexes, prevents the binding of p54^nrb^ to mRNAs with double-stranded RNA structure, which reduces paraspeckle-mediated nuclear retention of the mRNAs.^147^ The RNA binding activity of cellular nucleic acid binding protein, a zinc-finger protein that binds structured RNAs, is inhibited upon arginine methylation.^148,149^ These cases imply that protein methylation-mediated interfering binding of nucleic acids is associated with DNA/RNA higher structure, which may be due to interfered hydrogen bonding or introduced steric clashes.

Methylation can change the local or global structure of methylated DNA, RNAs, and proteins, which directly mediates the effects of methylation. DNA cytosine methylation stabilizes the double helix structure and in turn slows down the DNA unwinding, replication, and transcription.^150^ Furthermore, cytosine methylation causes profound alteration in the conformation of both nucleosomal and linker DNA, resulting in enhanced contacts between the 5mC-modified DNA and histone proteins.^151,152^ Such more stable and compact nucleosomes restrict DNA accessibility and facilitate the formation of repressive chromatin. DNA methylation also modulates the formation of certain non-canonical DNA (non-B DNA) structures including G-quadruplexes, which affects gene expression.^153^ Methylation in the mRNA coding sequence (CDS) including m^5^C and m^6^A can regulate codon-anticodon interactions, influencing translation efficiency in a codon-specific manner.^85,154^ Moreover, the three-dimensional (3D) structures of mRNAs can be altered or stabilized upon methylation, which regulates their stability, localization, splicing, and translation efficiency. The above-mentioned “m^6^A structural switch” is a good example in which methylation-induced structural alteration matters. A transcriptome-wide study showed m^5^C may commonly compromise the mRNA translation, which is likely associated with the stabilized secondary structures upon methylation by facilitating base stacking and enhancing the hydrogen-bonding strength with guanosine.^17,155^ Methylation of histone H3K79 and H4K20 alters nucleosomal surface and higher-order chromatin structure.^156,157^ Specifically, mono-methylation of H4K20 directly stimulates chromatin openness by interfering with chromatin folding, thereby promoting the transcription of housekeeping genes.^156^

Protein methylation, especially lysine methylation, competitively inhibits other post-translational modifications of the same residues, such as acetylation, ubiquitination, and crotonylation.^158^ There are at least 29 types of lysine modification across 219 species including humans, and their dysregulation is involved in abnormal biological processes and human diseases.^159^ For example, tri-methylation of H3K9 and H3K27 blocks the acetylation of the two sites and keeps the genes from activation.^160,161^ Mono-methylation of lysine 120 on histone H2B (H2BK120) in cancer cells prevents the ubiquitination of H2BK120 and down-regulates transcription of downstream tumor-suppressor genes.^162^ Nonhistone protein methylation (e.g., K372 of p53 and K302 of estrogen receptor α protein) can stabilize the methylated proteins by inhibiting polyubiquitination-dependent proteolysis.^163,164^ The biological significance of the switch between methylation and the other modifications (besides ubiquitination and acetylation) at specific lysine residues is poorly understood. In addition, a recent study showed that di-methylation of the autophagy initiation protein ULK1 at R170 directly stimulates its autophosphorylation of spatially closed T180, which activates ULK1-mediated hypoxic stress adaptation.^165^ These progresses indicate complex interactions between methylation and other post-translational modifications within proteins.

## Methylation in neurological disorders, cancer, and aging

### Neurological disorders

The development and function of the nervous system require cell-type-specific precise control of methylation pattern and readout at DNA, RNA, and protein dimensions, which is involved in the proliferation and differentiation of neural precursors, neuronal maturation, gliogenesis, synaptogenesis, and common brain physiology. Disruption of methylation patterns or factors has been linked to various human neurological disorders, such as Rett syndrome (RTT) and Fragile X syndrome (FXS).

#### Rett syndrome

**Fig. 3:**
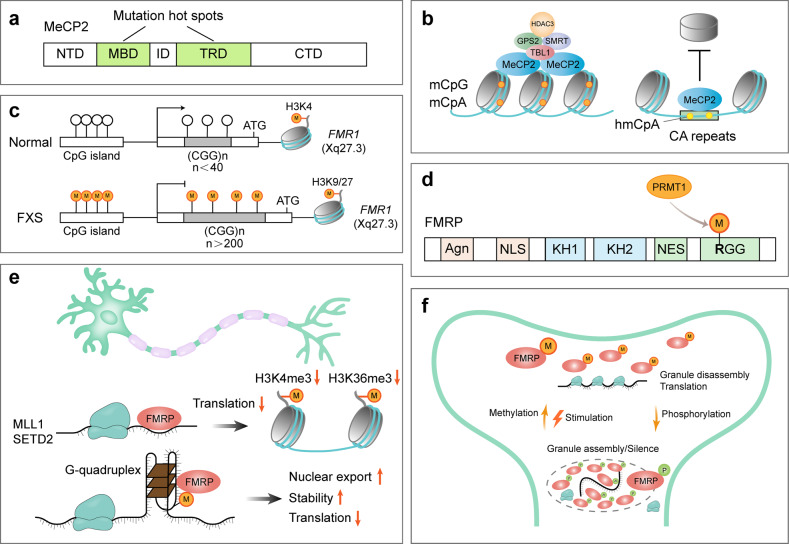
Function of *MeCP2* and *FMR1* and their mutations in RTT and FXS respectively. **a** The majority of RTT-causing mutations are located in the methyl-CpG-binding domain (MBD) and transcriptional repression domain (TRD) of MeCP2. ID, intervening domain; NTD, N-terminal domain; CTD, C-terminal domain. **b** Molecular functions of MeCP2: MeCP2 recognizes mCpG and mCpA and recruits NCOR-SMRT co-repressor complex to compact chromatin and suppress transcription; MeCP2 binds the hydroxymethylated CA repeats and protects them from nucleosome invasion. Both functions are abolished upon RTT-causing mutations. **c** CGG trinucleotide repeat expansion (>200 repeats) in the 5′-untranslated region of the *FMR1* gene causes DNA hypermethylation and histone methylation shift, which silences the *FMR1* gene in FXS patients. **d** Multiple important domains of FMRP, including a tandem Agenet (Agn) domain that binds DNA and other proteins, a nuclear localization sequence (NLS), a nuclear export sequence (NES), and several RNA- binding domains (KH1, KH2, and RGG box). PRMT1 performs arginine methylation of RGG motifs within FMRP. **e** FMRP regulates the histone methylation states by modulating the translation of writers (MLL1 and SETD2), and binds a fraction of m^6^A-modified mRNAs (probably with a G-quadruplex structure) to modulate their nuclear export, stability, and translation, which is implicated in the regulation of neural differentiation, development, and function. **f** Methylation and phosphorylation of FMRP have opposing effects on the neuronal granule assembly and activity-dependent translation through modulating FMRP-mediated phase separation

Unlike symmetrical CpG methylation that is maintained by DNMT1 during genome replication, asymmetrical non-CpG methylation is lost in replicating cells.^166^ Since post-mitotic neurons do not undergo replication, they accumulate exceptionally high levels of non-CpG methylation alongside the CpG methylation, most prevalently in CpA dinucleotides (mCpA).^4,167^ The deposition of mCpA is performed by DNMT3A at gene bodies of lowly expressed genes during early life in the brain and recruits the reader MeCP2 to repress transcription of the targeted genes in the adult brain^168^ (Fig. 3b). Consistently, the evolutionary analysis revealed that non-CpG methylation is confined to vertebrates and enriched within a highly conserved set of developmental genes silenced in adult brains, and MeCP2 originated at the onset of vertebrates, suggesting the emergence of non-CpG methylation and its reader may facilitate the evolution of sophisticated cognitive abilities of vertebrate lineage.^169^ As a result, loss-of-function mutations in the *MECP2* gene cause a severe neurological disorder known as RTT. The MeCP2 protein consists of an N-terminal domain, an MBD that recognizes methylated cytosine, an intervening domain (ID), a TRD able to recruit HDAC3-containing NCoR/SMRT co-repressor complex, and a C-terminal domain (Fig. 3a). RTT-causing mutations are largely confined to the MBD and TRD domains, supporting MeCP2 serves as a bridge between methylated DNA and the co-repressor complexes.^170,171^ RTT is characterized by an initial normal early development followed by progressive neurological dysfunction and developmental regression, which could be explained by the postnatal accumulation of non-CpG methylation and MeCP2.^172^ The binding of MeCP2 to DNA is correlated with the number of methylated cytosines, therefore, long genes with more methylcytosines, especially mCpA, are preferentially silenced by MeCP2 in neurons.^173^ Mice expressing a chimeric MeCP2 protein containing the DNA-binding domain of MBD2 that cannot bind mCpA develop severe RTT-like phenotypes, while mice with about half MeCP2 expression level show only very mild behavioral phenotypes, suggesting the irreplaceable functional significance of mCpA cannot be simply explained by doubling the abundance of methylcytosines in neurons; instead, the distribution of mCpA that shows more cell-type-specific than mCpG is crucial.^174,175^

In contrast, MeCP2 can also directly mediate gene activation through interacting with co-activator complexes at the promoters of targeted genes. Particularly, ~85% of thousands of genes can be positively regulated in the hypothalamus of mice by MeCP2.^176^ The exact mechanism of MeCP2-mediated gene activation remains unclear, and three models have been proposed. (1), cyclic AMP‑responsive element‑binding protein 1(CREB1) was identified as a MeCP2-interacting co-activator, and their co-occupancy at the promoter of an activated targeted gene, *somatostatin* (*Sst*), was confirmed.^176^ However, RTT-associated mutations have not been reported to impact the interaction between MeCP2 and CREB1. (2), HDAC3 recruited at promoters by MeCP2 can deacetylate the transcription factor forkhead box O3 (FOXO3) to stimulate the transcription of a subset of neuronal genes, including *brain-derived neurotrophic factor* (*BDNF*) gene.^177^ The RTT-causing mutation R306C in the TRD domain of MeCP2 inhibits the recruitment of HDAC3 and FOXO3, leading to the downregulation of targeted genes.^177^ (3), the transcription factor 20 (TCF20) complex interacts with the MBD-ID domain of MeCP2, which may regulate the transcription of downstream genes, such as *BDNF*.^178^ The interaction between MeCP2 and TCF20 complex is disrupted by the RTT-causing missense mutations in the MBD-ID region and by a missense mutation in a subunit of the TCF20 complex that was found in a patient with RTT-like syndrome.^178^ Although the latter two models seem to be compatible with mutation studies, all the current MeCP2-mediated gene activation models place MeCP2 at the transcription start sites or promoters where the level of both CG and non-CG methylation is low and cannot explain the essential role of the gene-body enrichment of non-CG methylation in the pathogenesis of RTT.^174,179^ More complex, MeCP2 mediates both negative and positive transcriptional regulation of targeted genes, such as one of the most and best-studied downstream genes, *BDNF*. Specifically, MeCP2 represses promoter III–dependent transcription of exon III–containing *BDNF* mRNA in the absence of neuronal activity, while the release of MeCP2 from promoter III upon phosphorylation is a prerequisite for de-repression.^180^ This differential regulation may be related to the location of the methylation and MeCP2 binding, resulting in different adjacent interacting proteins recruited by the surrounding DNA motifs. In addition to mCpG and mCpA, MeCP2 can recognize hydroxymethylated CpA (hmCpA) repeats through Arg^133^ and repel nucleosomes^62^ (Fig. 3b). The Arg^133^ is a potent RTT-causing mutation site, and loss-of-function mutation of MeCP2 alters chromatin architecture and genome-wide transcription of CpA repeat-enriched genes.^62^ The interaction of MeCP2 with hydroxymethylated DNA has a different thermodynamic signature, compared to that of methylated or unmethylated DNA.^181^ These studies show the complex regulatory role of MeCP2 in gene transcription through read mCpG, mCpA, and hmCpA signals.

Besides transcriptional control, it has been reported that MeCP2 binding of mCpG at the gene bodies can promote exon recognition in the alternative mRNA splicing process via recruitment of HDACs and subsequently altered DNA polymerase II elongation rate in two human non-neuron cell lines.^61^ Indeed, hundreds of aberrant splicing events occur in the cortex of *Mecp2* knockout mice, including genes critical for synaptic plasticity (*Gria2*, *Nrxns*, and *Nlgn1*).^182^ However, the mechanism identified in the non-neuron cell lines is not responsible for the altered splicing in the cortex of the RTT mouse model. Instead, MeCP2 interacts with several regulators of RNA splicing, including Y box-binding protein 1 (YB-1), lens epithelium-derived growth factor p75 (LEDGF/p75), or RNA-binding fox-2 (RBFOX2).^182–184^ The TRD domain of MeCP2 is involved in the interaction with YB-1 and LEDGF, and binding of methylated DNA is not required for their interaction, leaving the role of DNA methylation in the MeCP2-mediated alternative splicing unresolved.^183,184^ In contrast, the MBD and ID domains of MeCP2 are implicated in association with RBFOX2, which promotes the formation of large assemblies of splicing regulator (LASR) condensates in a DNA methylation-dependent manner through the LLPS property of MeCP2.^182,185^ Furthermore, RTT-causing missense mutations within MBD compromise the formation of MeCP2/RBFOX/LASR condensates.^182^ These results indicate MeCP2 can function as a bridge to link methylated DNA and mRNA splicing modulators. Future study using *Dnmt3a* conditional knockout model or the chimeric MeCP2 protein that can distinguish non-CG methylation from CpG methylation in neurons is promising for comprehensively understanding the mechanism of methylation-mediated splicing regulation in regards to specific methylation types, which may be implicated in the pathogenesis of RTT.^168,174^

#### Fragile X syndrome

FXS, an X-linked neurodevelopmental disorder, is a leading inherited form of intellectual disability and autism spectrum disorder (ASD), afflicting ~1 in 4000 males and 7000 females. Nearly all cases of FXS are caused by CGG trinucleotide repeat expansion (>200 repeats) in the 5′-untranslated region of the *fragile X mental retardation 1* gene (*FMR1*), leading to transcriptional silence and loss of the gene product FMRP (Fig. 3c). The mechanism underlying *FMR1* inactivation is of particular interest since *FMR1* reactivation can serve as a therapeutic strategy for FXS.^186,187^ Despite intensive research, the exact mechanism of the repeat expansion-induced gene silence remains unresolved.^188^ One important factor is the DNA cytosine hypermethylation of the *FMR1* promoter and the repeat region. Demethylation of the *FMR1* gene by DNMT inhibitors (e.g., azacitidine and decitabine) or CRISPR-mediated DNA demethylation reactivates the *FMR1* expression and rescues FXS neurons.^186,189–191^ Consistently, rare individuals with normal intelligence have a completely unmethylated or partially methylated mutated *FMR1* gene capable of producing FMRP proteins.^192–194^ The presence of expanded CGG-repeats in the 5′-untranslated region of the *FMR1* mRNA can stimulate the formation of DNA:RNA hybrid, which stalls RNA polymerase II (Pol II) transcription and causes gain of repressive histone marks including H3K9me2 and H3K27me3 and loss of activating histone mark H3K4me2^195,196^ (Fig. 3c). Combing the DNMT inhibitor (decitabine) with H3K9 or H3K27 HMT inhibitor (chaetocin or 3-deazaneplanocin A, respectively) potentiates the effect of reactivating treatment and prevents re-silencing, compared with decitabine treatment alone.^186,197^ This suggests a combination of DNA and repressive histone methylation mediates stable transcriptional silencing of the mutated *FMR1* gene. Since both DNA and histone methylation is stable and inheritable during cell divisions, the methylation pattern can be maintained in the absence of the initial stimulus.^198–200^ Strikingly, removal of the CGG repeat from FXS patient-derived cells by genome editing can stimulate extensive demethylation of the upstream CpG island within the *FMR1* promoter, shift repressive histone methylation to active modification, and initiate *FMR1* transcription.^201^ This suggests CGG repeat expansion is not only required for the establishment of the silence state of *FMR1* but also involved in the maintenance of the silence. A recent genome-wide loss-of-function genetic screening uncovered 155 candidate genes predicted to be involved in the maintaining silence of an *FMR1* reporter in the haploid and FXS patient-derived pluripotent stem cells (PSCs), including transcriptional co-repressor *ZNF217*, chromatin remodeling factor *SMARCD1*, and succinate-metabolism factor *C6orf57* (involved in the regulation of α-KG-dependent histone demethylation process).^202^ Among these, only *DNMT1* disruption resulted in robust and partial expression of *FMR1* mRNA, implying different repressive mechanisms exist to function redundantly for stable silence of the *FMR1* gene.^202^

FMRP is a widely expressed RNA-binding protein and plays an important role in nearly all aspects of brain development and function, including neurogenesis, neuronal maturation, and excitability^203^ (Fig. 3d). Initially, it was revealed that FMRP function as a translation repressor by stalling ribosome translocation, and recent studies showed it also regulates alternative splicing, poly(A) tail length, localization, and stability of mRNAs.^119,121,204–206^ More than 1000 mRNAs in the brain are targets of FMRP, among these, ~20 of which are histone methylation modifiers, e.g., MLL3 (H3K4me1/2 writer) and SETD2 (H3K36me3 writer) that regulate transcription and alternative splicing of genes related to neural function, respectively^207,208^ (Fig. 3e). The principle of target mRNA selection is a focus of the field. m^6^A modification within the mRNAs contributes to the target specificity. FMRP preferentially binds m^6^A-modified mRNAs to maintain the mRNA stability in adult mouse cerebral cortex and to promote nuclear export of the mRNAs that regulate mouse neural differentiation.^119,121^ YTHDF, the unique cytoplasmic YTH protein in *Drosophila*, regulates FMR1(the *Drosophila* FMRP homolog) target selection in an m^6^A-dependent manner, which represses the translation of key mRNAs implicated in axonal growth.^209^ A similar mechanism of indirectly choosing m^6^A-modified mRNAs might work in mammals as YTHDF2 can interact with FMRP in an RNA-independent manner.^119^ Moreover, since the m^6^A modification is more prevalent in the human brain than the mouse, m^6^A might contribute to human-specific mRNA targeting by FMRP.^210,211^ It should be noted that only a fraction of m^6^A-modified mRNA is recognized by human FMRP, suggesting m^6^A cooperates with other factors to define a subset of FMRP targets.^120^ One possibility is the FMRP-interacting protein, like the mechanism in *Drosophila*.^209^ Alternatively, m^6^A may cooperate with secondary RNA structure, such as G-quadruplex, to restrict FMRP specificity, which is supported by the co-localization of m^6^A and G-quadruplex-forming sequences and preference of FMRP for RNA G-quadruplex structure^212–214^ (Fig. 3e).

The recognition of the G-quadruplex structure is executed by the RGG domain of FMRP.^215,216^ Methylation of this domain compromises the interaction of FMRP with G-quadruplex-containing mRNAs and polyribosomes, facilitating translation^217–219^ (Fig. 3d). Coupling with phosphorylation, methylation/demethylation of FMRP protein regulates reversible neuronal granule assembly for activity-dependent translation control at the synapse^220^ (Fig. 3f). Collectively, the biological function of *FMR1* gene is associated with methylation status of chromatin, mRNA targets, and the protein per se. Deciphering the details of the underlying molecular mechanisms may provide the opportunity to develop new therapies for FXS.

### Cancer

**Fig. 4:**
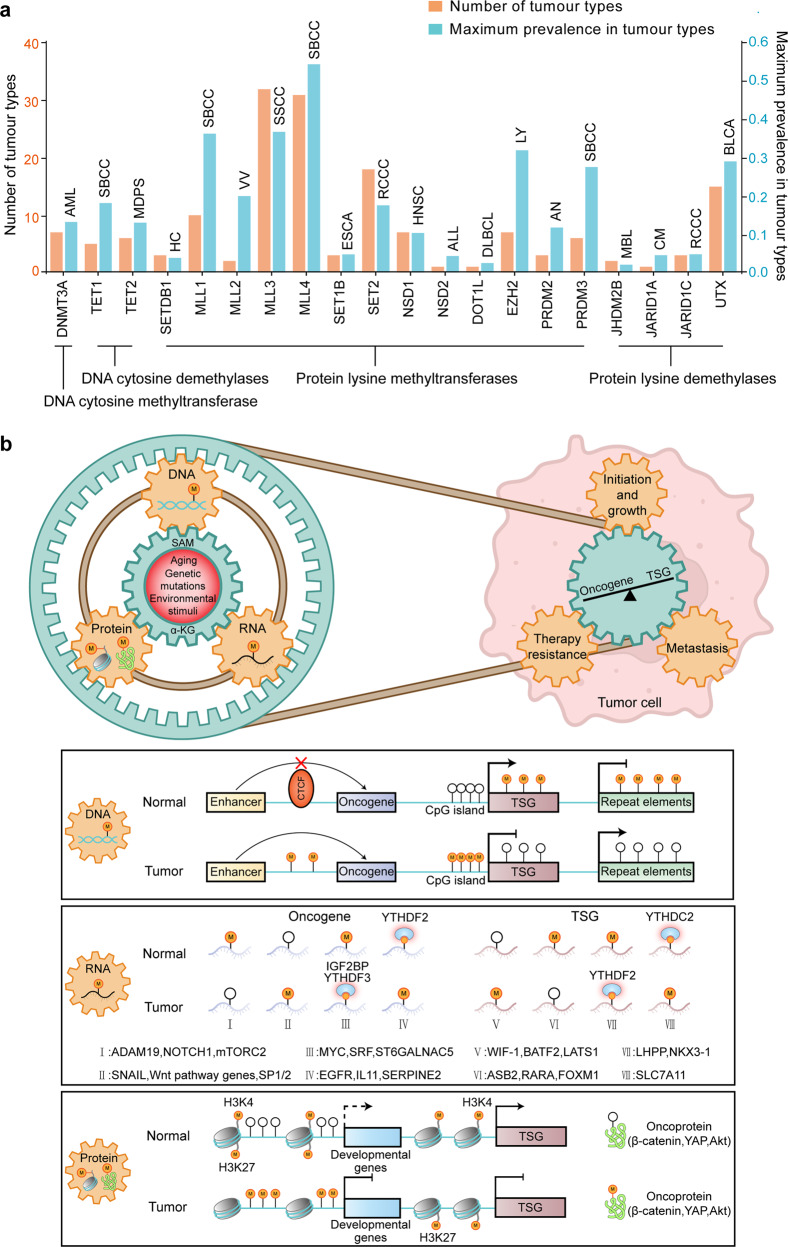
Mutational cancer driver methylation modifier genes and the role of methylation dysregulation across the central dogma in tumor initiation and progression. **a** Distribution of the prevalence of methylation modifiers with cancer driver mutations across 66 cancer types. All data are retrieved from IntOGen database. AML acute myeloid leukemia, SBCC skin basal cell carcinoma, MDPS myelodysplastic syndrome neoplasm, HC hepatic cancer, VV vulval cancer, SSCC skin squamous cell carcinoma, ESCA esophageal carcinoma, RCCC renal clear cell carcinoma, HNSC head and neck squamous cell carcinoma, ALL acute lymphoblastic leukemia, DLBCL diffuse large B cell lymphoma, LY lymphoma, AN anus cancer, MBL medulloblastoma, CM cutaneous melanoma of the skin, BLCA bladder cancer. **b** Methylation remodeling of DNA, RNA, histone, and nonhistone proteins contributes to tumor initiation and progression. Aging, genetic mutations, or environmental stimuli induce methylation remodeling of DNA/RNAs/proteins and causes oncogene activation and TSG silence. A gear set is used as a metaphor for the link between different methylation pathways and their roles in tumorigenesis. The common mechanisms that cause oncogene/TSG disturbance by methylation remodeling at DNA, RNA, and protein levels are recapitulated in the boxes

Cancer is a leading cause of death worldwide with almost 10.0 million deaths in 2020, and the global cancer burden is expected to increase by 47%, i.e., from 19.3 million cases in 2020 to 28.4 million cases in 2040.^221^ The increase in incidence is associated with an aging and growing population as well as changes in the distribution and prevalence of the cancer risk factors, such as excess body weight, physical inactivity, ionizing radiation, chronic infection, and certain environmental pollutants. The transformation of normal, healthy cells into lethal cancer cells with multiple hallmark capabilities, including sustaining proliferative signaling, resisting cell death, avoiding immune destruction, inducing/accessing vasculature, unlocking phenotypic plasticity, and activating invasion and metastasis, is enabled at least by genome instability/mutation and non-genetic alterations within the cells. Aberrant and adaptive DNA/RNA/protein methylation landscape contributes to every stage of cancer progression, from initiation to metastasis and treatment resistance (Fig. 4). The use of single-cell and spatial technologies has provided unprecedented insights into the mechanism of methylation in tumorigenesis.

#### Instability of genome and methylome in cancer

Cancer genomes accumulate different numbers and types of mutations (e.g., point mutations, copy number alterations, genomic rearrangements) in coding and non-coding sequences, due to various exogenous and endogenous DNA damaging agents, including ultraviolet, ionizing radiation, chemicals, replication errors, spontaneous hydrolytic reactions, and reactive oxygen intermediates.^222–224^ A relatively small proportion of mutations, known as drivers, contribute to tumorigenesis and are thus under positive selection with observed frequency and patterns of mutations deviating from that of expected neutral mutations (known as passengers).^225^ Dysregulation of DNA, RNA, histone, and nonhistone protein methylation contributes to genome instability/mutations and cancer progression. DNA cytosine methylation can directly promote genetic mutations by spontaneous hydrolytic deamination of 5mC, which leads to C→T transition mutations and is a common cause of somatic point mutations in tumor suppressor genes (TSGs) including *TP53*.^223,226^ Indirectly, promoter methylation-induced silence of DNA repair genes (e*.*g., *MLH1*, *BRCA1*, *MGMT*) compromises the ability to repair DNA damages and results in genome instability and hypermutation during tumor initiation and progression.^227–229^ Loss of DNA methylation at transposable elements reactivates their activity, and subsequent random insertion into the genome potentiates cancer-driver mutations.^230^ Dynamic mRNA modifications including m^6^A and m^5^C are involved in homologous-recombination-mediated DNA repair through the formation and resolution cycle of R-loops.^22,23,113^ Deficiency of the mRNA methylation or disruption of the dynamics compromises the repair process or induces new damages due to the persistence of R-loops, respectively.^22,23,113,231^ Elevation of m^6^A modification in human cancers promotes telomere shortening and genomic instability through degradation of m^6^A-modified *HMBOX1* mRNAs.^232^ Methylated histones often provide docking sites for repairing proteins at damaged sites. For example, H3K9me3 and H3K4me3 recruit TIP60 and RIF1 for homology-dependent and non-homologous end-joining (NHEJ) repair respectively.^233,234^ An increasing number of DNA repair proteins or regulators have been identified to be substrates of PRMTs, especially PRMT1 and PRMT5. PRMT1 methylates USP11, MRE11, and 53BP1 while PRMT5 methylates RUVBL1, FEN1, RAD9, and TDP1 to facilitate DNA repair and maintain genome stability.^235,236^ In addition, PRMT5 methylates DDX5 and the carboxy-terminal domain of Pol II to resolve the R-loop and prevent DNA damage.^235^ Therefore, maintenance of proper methylome at DNA, RNA, and protein levels is required for genome integrity.

The pattern of DNA methylation in most cancers is characterized as genome-wide hypomethylation accompanied by focal hypermethylation at CpG islands in promoters.^237^ The exceptions, including follicular thyroid cancer, acute lymphoblastic leukemia, and acute myeloid leukemia, only show a hypermethylation phenotype without global hypomethylation.^238,239^ Genes susceptible to promoter CpG-island hypermethylation in adult human cancers are bivalently marked with H3K4me3 and H3K27me3 in embryonic stem cells. The bivalent marks let lineage-controlling developmental genes in a repressed and “transcription-ready” state, while DNA hypermethylation, probably a result of loss of H3K4me3/H3K27me3 bivalency in cancer, locks the silenced state and reduces regulatory plasticity, promoting the maintenance of stem cell features and carcinogenesis.^240–242^ In addition, DNA methylation-induced transcriptional silence frequently occurs in a number of TSGs involved in cancer-related cellular pathways, such as DNA repair (*MLH1*, *BRCA1*, *MGMT*), cell cycle (*CDKN2A/B*, *Rb*), p53 network (*TP73*, *HIC1*), Ras signaling (*RASSF1A*), and apoptosis (*TMS1*, *DAPK1*).^243^ Global hypomethylation occurs primarily in lamina-associated and late-replicating regions, maybe a result of inefficient methylation maintenance during excess mitotic cell division.^244,245^ The oncogenic role of global hypomethylation has been attributed to transcriptional activation of transposable elements and/or oncogenes involved in the regulation of cell proliferation, angiogenesis, immortality, metastasis, and tumor suppressor pathways.^246,247^ However, this classical oncogenic view has been challenged by a study suggesting that global hypomethylation and associated topological alterations have a tumor-suppressive role in colorectal cancer through inhibiting stemness and invasion programs and activating antitumor immunity genes.^248^ The existence of cancer types without global hypomethylation indicates that global hypomethylation maybe not a prerequisite for carcinogenesis.^238,239^ Moreover, DNA methylation is required for maintaining the integrity of higher-order genome architecture, and the hypomethylation treatment caused a similar tumor-suppressive topological genome reorganization in human colon cancer cells.^248,249^ It is important to learn more about how cancer cells balance the oncogenic and tumor-suppressive effects of global hypomethylation.

Methylome has high intrinsic plasticity that is needed for the differentiation of hundreds of cell types in our body with a unique same genome. Operating at the interface between the genome and the environment, it readily changes upon environmental stimuli, which can precede oncogenic mutations and predispose cells to driver mutations through above-mentioned mechanisms.^250^ Different cell types, either within the same tissue or between tissues, display strongly divergent methylome landscapes.^251,252^ This relates to different types and frequencies of mutations in tumors with different cell-of-origin.^253^ Strikingly, H3K9me3 alone can account for more than 40% of mutation rate variation in human cancer cells, and the numeral increases to 55% when combined with other types of histone methylation and chromatin organization features.^254^ Tumors with more DNA hypomethylation regions have higher frequencies of copy number variations, and regions suffering from differential methylation during cancer progression overlap with mutational hotspots.^255^

Mutually, genome instability/mutations lead to further disruption of the methylome. A comprehensive mutational cancer driver gene identifying pipeline called IntOGen integrates more than 28,000 tumors of 66 cancer types and identifies 568 driver genes, including 20 DNA and histone (de)methylation enzymes^253^ (Fig. 4a). Two genes (*MLL3* and *MLL4*) are the extremely wide drivers that drive more than 30 malignancies through mutations, with maximum mutation frequencies in skin cancers, i*.*e., 55% for *MLL4* in skin basal cell carcinoma (SBCC) and 37% for *MLL3* in skin squamous cell carcinoma (Fig. 4a). Nine genes including *NSD2*, *MLL2*, and *JARID1C* act as drivers in only one to three tumor types, and the rest nine genes including *DNMT3A*, *EZH2*, and *SET2* drive 5–18 malignancies (Fig. 4a). On the other hand, mutations at or near key sites of methylation can inhibit or ectopically enhance the modification activity. For example, H3K27M and H3K36M, two oncohistone mutations identified in 78% of diffuse intrinsic pontine gliomas and 95% of chondroblastomas, act as dominant-negative inhibitors of the H3K27 and H3K36 methyltransferases, leading to a global loss of H3K27 and H3K36 methylation, respectively.^256,257^ Mutations in DNA-binding motifs of CTCF and other regulatory factors significantly influence the methylation level of the CpGs in the neighboring regions, which is associated with cancer subtypes and patient survival.^258^ The deposition of m^6^A is regulated by cis-elements 50-nt downstream of the m^6^A sites, and mutations of these elements or the m^6^A site itself can influence the m^6^A deposition and the mRNA fate and subsequently the fitness of cancers.^259,260^ Therefore, the instability of genome and methylome across the central dogma and their interaction fuel tumor initiation and evolution.

#### Methylation and cancer initiation and progression

Recent sequencing studies indicate that cancer driver mutations are not rare in normal healthy tissues and can occur early in life.^261,262^ Only sporadic cells with these mutations transform into a malignant state. The oncogenic competence of a given mutation within the cells depends on the gene-expression programs which are associated with the microenvironment and the cell of origin and are shaped by methylation from DNA to proteins^263,264^ (Fig. 4b). For example, BRAF^V600E^ mutation readily transforms neural crest and melanoblast lineages but less so in the melanocytes in human pluripotent stem cell-derived cancer model and transgenic zebrafish model.^264^ Multiple DNA and histone (de)methylases (e.g., EZH2, TET1, and SET2) are highly expressed in these progenitor cells, and gain-of-function EZH2 mutations sensitize melanocytes to BRAF^V600E^-mediated transformation by global redistribution of H3K27me3, silence of ciliary genes, and activation of WNT/β-catenin signaling in mouse models.^264–266^ WNT pathway is also activated by age-associated DNA methylation remodeling that facilitates oncogenic BRAF mutations to drive colon cancer initiation in mouse colon-derived age-mimic organoid and aged animal models, which may be related to the higher risk of BRAF-mediated transformation from sessile serrated lesions in older individuals, despite these pre-cancerous polyps are equally represented across the age spectrum.^267,268^ Unlike DNA and protein methylation modifiers that frequently mutate during tumorigenesis (Fig. 4a), RNA methyltransferases and demethylases are frequently ectopic expressed in cancer tissues.^269^ The role of mRNA m^6^A modification in tumorigenesis is complex with both tumor-promoting and suppressing effects, depending on both specific sites and alteration of the m^6^A level (Fig. 4b). A panel of oncogenes (e.g., ADAM19, mTORC2, SP1) or TSGs (e.g., FOXM1, ASB2, LATS1) are targets of m^6^A pathway, which is co-opted by oncogenic mutations (e.g., *RAS*, *p53*, *MLL-fusion*) to initiate tumors.^270–274^ Furthermore, fluctuation in the m^6^A reading process can independently cause amplification of oncogenic signals while shrinkage in TSG signals (Fig. 4b). Alteration of methylomes also bridges exogenous carcinogens and transformation by driver mutations; for example, chronic cigarette smoke replaces H3K4me3/H3K27me3 bivalent histone marks with 5mC at promoters of a set of low-expression genes and primes human bronchial epithelial cells for cancer initiation by a single *KRAS* mutation.^275^ In addition to potentiating the oncogenic competence of driver mutations, methylation alteration plays a leading role in the initiation of some cancer types, which has been best demonstrated in some pediatric tumors with very few or no recurrent somatic mutations. For example, childhood ependymomas lack recurrent single nucleotide and focal copy number variations, instead displaying a switch between H3K27me3 and 5mC marks and response to inhibitors that target either DNMTs or PRC2/EZH2.^276,277^ Strikingly, *MLL1* (also known as *KMT2A*) genomic rearrangements that induce leukemia in an H3K79 methylation-dependent manner are sufficient to induce infant acute lymphoblastic leukemia in the background of fetal-specific gene expression programs.^278,279^ Although the crucial role of methylation reprogramming in cell transformation has been appreciated, unraveling the exact mechanism of how it drives cancer initiation remains a formidable challenge, due to the rarity and transient nature of these events. This is further aggravated by the complex reading systems of the methylation signals and the coexistence of driver and passenger methylation changes.^280^

After initiation, the tumor cells proliferate and progress toward aggressive cancers with increasing intratumoral heterogeneity (ITH) of cellular subpopulations that are associated with treatment resistance, metastasis, and relapse (Fig. 4b). ITH has traditionally been ascribed to genetic variation, recent studies indicate that epigenetic variation that is usually manifested as plastic DNA and histone methylomes is a major driver of phenotypic ITH with underlying transcriptomic heterogeneity. DNA methylation changes in human cancers are dominated by stochasticity and occur at different rates across the genome.^280^ An assay of CpG methylation changes in patient samples with chronic lymphocytic leukemia (CLL) showed that variation within DNA fragments (termed locally disordered methylation) rather than the variation between concordantly methylated fragments constitutes the basis of intratumor methylation heterogeneity.^281^ For instance, a fragment with 5 CpG sites has 2^5^ possible patterns or 32 epialleles. Such methylation heterogeneity contributes to transcriptional variation by regulating the activity of promoter, enhancer, or CTCF-mediated insulation, which may promote cancer evolution and causes adverse clinical outcomes.^281,282^ Although DNA methylation modifiers are rarely mutated in CLL, such mutations in individual samples (DNMT3A-Q153* and TET1-N789I) further increase the methylation heterogeneity, implying the difference in expression level, activity, or recruitment of methylation modifiers in the cancer cells causes the DNA methylation variation.^281^ Several histone demethylases, such as KDM5B (catalyzes H3K4me3 demethylation), are overexpressed in human tumors, which is associated with higher transcriptomic heterogeneity.^283^ Unlike locally disordered manner in DNA methylation, the difference in methylation peak broadness correlates with stochastic gene transcription.^284^ Specifically, higher KDM5B activity decreases H3K4me3 peak broadness, which results in rare events of active transcription, larger gene expression fluctuations, and elevated cellular transcriptomic heterogeneity. Conversely, broad H3K4me3 domains are associated with rapid activation of transcription with smaller fluctuations, contributing to high transcriptional consistency. Phenotypic ITH may be further enhanced by the m^6^A modification of mRNAs since the installation and downstream effects of m^6^A are heterogeneous across individual cells.^252,285^ The resource of m^6^A heterogeneity and the mechanism of how it contributes to ITH remain unknown, and the development of new detecting methods at the single cell level (e.g., scDART-seq) will help to resolve the fundamental questions.^252^ Tumor evolution can be driven by post-translational modification of nonhistone proteins, resulting in a non-stochastic probability of cancer cells with higher fitness.^286^ EZH2-catalyzed methylation of β-catenin enhances its stability by inhibiting ubiquitination-mediated degradation and activates Wnt–β-catenin signaling, which sustains self-renewal of cancer stem cells and may contribute to heterogeneity and recurrence of hepatocellular carcinoma^287^ (Fig. 4b).

#### Methylation and metastasis

Metastasis is the major cause of cancer-related death. Although large-scale prospective clinical sequencing found an association between genomic alterations and metastatic patterns, there are few metastasis-specific driver gene alterations compared to primary lesions.^288,289^ Metastasis involves multiple and even opposite steps including detachment and intravasation from primary sites and extravasation and colonization at distal sites, which requires a high degree of gene expression plasticity and reversibility that are readily achieved by non-genetic rather than genetic alterations, therefore, non-genetic variations, such as methylation changes at DNA, RNA, and protein levels, could be the main drivers of primary-to-metastasis transition. For example, dynamic changes of methylation level in *CDH1* (encodes a cell-cell adhesion glycoprotein E-cadherin) promoter contribute to the induction of epithelial-mesenchymal transition (EMT) in primary thyroid cancer and of the reverse process, mesenchymal-epithelial transition (MET), in lymph node metastases.^290^ Concerted histone and DNA hypomethylation, as a result of KMT2C deficiency and its link to *DNMT3A*, promotes metastasis of small cell lung cancer through activating metastasis-promoting *MEIS*/*HOX* genes.^247^ Inheritable metastasis-promoting gene expression signatures can be achieved by biotic and non-biotic factor-mediated methylome alteration, such as cell-cell contact and hypoxia which are unlikely to induce gene mutation. Specifically, in the circulatory system DNA methylation levels at binding sites of proliferation- and stemness-associated transcription factor genes, such as *OCT4*, *NANOG*, and *SOX2*, can be affected by the clustering state of circulating tumor cells, which is associated with the different metastatic capability of cluster and single circulating tumor cells of breast cancer patients and mouse models.^291^ Hypoxia promotes EMT and stem cell phenotypes through directly or indirectly suppressing oxygen-dependent H3K27me3 demethylases KDM6A/B, resulting in the persistence of H3K27me3, and subsequent silence of key genes (e.g., *DICER*)^292,293^ (Fig. 4b). Increasing evidence indicated that elevation in mRNA m^6^A level is involved in the EMT and cancer metastasis.^294,295^ Upregulation of METTL3 or downregulation of FTO promotes methylation of SNAIL or Wnt pathway transcripts, respectively, which enhances the translation or stability of target mRNAs that mediate the EMT process^294,295^ (Fig. 4b). Although RNA m^6^A modification itself is not considered to be inheritable, Genetic analyses supported the contribution of mRNA methylation to human disease heritability.^285^ The inheritance can be achieved by inheritable epigenetic changes in its writers (e.g., METTL3) and erasers (e.g., FTO) or alterations in H3K36me3 as it guides m^6^A deposition globally.^296^ Otherwise, a recent study showed RNA m^6^A modification regulates chromatin accessibility and gene transcription via recruitment DNA demethylase TET1 in normal and cancer cells,^297^ constituting a potential mechanism for the propagation of phenotypic changes during cell division. Beyond inheritance, plasticity, and reversibility, methylation change is a continuous variable that gives rise to continuous phenotypes of cancer cells, whereas genetic alterations usually produce discrete or binary phenotypic changes.^286,298^ Such continuous property greatly increases ITH within tumors and subsequent metastasis, which facilitates cancer cells to adapt to the dynamic environments during different stages of metastasis by achieving the fittest gene expression signatures.

In addition to methylation fluctuation, the activity of reader proteins influences the downstream events and promotes metastasis. For example, upregulation of the RNA m^6^A reader YTHDC1 facilitates TGFβ-mediated lung metastasis of triple-negative breast cancer through promoting nuclear export of SMAD3 transcripts and expression.^299^ Furthermore, YTHDF3 expression is specifically upregulated in brain metastasis but not in other metastases, which promotes the translation of brain metastasis-associated m^6^A-modified mRNAs including EGFR, GJA1, and ST6GALNAC5^300^ (Fig. 4b). These results suggest that different readout of methylation signals may correlate with organ-specific patterns of metastasis.

#### Methylation and tumor microenvironment

**Fig. 5:**
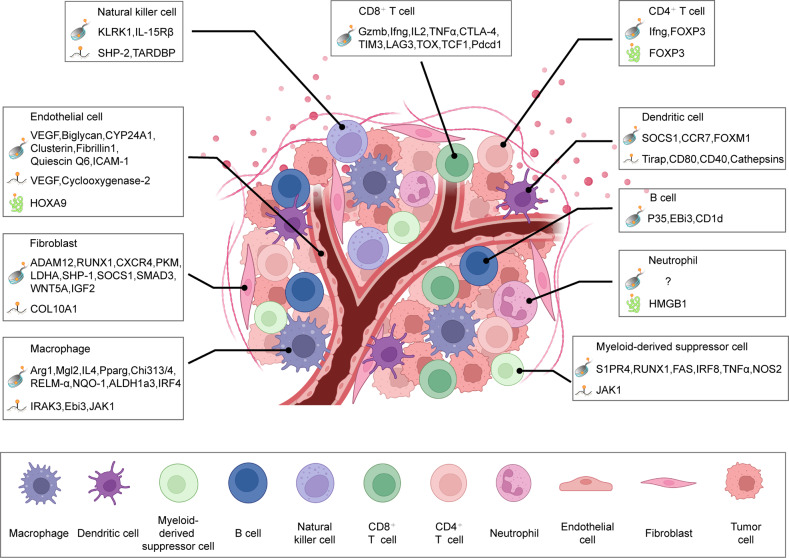
Methylation remodeling of non-tumor cells in the tumor microenvironment (TME). The TME is populated by various cell types including immune cells (e.g., T lymphocytes, natural killer cells, macrophages, neutrophils, and dendritic cells), stromal cells (e.g., fibroblasts and mesenchymal stromal cells), and blood and lymphatic vessels. To support tumor development and immune evasion, tumor cells induce alterations of the methylome landscape and subsequent gene expression changes in these TME cells. Examples of methylation remodeling of chromatin, mRNAs, and nonhistone proteins (represented by cartoons in the figure) are summarized

The initiation and progression of tumors are determined by both tumor cell-intrinsic properties and the surrounding tissue/organ environment. The tumor microenvironment (TME) is typically composed of immune cells (e.g., T lymphocytes, natural killer cells, macrophages, neutrophils, dendritic cells), stromal cells (e.g., fibroblasts and mesenchymal stromal cells), blood and lymphatic vessels, extracellular matrix (e.g., collagens, fibronectins, and elastin) and other secreted molecules by tumor and non-tumor cells (e.g., cytokines, growth factors, and chemokines) (Fig. 5). These TME components that usually suppress tumor in their naïve states are reshaped by tumor cells to support growth, angiogenesis, immune evasion, local invasion, metastasis, and therapy resistance of tumors, which involves gene expression reprogramming in non-tumor cells. Although a recent study observed increased somatic copy number alterations in tumor-associated fibroblasts of patients with colorectal cancer,^301^ generally these non-tumor cells are genetically stable and their tumor-supporting gene expression programs are established through non-genetic mechanisms, including methylation of DNA, RNAs, and proteins.^302,303^ Through direct cell-cell contact, secreted molecules, or tumor-induced extracellular physicochemical changes (e.g., hypoxia, elevated extracellular potassium), tumor cells induce alteration of methylome landscape and subsequent gene expression changes in TME cells, which can be fixed to robustly facilitate tumor progression due to the inheritability of chromatin methylation.^304–307^ In the following discussion, we focus on how methylation remodeling in the various TME cells regulates tumor development and progression (Fig. 5).

##### T cells

The immune landscape within TME can be classified into three main categories based on the spatial distribution of T cells: (1) immune-inflamed landscape in which T cells infiltrate and distribute throughout the tumor; (2) immune-excluded landscape in which T cells accumulate at the periphery of tumor; (3) immune-desert landscape in which T cells are completely lacking or at very low numbers.^308^ The expression of DNA/RNA methylation regulators or methylation profiling of tumors can be used to identify the immune landscape type of individual tumors with mathematical tools, which is consistent with the important roles of methylation in the regulation of tumor immune landscape.^309–311^ There are two major classes of T cells: CD4^+^ and CD8^+^ T cells. CD8^+^ T cells, also known as cytotoxic T cells, recognize antigens presented by MHC-I molecules on the tumor cell surface and kill the tumor cells. Accordingly, tumor cells downregulate the expression of MHC-I molecules or neoantigens to evade immune surveillance of the T cells through promoter hypermethylation of the encoding genes.^312,313^ Differentiation and activation of naïve CD8^+^ T cells, featured by the induction of a transcriptional program that facilitates rapid expansion, migrating into tumor tissue, and expression of key cytokines (e.g., IFNγ and IL-2) and effector proteins (e.g., granzymes and perforins), is associated with global DNA and histone methylome remodeling.^314,315^ For example, the DNA 5mC signal is erased from the promoters of *Gzmb* (encoding granzyme), *Ifng* (encoding IFNγ), and the inhibitory receptor *Pdcd1* genes to derepress their expression in the activated CD8^+^ T cells. In the setting of acute infections, most activated CD8^+^ T cells die and a small portion survive and differentiate into memory CD8^+^ T cells after clearance of infectious agents, with much of the activated effector genes being silenced again by DNA methylation in the memory CD8^+^ T cells. These memory CD8^+^ T cells can provide long-term immunity against pathogens and cancers, as they can rapidly replenish effector CD8^+^ T cells upon restimulation.^316^ However, in the setting of cancer, chronic stimulation by antigens and/or inflammation causes their differentiation into a dysfunctional or exhausted phenotype, resulting in failure of cancer eradication by T cells in many patients. The differentiation to exhaustion phenotype is progressive, consistent with progressive alterations of the underlying transcriptome and epigenome.^317^ Terminally exhausted CD8^+^ T cells show poor proliferative and cytolytic capacity, low expression of effector cytokines (e.g., IFNγ, IL-2, and TNFα), and sustained upregulation of multiple inhibitory receptors (e.g., PD-1, CTLA-4, TIM3, and LAG3), which is associated with aberrant DNA and histone methylation, i.e., suppression of effector-related genes by DNA methylation, repressive histone methylation or histone bivalency (H3K4me3/H3K27me3), and activation of inhibitory genes by DNA demethylation and activating histone modification.^305,318,319^ Epigenetic stability of exhausted CD8^+^T cells prevents function recovery by simple immune-checkpoint blockade therapy.^318,320^ Not only endogenous CD8^+^ T cells, but also engineered adoptive CD8^+^ T cells undergo exhaustion-associated DNA methylation reprogramming that dampens chimeric antigen receptor (CAR) T-cell therapy, characterized by the methylation and silence of genes associated with memory potential (e.g., *TCF7* and *LEF1*) and demethylation and activation of exhaustion-driver genes (e*.*g., *TOX*, *CX3CR1*, and *BATF*).^321–323^ Therefore, understanding the mechanisms of T cell-exhaustion induction is crucial yet challenging for cancer prevention and immunotherapy.

Persistent tumor antigen exposure, inhibitory receptors/ligands, suppressive cytokines, and harsh TME (e.g., hypoxia and nutrient levels) contribute to CD8^+^ T cell exhaustion.^324^ Methylome remodeling is involved in the integration of these signals within the T cells, as well as the upregulation of these signals in tumor and TME cells. Tumor antigen overstimulation is doubtlessly a crucial inducer for CD8^+^ T cell exhaustion, and multiple transcription factors or regulators are identified, such as HMG-box transcription factor TOX, T cell factor-1, and death-associated protein like 1 (Dapl1).^325–327^ However, the details of how they respond to upstream antigen overstimulation and orchestrate downstream methylome remodeling remain largely unknown. While Yin Yang-1 (YY1) transcription factor recruits EZH2 histone methyltransferase to the *IL-2* locus and inhibits the production of the pivotal cytokine in an in vitro exhaustion model created by persistent antigen and co-stimulatory signal stimulation, whether YY1 regulates T cell exhaustion in vivo in a similar way remains to be investigated.^328^ Inhibitory receptors and their ligands are ectopically upregulated in tumor-infiltrating CD8^+^ T cells and tumor cells respectively, marking T cell exhaustion and immunosuppression. DNA hypomethylation, loss of repressive histone methylation (e.g., H3K9me3, H3K27me3), and/or mRNA methylation contribute to their upregulation in tumor tissue.^319^ It should be noted that the effect of mRNA methylation is dependent on the reader proteins in different cancer types, e.g., YTHDF2 promotes degradation of m^6^A-modified PD-L1 mRNA in intrahepatic cholangiocarcinoma while IGF2BP3 enhances the stability of the modified PD-L1mRNA in breast cancer, resulting in an opposite influence on T cell exhaustion and tumor immune escape.^329,330^ Suppressive cytokines, including IL-10 and TGFβ that are secreted by tumor cells and TME cells (including immune cells and stromal cells), promote T cell exhaustion and tend to be overproduced in different cancer types by epigenetic alterations. For example, both promoter and gene-body hypomethylation of *IL-10* contribute to the upregulation of IL-10 by enabling access to activating transcription factors, such as STAT3 and Sp1.^331,332^ The development and differentiation of T cells require adequate oxygen and nutrient supplies which are undermined in TME by tumor cells. For nutrient supply, a prominent example is the tug-of-war between cancer and T cells for methionine uptake, an amino required for the synthesis of the common methyl donor SAM for DNA, RNA, and protein methylation in T cells.^333^ Cancer cells express high level of the methionine transporter SLC43A2 and outcompete for methionine, leading to lower intracellular level of SAM and loss of histone H3K79 methylation in CD8^+^ T cell, which in turn transcriptionally downregulates the expression of STAT5 transcription factor and causes T cell death and dysfunction.^333^ Exposure to hypoxia in TME directly inhibits the activity of oxygen-sensitive histone demethylases including KDM5A (H3K4me3 demethylase) and KDM6A (H3K27me3 demethylase), leading to increased histone bivalency and reduced transcription of genes required for robust effector response in terminally exhausted CD8^+^ T cells.^305^ Notably, the bivalent gene set was not significantly changed in the chronic viral infection-inducted exhaustion, suggesting bivalency is specific to TME-driven exhaustion of T cells.^305^

CD4^+^ T cells recognize antigens presented by MHC-II molecules that are mainly expressed in professional antigen-presenting cells (APCs), such as dendritic cells, B cells, and macrophages. Depending on their patterns of cytokine and function, CD4^+^ T cells are classified into multiple subtypes including T-helper 1(Th1), Th2, Th17, and regulatory T cells (Treg). These subtypes extensively regulate immune responses within TME, resulting in either tumor progression or regression. Th1 cells are characterized by the production of IL-2 and IFNγ, which suppresses tumor progression at least by supporting CD8^+^ T cell function, facilitating recruitment of antigen-presenting cells, and inhibiting tumor angiogenesis. In contrast, Treg cells, characterized by expression of transcription factor forkhead box P3 (FOXP3) and the high-affinity heterotrimeric IL-2 receptor (CD25), generally inhibit antitumor immunity through secreting immunosuppressive cytokines (e.g., TGFβ, IL-10, and IL-35), sequestrating IL-2, producing immunomodulatory metabolite (adenosine), expressing inhibitory receptors (e.g., CTLA-4 and LAG-3).^334^ The roles of Th2 and Th17 cells in tumor progression are ambivalent, depending on the tumor phenotypes.^335^ The differentiation and function of the different subtypes depend on a particular cytokine milieu upon activation by antigens, followed by the expression of master regulators and methylome remodeling.^336,337^ For example, deposition of activating histone mark H3K4me3 is associated with the expression of signature-cytokine genes (e.g., *Ifng*, *Il4*, and *Il17*) in their corresponding CD4^+^ T cell lineages (Th1, Th2, and Th17); active DNA demethylation at the Treg-specific demethylated region within the *FOXP3* locus and maintenance of global DNA methylation is required for Treg differentiation and stability of suppressive function;^338,339^ METTL3-meditated mRNA methylation is implicated in the control of homeostatic proliferation and differentiation of CD4^+^ T cells and the maintenance of Treg suppressive functions by targeting IL-7/STAT5/SOCS and IL-2/STAT5/SOCS pathways, respectively.^340,341^ In addition, methylome remodeling mediates transdifferentiation between CD4^+^ T subtypes. The poised chromatin state, featured by the presence of histone bivalency and lack of DNA methylation, at lineage-specific loci in nonexpressing lineages contributes to this cellular plasticity. For example, *TBX21* (a master regulator gene of Th1 differentiation) is marked by histone bivalency in nonexpressing lineages and involved in KDM6B(an H3K27me3 demethylase)-mediated transdifferentiation of Th2, Th17, and Treg cells to Th1 cells.^342,343^

Given the importance of the methylation program in the lineage commitment of CD4^+^ T cells, tumors often induce immunosuppression through methylation remodeling of key effector genes or lineage-specific master regulator genes, skewing toward protumor Treg rather than antitumor Th1 phenotype. *Ifng* promoter region is significantly more methylated in CD4^+^ T cells of TME than that of tumor-draining lymph nodes from patients with colon cancer, showing Th1 lineage restriction of tumor-infiltrating lymphocytes.^344^ Treg cells are epigenetically reprogrammed in the TME for complete activation with the help of several important transcription factors, such as BATF and IRF4.^345–347^ After activation, EZH2-mediated histone H3K27 methylation is involved in the maintenance of FOXP3 expression and Treg identity in non-lymphoid tissues, which contributes to cancer immune tolerance.^348,349^ Moreover, PRMT1- or PRMT5-mediated arginine methylation of FOXP3 is crucial for its transcriptional activity and the function of Treg, and interference with such arginine methylation improves antibody-mediated targeted therapy in a mouse model by inhibiting Treg function and inducing tumor immunity.^350,351^ Therefore, through these methylation reprogramming mechanisms Treg cells accumulate in TME and compromise antitumor immunity of infiltrating CD8^+^ T cells, contributing to tumor development and progression.

##### B cells

Like the T cells, B cells are heterogenous and contain multiple subsets, including antibody-secreting plasma cells and regulatory B (Breg) cells. They play different roles in the tumor immune response, resulting in either tumor regression or progression, depending on the phenotype of both tumor and B cells. The antitumor effects of B cells are achieved by producing antibodies that can mediate antibody-dependent cell cytotoxicity and phagocytosis of cancer cells by natural killer cells and macrophages respectively, presenting antigen to T cells, secreting proinflammatory cytokines (IFNγ and IL-12) that promote cytotoxic immune responses, and directly killing cancer cells with granzyme B and death ligand TRAIL.^352^ Such antitumor effects of B cells are augmented when they form tertiary lymphoid structures (TLSs) with other immune cells including T cells and dendritic cells.^353,354^ Consequently, the presence of B-cell-rich TLSs is often correlated with a positive prognosis and a strong indicator of better survival upon immune-checkpoint blockade treatment in various tumor types, such as sarcoma, melanoma, renal cell carcinoma, and lung cancer.^355–358^ Moreover, de novo induction of TLS in tumors improves immunotherapy in a mouse model of resistant insulinomas.^359^ Alternatively, B cells in patients often fail to perform an antitumor function but promote tumorigenesis through secreting immunosuppressive cytokines (e.g., IL-10, IL-35, and TGFβ) and small metabolite (GABA) and expressing inhibitory ligand (PD-L1), leading to inhibition of cytotoxic CD8^+^ T cells and natural killer cells.^352,360^ Immunosuppressive and tumor-promoting B cells are generally attributed to the presence of Breg subsets. A recent study showed that IL-35 is involved in Breg differentiation, implying a positive feedback loop exists to consolidate the immunosuppressive role of B cells in tumors.^361^ The balance between antitumor B cell lineages and Breg cells determines the immune response and is affected by cancers. Pancreatic cancer shifts the B cell differentiation away from plasma phenotype and toward Breg phenotype through IL-35/STAT3/PAX5 pathway, which supports tumor growth, suppresses CD8^+^ T cell-mediated immunity, and dampens anti-PD1 immunotherapy.^361–363^ Upregulation of several lysine demethylases (e.g., KDM6A/B) and removal of repressive H3K27me3 histone marks at downstream target genes contribute to this Breg differentiation.^361^ How methylation reprogramming of DNA, RNAs, and proteins orchestrates B-cell activation and differentiation in the peripheral lymphoid system is relatively well documented,^364^ while counterpart information in tumors is much less. Compared with T cells, B cell infiltration in TME is relatively few. However, as the importance of B cells in tumor development and treatment is emerging, especially recent pan-cancer studies showed that the presence of tumor-infiltrating B cells and/or activated natural killer cells is correlated with exceptional responses to treatment,^365,366^ it is expected that the gap will be filled soon in future.

##### Natural killer cells

Natural killer (NK) cells belong to the innate lymphoid cell family and possess remarkable cytotoxic potential. They constitute the first line of immunity against tumor initiation and progression by directly killing tumor cells or enhancing both innate and adaptive immune responses. The former is achieved by releasing granzymes and perforin or expressing death ligands (e.g., FasL and TRAIL), and the latter is mediated by secreting pro-inflammatory cytokines (e.g., IFNγ).^367^ NK cells do not express somatically rearranged T or B cell antigen receptors to specifically pick up tumor cells, instead, they target tumor cells with MHC class I-deficiency (missing-self signal) and/or stress-induced (e.g., DNA damage) activating ligand overexpression. The two signals correspond to inhibitory and activating receptors on the NK cell surface, the balance of which regulates whether NK cells initiate attack. Inhibitory receptors including the killer cell immunoglobulin (Ig)‑like receptors (KIRs) bind MHC-I molecules to protect healthy cells from NK cell attack, while tumor cells downregulate MHC-I expression in an attempt to evade CD8^+^ T cell response and activate NK cell response. Alternatively, the inhibitory signals are overridden by upregulated activating ligands in tumor cells with the full expression of MHC-I.^368^ Suppressing ligand-receptor interactions between tumor and NK cells constitutes an important mechanism to escape detection and eradication by NK cells, which is underpinned by methylation reprogramming. For instance, IDH-mutation mediated DNA hypermethylation and EZH2-catalyzed H3K27 methylation transcriptionally suppress the expression of NK group 2D (NKG2D) ligands (e.g., ULBP1 and ULBP3) in glioma and hepatocellular carcinoma, respectively.^369,370^ Furthermore, circular EZH2 RNA derived from circularization of exon-2 and -3 of the *EZH2* gene encodes a functional shortened version protein called EZH2-92aa which represses the transcription of multiple NKG2D ligands both directly (by binding to the gene promoters) and indirectly (by stabilizing EZH2).^371^

NK cells show distinct DNA methylation and hydroxymethylation landscapes among innate lymphoid cell lineages,^372^ and their differentiation and activation are regulated epigenetically and epitranscriptionally.^373,374^ EZH2-catalyzed H3K27 methylation negatively regulates the differentiation and function of NK cells through downregulating NKG2D receptor and IL-15 receptor β chain (IL-15Rβ/CD122), a cytokine pathway crucial for the survival, proliferation, and activation of NK cells.^375^ Therefore, inhibition of EZH2 has the potential to kill two birds with one stone: upregulation of NKG2D ligands in tumor cells to expose them to NK cells and increasing NKG2D receptor and IL-15Rβ in NK cells to promote effector function, which ultimately mediates NK cell-based tumor eradication. METTL3-catalyzed m^6^A modification of *SHP-2* mRNAs is required for SHP-2 expression and response to IL-15, whereas the expression of METTL3 is reduced in tumor-infiltrating NK cells from patients with hepatocellular carcinoma, compromising tumor immune-surveillance function of NK cells.^374^ The m^6^A reader YTHDF2 that works downstream of the IL-15 signaling pathway is required for the antitumor activity of NK cells by forming a STAT5-YTHDF2 positive feedback loop and targeting *TARDBP* mRNAs.^376^ Like CD8^+^ T cell exhaustion, chronic exposure to activating stimuli during tumorigenesis can cause NK cell exhaustion, accompanied by global DNA methylation changes.^377,378^ Other mechanisms contributing to tumor evasion of NK cells include expressing immune checkpoint ligands (PD-L1, LAG3, and TIM-3), releasing immunomodulatory molecules (IL-10, TGFβ, and prostaglandin E_2_), and expressing inhibitory NK cell ligands (HLA-E and HLA-G).^379^ However, whether and how methylation changes within NK cells affect antitumor activity upon these inhibitory interactions conducted by cancer cells remain unknown, and filling up the gap would help to develop more effective NK cell-based immunotherapy.

##### Macrophages

Macrophages are derived from myeloid cells in the bone marrow and abundantly populate the majority of solid tumors. They exhibit a high degree of phenotypic and functional plasticity, with pro-inflammatory (M1) and anti-inflammatory (M2) states representing the two ends of the phenotypic spectrum of macrophages.^380^ M1 macrophages coordinate innate and adaptive immune responses and restrain tumor development through phagocytosis, antigen presentation, and secretion of pro-inflammatory cytokines (e.g., TNFα and IL-6); while M2 macrophages that normally function in wound healing and tissue repair promote tumor progression and metastasis by secreting immunosuppressive factors (e.g., IL-10), pro-angiogenic cytokines (e.g., VEGF), growth factors (e.g., EGF), and extracellular matrix remodeling proteases (e.g., matrix metallopeptidases).^381^ The phenotypes and the underlying epigenetic identity and gene expression signature of macrophages are ready to be sculpted by the niche environments, which provides a rationale for the education of macrophages in the TME towards M2 state through hypoxia, tumor-derived metabolites (e.g., lactic acid), and secreted cytokines (e.g., IL-4).^382–384^ As a result, high macrophage infiltration often correlates with poor patient prognosis.

Multiple modifiers of DNA/RNA/protein methylation are implicated in regulating the balance between immunosuppressive and proinflammatory polarization of TAMs, and controversial results were reported for certain of them. IL-1R-MyD88 axis-induced TET2 expression in tumor-associated macrophages (TAMs) maintains the expression of immunosuppressive genes (*Arg1, Mgl2, and Il4*) and M2 macrophage function, which inhibits the antitumor T cell response and increases melanoma tumor burden.^385^ DNMT3B promotes macrophage polarization to classically activated M1 phenotype in murine adipose tissue macrophages and RAW264.7 macrophage cells by promoter methylation and silence of peroxisome proliferator activated receptor γ (*Pparg*) gene which encodes a key transcription factor promoting M2 polarization.^386^ Consistently, DNMT inhibitor (DNMTi) treatment in a mouse model of pancreatic ductal adenocarcinoma (PDAC) increases M2 polarization of tumor-infiltrating macrophages that express markers including Arg1, Chi3l3/4, and the resistin-like molecule (RELM)-alpha/FIZZ1.^387^ In contrast, GARP- and integrin αV/β8-mediated interaction between PDAC tumor cells and M1-like macrophages induces metabolic and phenotypic reprogramming to M2-like macrophages in a DNMT-dependent manner, in which some glucose metabolism and OXPHOS genes are methylated and downregulated.^388^ DNMTi treatment increases M1 macrophages while reducing M2 macrophages in the TME of murine models of ovarian cancer, through activating type I IFN signaling.^389,390^ The different roles of DNA methylation writers and erasers may be associated with tumor types, the specific TME, and upstream signaling pathways. Things are more complex for the modifiers of protein methylation in the regulation of macrophage polarization. Enzymatic activity-dependent and -independent mechanisms and opposite roles of the same modifier or the modifiers that catalyze the same methylation reaction are observed. For example, KDM6B facilitates macrophage M1 polarization through H3K27 demethylation-dependent and independent pathways and serves as a target of cancer-derived exosomal miR-138-5p which promotes the progression of breast cancer;^391,392^ while acting downstream of PERK (encoded by *EIF2AK3*) signaling, α-KG synthesis and KDM6B-catalyzed H3K27 demethylation at M2 genes (*IRF4*, *MGL2*, and *PPARG*) reinforces immunosuppressive activity of TME-educated macrophages.^393^ SETD4 positively regulates TLR-induced IL-6 and TNFα production in LPS-stimulated macrophages by directly methylating histone H3K4 of their promoters,^394^ while another H3K4 methyltransferase Ash1l negatively modulates this process by methylation and induction of the ubiquitin-editing enzyme A20 that mediates deubiquitination of NF-κB signal modulator NEMO and transducer TRAF6,^395^ indicating different target choices contribute to the divergent influences of these protein (de)methyltransferases on macrophage polarization. However, major information about the function of protein (de)methyltransferases in macrophage polarization, including SETD4, Ash1l, PRMT1, and G9a, is obtained using classical inflammatory macrophage models, and therefore the further study of TAM models is needed to get a precise conclusion of their roles in the tumor progression.^396^ The role of RNA m^6^A methylation in TAM polarization is emerging and also under debate.^397^ Initially, METTL3/METTL14-YTHDF1-3 axes were shown to drive the polarization bias of TAMs toward M1 phenotype, promote CD8^+^ T cell function, and inhibit colon cancer progression by targeting mRNAs of immunosuppressive factors for degradation, such as IL-1 receptor-associated kinase 3 (IRAK3, also known as IRAKM) and Epstein-Barr virus-induced protein 3 (Ebi3).^398,399^ In contrast, a recent study in colon cancer displayed that the METTL3-m^6^A-YTHDF1 axis fosters immunosuppression of tumor-infiltrating myeloid cells including M2 macrophages, through enhancing the translation of m^6^A-modified *Jak1* mRNA.^400^ Furthermore, this study showed that lactylation modification of METTL3 is crucial for its target RNA capture. These results imply a sophisticated regulatory network of RNA methylation pathway that may involve post-translational modification of writers, target choice, and readout manner for modulating the macrophage phenotypes in the TME, which requires further exploration and clarification.

##### Myeloid-derived suppressor cells

Myeloid-derived suppressor cells (MDSCs) are heterozygous populations of bone marrow-derived immature myeloid cells with an immunosuppressive function in TME. Phenotypically, MDSCs are characterized by the expression of myeloid markers (CD33 and CD11b) but lack MHC II (HLA-DR) expression, and they are generally classified into two main subgroups according to their morphological resemblance to monocytes and neutrophils, i.e., monocytic MDSCs (M-MDSCs) and polymorphonuclear MDSCs (PMN-MDSCs) (also known as granulocyte-MDSCs (G-MDSCs). Multiple mechanisms are used by MDSCs to dampen cytotoxic T cells and NK cells, such as the production of nitric oxide (NO) and reactive oxygen species (ROS), secretion of immunosuppressive cytokines (IL-10 and TGFβ), expression of ARG1, indoleamine 2, 3-dioxygenase, and immune checkpoint molecules (PD-L1 and CTLA-4), differentiation into suppressive TAMs (mostly from M-MDSCs).^401,402^ The differentiation, activation, and survival of MDSCs are regulated by tumor-derived factors (e.g., GM-CSF, IL- 6, and IL-1β), followed by methylation remodeling of DNA/RNA/protein.

Distinct DNA methylation patterns are observed between antigen-presenting myeloid cells (macrophages and dendritic cells) and myeloid suppressive cells under normal physiological conditions and within different MDSC subsets in TME.^403,404^ Immunosuppressive molecules including TGFβ1, TIM-3, and ARG1 are highly expressed with their promoters highly demethylated in CD33^+^HLA-DR^–^ cells compared with antigen-presenting myeloid cells,^404^ and genes related to DNA methylation are deregulated in tumor-infiltrating MDSCs.^403^ Tumor-derived factors, such as prostaglandin E2, shift differentiation from immune-promoting dendritic cells to MDSCs by upregulating DNMT3A and consequently methylating and inhibiting immunogenic-associated genes (e.g., *S1PR4*, *RUNX1*, and *FAS*).^405^ In addition to specific gains of DNA methylation during this differentiation, extensive demethylation also occurs, consistent with the fact that TET2 expression is upregulated and contributes to the immunosuppressive function of MDSCs during melanoma progression.^385^ IFN regulatory factor 8 (IRF8) is an important transcription factor for the differentiation and apoptosis of myeloid cells, and IRF8 deficiency facilitates MDSC differentiation and correlates with increased MDSC levels in cancer patients.^406,407^ Although *IRF8* promoter is hypermethylated in tumor-induced MDSCs, DNA methylation regulates MDSC accumulation via an IRF8-independent mechanism in which IL6-STAT3-DNMT axis silences TNFα expression and suppresses downstream RIP1-dependent necroptosis to support MDSC survival.^408^ Similarly, SETD1B bypasses a normal role for IRF8 in stimulating iNOS expression in MDSCs and instead directly deposits H3K4me3 signals at the *NOS2* promoter to activate the gene expression in tumor-induced MDSCs.^409^ Another H3K4 methyltransferase, MLL1, negatively regulates the expansion, activation, and differentiation of PMN-MDSCs, and tumor-secreted factors, as well as GM-CSF + IL-6, activate STAT3/CEBPβ to induce microRNAs targeting MLL1-complex (containing WDR5 and ASH2L) during accumulation and activation of PMN-MDSCs.^410^ Although the role of H3K27 acetylation in the regulation of MDSC-associated genes expression (e.g., *NOS2*, *Arg1*) in tumors was reported,^411^ whether and how methylation of H3K27, as well as other modification sites of histones, modulate the fate of tumor-infiltrating MDSCs need further investigation. Like the function of the METTL3-m^6^A-YTHDF1 axis in TAMs mentioned above, this axis strengthens immunosuppressive functions (e.g., generation of NO and ROS) of tumor-infiltrating MDSCs by upregulating JAK1-STAT3 signaling pathway.^400^ Moreover, intratumoral MDSC expansion is associated with high levels of METTL3 in tumor tissues of patients with cervical cancer, and the induction of CD33^+^CD11b^+^HLA-DR^−^ MDSCs and tumor-derived MDSCs in vitro is weakened in METTL3-deficient CD33^+^ cells.^412^ The potential role of the RNA methylation pathway in regulating MDSC differentiation and homeostasis requires further confirmation and study in vivo, as well as the exact mechanism and downstream targets.

##### Dendritic cells

Dendritic cells (DCs) are heterogeneous and comprise four main subtypes: conventional DC 1 (cDC1), conventional DC 2 (cDC2), plasmacytoid DC (pDC), and monocyte-derived DC (moDC).^413^ DCs not only are professional APCs that present antigens on MHC II molecules to prime CD4^+^ T cells but also are the main APCs that crosspresent tumor-associated antigens on MHC I molecules to activate CD8^+^ T cells, which is essential for antitumor immunity. The activation and maturation of DCs are stimulated by damage-associated molecular patterns of necrotic cells and/or type I interferons within TME. On the other hand, DCs are implicated in the induction of central and peripheral immune tolerance, and they achieve tolerogenic/immunosuppressive functions under the stimulation of various factors, such as IL-10, TGFβ, and vitamin D3.^414^ This is often co-opted by tumors to shift the phenotypes of infiltrating DCs from immunogenic to tolerogenic, which dampens antitumor immunity and promotes tumor progression.^415^ The maturation and functional plasticity of DCs in TME involves methylation remodeling across DNA/RNA/protein and related writers, erasers, and readers.

Different DNA methylation levels and patterns are needed for the development of DC subsets, with pDC showing the highest DNA methylation levels across differentially methylated regions (DMRs) and most sensitive to hypomethylating perturbation.^416,417^ DNMT1 deficiency leads to hypomethylation and upregulation of suppressor of cytokine signaling (SOCS)1 and impairs the maturation of tumor-associated DCs.^418^ Meanwhile, TET2-mediated DNA demethylation along with the JAK3-STAT6 pathway is crucial for moDC differentiation upon IL-4 and GM-CSF stimulation, and elevating the enzymatic activity of TET2 by vitamin C treatment during the differentiation improves the immunogenic properties of moDCs, supporting the proliferation and function of T cells and the cancer immunotherapy.^419–422^ In contrast, TET2 along with the JAK2-STAT3 pathway is indispensable for the vitamin D-mediated establishment of tolerogenic/immunosuppressive moDCs.^423^ Similarly, histone methylation (H3K4me3 and H3K27me3) regulates the differentiation of moDCs into either immunogenic or tolerogenic phenotypes, depending on the environmental stimuli.^424^ Tumor-derived TGFβ induces alteration in H3K4me3 and H3K27me3 landscapes and represses the maturation and function of DCs, which involves downregulation of MHC class II, costimulatory molecules, the chemokine receptor CCR7, and type I IFN.^425–427^ CCR7 is a key determinant for the migration of mature DCs (primarily cDC1s) to the lymph nodes where T cell priming occurs, and distinct levels of H3K27me3 account for different migration abilities of DC subsets.^413,428^ Besides H3K4 and H3K27 methylation, DOT1L-catalyzed H3K79 methylation at the *Forkhead box transcription factor M1* (*FOXM1*) promoter enhances FOXM1 expression that inhibits the DC maturation via the Wnt5a signaling pathway, which is associated with the poor survival in pancreatic cancer and colon cancer patients with high DC infiltration.^429^ The m^6^A-YTHDF1 axis promotes DC activation and function to augment CD4^+^ T cell proliferation by enhancing the translation of some immune-related transcripts, including Tirap, CD80, and CD40 mRNAs.^430^ However, this axis negatively regulates the antigen crosspresentation ability of cDCs by enhancing the translation of targeted lysosomal protease transcripts, which restrains the priming of CD8^+^ T cells and antitumor immunity.^431^ The underlying mechanism for the distinct roles of m^6^A in DC function modulation remains unknown. Nonetheless, these studies indicate a flexible role of RNA methylation, as well as DNA and protein methylation, in DC-mediated antitumor immunity.

##### Neutrophils

Neutrophils constitute 50–70% of circulating leukocytes in humans and are essential components of innate immunity against invading pathogens. Like macrophages, tumor-associated neutrophils polarize to either antitumor (N1) or protumor (N2) phenotype. The N1 neutrophils are short-lived and use tools of anti-infection to eliminate tumor cells, including performing antibody-dependent cell-mediated cytotoxicity (ADCC), releasing chromatin DNA filaments (known as neutrophil extracellular traps (NETs)), and generating superoxide (H_2_O_2_ and HOCl), and stimulating adaptive antitumor immune responses. In contrast, the N2 neutrophils are long-lived, low cytotoxic, and positively involved in tumor initiation, growth, metastasis, angiogenesis, and immune evasion.^432^ Notably, neutrophils participate in the entire metastatic process from primary tumors to the distant organs, which involves distinct subsets of neutrophils: MMP-9^+^TIMP^-^ neutrophils promote angiogenesis and intravasation through releasing MMP-9 at the primary tumor sites;^433^ neutrophils cluster with circulating cancer cells (CTCs) to promote cell cycle progression within the bloodstream;^434^ NET DNA acts as both chemotactic factor and trap to facilitate CTC landing at distant organs;^435,436^ a subset of neutrophils featured by P2RX1 deficiency foster immunosuppressive metastatic TME through upregulating immune exhaustion-related genes (e.g., *PD-L1*).^437^ The protumor effect of neutrophils dominates in patients, and consequently, neutrophil-based assays, including the number of tumor-associated neutrophils, circulating neutrophil level, neutrophil to lymphocyte ratio, and CD8^+^ T cell to neutrophil ratio, are developed to predict patient prognosis and responsive to treatments.^438^ The phenotypes of neutrophils are shaped by tumor cells and other cells within TME through released cytokines, such as TGFβ, IL-6, and IFNβ. TGFβ^439^ and IL-6^440^ drive N2 phenotype, while IFNβ^441^ stimulates N1 phenotype. Furthermore, in tumor-bearing mice TGFβ can reverse mature antitumor neutrophils to pro-tumor immunosuppressive counterparts that accumulate in cancer patients, indicating phenotypic plasticity of mature neutrophils.^442^

Although DNA/RNA/protein methylation remodeling in tumor cells for motivating, recruiting, and polarization of neutrophils has been studied,^443,444^ the methylation mechanism for intrinsically regulating neutrophils is still largely unknown, due to technical difficulties in isolating tumor-associated neutrophils from human samples at high purity. Dynamic DNA methylation remodeling is involved in the neutrophil differentiation from the multipotent common myeloid progenitors,^445^ and dysregulation in DNA methylation contributes to neutrophil-based autoimmune vasculitis,^446^ suggesting the role of DNA methylation in modulating neutrophil fate and function, which is pertinent to diseases. Tumor-infiltrating neutrophils form a positive feedback loop with glioblastoma cells to facilitate further neutrophil infiltration and tumor progression through releasing High Mobility Group Box 1(HMGB1) and activating the NF-κB signaling pathway in glioblastoma.^447^ The exposure of nuclear-localized HMGB1 is a result of NET formation, and mono-methylation of HMGB1 at Lys42 changes the protein conformation and causes its cytoplasmic localization.^448^ The oncoprotein growth factor independent 1 (GFI-1) is essential for neutrophil differentiation and maturation after the myeloblast stage and acts as a transcriptional repressor that cooperates with suppressive histone modifiers, such as G9a (H3K9 methylation) and LSD1 (H3K4 demethylation).^449^ These results imply a potential role of histone and nonhistone protein methylation in regulating neutrophil recruitment and function in tumors. A recent study demonstrated the migration of neutrophils to infectious sites is empowered by the m^6^A demethylase ALKBH5 that changes the degradation of migration-related transcripts (*CXCR2*, *NLRP12*, *PTGER4*, *WNK1*, and *TNC*).^450^ Up-regulation of CXCR2 is also crucial for neutrophil recruitment in tumors,^438^ suggesting a potential role of neutrophil RNA methylation in regulating tumor progression.

##### Endothelial cells

When tumors reach about 2 mm^3^ in volume, passive diffusion cannot meet the further demand for oxygen and nutrients, and tumors have to establish their blood supply system.^306^ Endothelial cells (ECs) form tumor blood vessels to deliver nutrients and other essentials for tumor growth. In addition, tumor blood vessels enable the dissemination of tumor cells to distant organs. Stimulated by excessive proangiogenic factors (e.g., VEGF, PDGF, and EGF), the new tumor vessels are malformed and dysfunctional, characterized by irregular diameters, fragility, leakiness, tortuosity, and abnormal blood flow. These characteristics prevent drug delivery, cause hypoxia, foster an immunosuppressive environment, and facilitate intravasation and metastasis. Recent studies have proposed that tumor-associated ECs play a more active role in TME remodeling and tumor progression than merely providing a physical channel. EC-derived surface proteins (e.g., VCAM-1) and soluble factors (e.g., CXCL2, soluble Notch ligands, LAMA1, and INHBB) orchestrate tumor cell behavior, promote cancer stem cell phenotype and survival, and recruit and educate tumor-associated neutrophils and macrophages.^451–454^ To achieve these above-mentioned protumor function, tumor ECs alters their gene expression program upon crosstalk with the tumor cells and other TME cells.^454,455^

Genome-wide DNA methylation profiling uncovered thousands of differentially methylated loci that contain hundreds of differentially expressed genes in human prostate tumor-derived ECs versus normal ECs.^456^ DNA hypomethylation of some proangiogenic genes (e.g., VEGF) caused by intracellular adenosine accumulation within ECs in a hypoxic environment promotes EC proliferation, migration, and angiogenic sprouting.^457^ DNA demethylation at the promoter region upregulates biglycan (a small leucine-rich secretory proteoglycan) expression in TECs from high metastatic tumors, which promotes tumor cell migration and intravasation through activating TLRs/ NF-κB/ ERK signaling pathway.^458^ *CYP24A1* is hypermethylated and silenced in the tumor ECs compared with that of normal ECs, which contributes to the selective calcitriol-mediated growth inhibition in ECs.^459,460^ However, the exact function of DNA methylation change in the *CYP24A1* gene in tumor vasculature remains unknown. Using DNMT and HDAC inhibitors, 81 genes including functionally validated anti-angiogenesis genes (*clusterin*, *fibrillin 1*, and *quiescin Q6*) were identified specifically and epigenetically silenced in tumor-conditioned versus quiescent ECs.^461^ Although the mechanism of DNMT-mediated gene silence remains elusive as no significant DNA methylation alteration was detected in the promoter CpG islands of seven tested genes, histone modification change, i.e., loss of H3 acetylation and H3K4 methylation, was proposed to be associated with the gene silence. Similarly, histone H3 deacetylation and H3K4 demethylation are involved in the silence of intercellular adhesion molecule-1 (ICAM-1) in tumor ECs, leading to reduced leukocyte-endothelial cell adhesion and consequently less inflammatory infiltration.^462^ In contrast, the expression of cell adhesion molecules in the ECs of distant tissues is crucial for circulating tumor cell adhesion, extravasation, and metastasis. Tumor cells induce an inflammatory response and consequent upregulation of cell adhesion molecules (e.g., E-selectin and VCAM-1) in the ECs at premetastatic sites.^463^ PRMT5-mediated methylation of the homeobox transcription factor HOXA9 is essential for the expression of cell adhesion molecules during the inflammatory response of human umbilical vein ECs.^464^ In addition, several histone methyltransferases (EZH2, G9a, and DOT1L) were shown to regulate the inflammatory response, cell cycle progression, viability,^465^ and angiogenesis of normal ECs, suggesting protein methyltransferases may play a more important role than currently appreciated in the regulating tumor ECs and tumor progression, which deserves further exploration. Although RNA methylation is relatively well studied in other systems (e.g., cardiovascular system) where it affects angiogenesis and vascular function, information about the potential role of m^6^A modification in tumor ECs is scarce.^466^ VEGF which has been considered the most crucial angiogenic growth factor is positively regulated by METTL3-catalyzed m^6^A modification in bone marrow stem cells.^467^ In tumor but not normal ECs, the m^6^A reader Hu antigen R (HuR) specifically accumulates in the cytoplasm to enhance the mRNA stability of VEGF and cyclooxygenase-2, promoting the survival, migration, and tube formation of tumor ECs.^468,469^ These results indicate DNA/RNA/protein methylation mechanism plays a key role in regulating the gene expression reprogramming and phenotypic plasticity of tumor ECs. Further studying of the methylation remodeling mechanism in tumor ECs may aid in developing new strategies to normalize or target tumor vasculature.

##### Fibroblasts

Cancer-associated fibroblasts (CAFs) are a major tumor stroma component (can reach 90% of tumor mass in pancreatic cancer) that participate in the TME shaping. They are responsible for the production of the bulk of extracellular components, including growth factors, cytokines, and extracellular matrix, as well as for ECM structure remodeling.^470^ Through these, CAFs stimulate tumor proliferation and metastasis, enhance angiogenesis, drive the desmoplastic reaction, suppress the immune response, and foster therapeutic resistance, resulting in poor prognosis of many tumor types. There are distinct subtypes of CAFs, including at least three main subtypes: myofibroblastic, inflammatory, and antigen-presenting subtypes that express α-smooth-muscle-actin, IL-6/LIF, and MHC class II, respectively.^471,472^ Identification of sub-subsets with specific markers (e.g., LRRC15 and NetG1) further subdivides the three main subtypes into more subpopulations, underpinning the complexity and heterogeneity of CAFs.^473,474^ Different external cues from TME drive the heterogeneity of CAFs. For example, tumor-derived IL-1 promotes LIF expression and differentiation into inflammatory CAFs, while TGFβ antagonizes this process by suppressing the expression of IL-1 receptor and facilitates differentiation into myofibroblastic subtype.^472^ In addition, multiple cells of origin generating CAFs contribute to heterogeneity and functional divergence, including tissue-resident fibroblasts, pericytes, mesenchymal stromal cells, and adipocytes.^475^ The genome of CAFs is stable, but their gene expression programs show highly heterogeneous. The transition from normal and quiescent fibroblasts into activated CAFs in TME involves distinct methylation alteration in DNA, histones, and likely RNAs.^476,477^

Similar to tumor cells, global DNA hypomethylation accompanying focal hypermethylation was observed in CAFs compared with that in normal fibroblasts.^478,479^ Specific genes that are hypomethylated, upregulated, and functionally validated in CAFs include *ADAM12*, *RUNX1*, *CXCR4*, and glycolytic genes (*PKM* and *LDHA*). ADAM12 functions in tumor-stromal cell interactions;^480,481^ RUNX1 mediates the transition from normal fibroblasts to CAFs;^482^ CXCR4 promotes tumor cell invasion probably through regulating the production of IL-8 in CAFs;^483^ PKM and LDHA, along with hypomethylated *HIF-1α*, promote glucose metabolic rewiring in CAFs to produce metabolites (lactate and pyruvate) that support anabolism of tumor cells.^484^ Independence on DNA synthesis and an increase in 5hmC level suggest the demethylation process involves TET-mediated active DNA demethylation mechanism.^485,486^ Indeed, tumor-derived lactate promotes α-KG synthesis in mesenchymal stem cells, facilitating TET activation and global demethylation reaction during differentiation into CAFs.^483^ On the other hand, the silence of negative regulators of CAF activation signaling is a prerequisite. STAT3 and TGFβ signaling pathways are essential for differentiation into inflammatory and myofibroblastic CAFs, respectively.^472^ DNA methylation-mediated silence of the negative regulators, *SHP-1* and *SOCS1*, is involved in the constitutive activation of STAT3 signaling, leading to the conversion of fibroblasts and sustained pro-invasive and pro-tumor phenotype of CAFs.^487–489^ *SMAD3*, an important transcription factor downstream of TGFβ signaling, is selectively hypermethylated in CAFs, which is linked with hyperresponsiveness to TGFβ signaling in terms of contractility and ECM deposition.^478^ Moreover, DNMT3B and TGFβ form a positive feedback loop through miR-200s/221, leading to a stably high level of DNMT3B expression and contributing to the maintenance of CAF phenotype.^490^ Methylation remodeling of histone constitutes another mechanism for maintaining the protumor capacity of CAFs. Loss of EZH2-mediated H3K27 methylation is implicated in the regulation of protumor secretome of CAFs, such as ADAMTS1, WNT5A, and IGF2 which facilitate cancer cell growth, migration, and invasion.^307,491^ Nicotinamide N-methyltransferase that consumes SAM to generate SAH cause histone hypomethylation and global gene expression alteration in CAFs, which is necessary and sufficient for the expression of CAF markers and secretion of protumor factors.^492^ The methylated product, 1-methylnicotinamide, can be taken up by infiltrating T cells and influences its tumor-killing function by inducing and reducing the expression of TNFα and IFNγ, respectively.^493^ Furthermore, the expression of lysine demethylases (e.g., KDM2A and LSD1) actively demethylates promoter-associated histones, rewiring the gene expression programs and promoting the conversion and function of CAFs.^494,495^ Although the current studies bias histone hypomethylation, histone methylation deserves further investigation as there is emerging evidence supporting the role of histone methylation gain (e.g., H3K4 methylation) in regulating CAF phenotype.^494^ Few studies focusing on the role of RNA methylation in regulating the differentiation and function of CAFs. METTL3-catalyzed m^6^A modification of *COL10A1* transcripts in CAFs enhances the mRNA stability and protein level, which protects lung squamous cell carcinoma from apoptosis-induced oxidative stress and promotes cancer cell proliferation.^496^ Zfp217-FTO-YTHDF2 axis is involved in the adipogenic differentiation of mouse fibroblast cells,^497^ suggesting a potential role of m^6^A remodeling in regulating CAF fate.

### Aging

Aging is characterized by functional decline with time at the molecular, cellular, tissue, and organismal levels, constituting a predominant risk factor for mortality and various diseases, such as cancer, diabetes, cardiovascular diseases, and neurodegenerative diseases. Twelve hallmarks of aging are recapitulated at the molecular and cellular levels: genomic instability, epigenetic alterations, telomere attrition, loss of proteostasis, mitochondrial dysfunction, deregulated nutrient sensing, stem cell exhaustion, altered intercellular communication, cellular senescence, disabled macroautophagy, chronic inflammation, and dysbiosis.^498,499^ Although these hallmarks are interconnected in a complex network, some degree of hierarchical relationship exists between them, with epigenetic changes at the top of the hierarchy.^498,500^ Studies in simple organisms (e.g., yeast, worms, and flies) lead to the proposition of the “information theory of aging”, which states that loss of epigenetic information and collapse of gene expression networks with time caused aging,^501–504^ and recently this theory has been experimentally validated in mammal.^505^

#### DNA methylation and aging

**Fig. 6:**
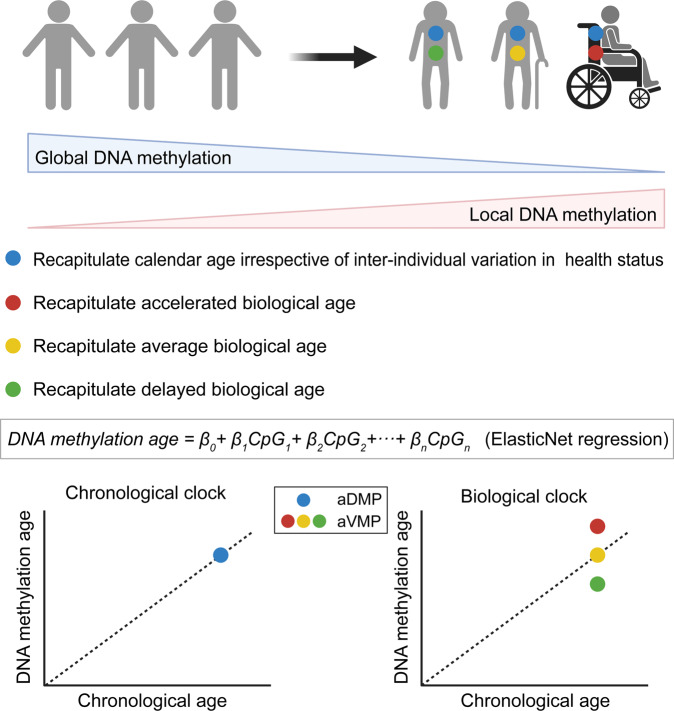
DNA methylation changes during aging and methylation clocks. Both global loss of and local gain of DNA methylation occur with age. age-associated differentially methylated positions (aDMPs) reflect an intrinsic age-related functional decline process occurring over chronological time in a population, while age-associated variably methylated positions (aVMPs) recapitulate the inter-individual heterogeneity in health status. A variety of DNA methylation clocks have been developed, generally using ElasticNet regression, to assess chronological age (chronological clocks) or biological age (biological clocks). A chronological clock can precisely predict calendar age irrespective of health conditions, while a biological clock can distinguish between people with different health conditions

As cells aging, genome-wide DNA methylation alteration occurs, featuring both loss of and gain of methylation at different sites (Fig. 6). The methylation alteration is conserved among species including mice, monkeys, and humans, and its rate is associated with lifespan.^506^ Loss of DNA methylation occurs primarily at repetitive DNA sequences where transposable elements enrich. These mobile sequences are highly methylated and form heterochromatin to suppress their activity within young cells. Hypomethylation contributes to heterochromatin loss and activation of (retro)transposition, leading to genomic instability and an increase in disease risk. As transposition-induced double-strand breaks can further aggravate loss of DNA methylation and heterochromatin through re-localization of the methyltransferases,^505,507,508^ loss of DNA methylation and activation of transposable elements may form a positive feedback loop. Reduced expression and/or activity of DNMTs contribute to the global demethylation process during aging. The expression of DNMTs responds to age-related decline of growth hormone, and their enzymatic activities are diminished due to reduced SAM/SAH ratio.^509^ Outside the heterochromatin regions, selected hypomethylation occurs in regulatory elements of genes (e.g., enhancers and promoters), resulting in elevated expression of the functional genes. Lineage-specific transcription factor binding and chromatin states in young cells facilitate gene demethylation with aging in specific cell types, such as *ITGAL* in T cells and *Nkx6-1* in pancreatic β cells.^510,511^ Hypermethylation and age-associated heterochromatin formation usually occur at CpG-island-containing genes, such as metabolism-associated genes and cell-cycle genes, contributing to metabolic dysfunction and permanent close of the proliferation program, respectively.^510,512,513^ Genome-scale studies of DNA methylation dynamics in stem cells revealed that Polycomb target genes are preferentially hypermethylated with age, contributing to stem cell exhaustion during aging.^514,515^ Two alternative theories have been proposed for the hypermethylation of Polycomb target genes: cooperation, or competition between de novo DNA methyltransferases and Polycomb complexes, which remains further clarification.^509^ Since these hyper- or hypomethylated CpG sites show predictable and consistent shifts in average methylation level as individuals age, they are termed age-associated differentially methylated positions (aDMPs) or differentially methylated regions (aDMRs) that involve multiple contiguous CpGs. aDMPs and aDMRs might link to genes and pathways (as mentioned above) involved in an intrinsic age-related functional decline process occurring over chronological time^516^ (Fig. 6).

Unlike aDMPs, there are CpG sites that show an increase in methylation variance with age, while the mean methylation level does not necessarily shift. These sites are referred to as age-associated variably methylated positions (aVMPs) or variably methylated regions if implicate multiple contiguous CpGs.^516^ Such methylation drifts with aging was first reported in studies of monozygotic twins, in which inter-individual variation in both global and locus-specific methylation is larger in the elderly than in the young.^517^ aVMPs represent a stochastic alteration of methylation accumulated during age as a result of differential environmental stimulation, contributing to deviation of biological age from chronological age. That is, aVMPs reflect inter-individual heterogeneity in health status (Fig. 6). Along with hypermethylated or hypomethylated CpG sites that converge on the mean (i.e., methylation level transition from 0→50% or 100→50%), aVMPs increase epigenetic disorder as measured by Shannon entropy.^516,518^ The heterogeneous methylation landscape is correlated with increased gene expression variability in cells and tissues of elders, including stem cells, pancreas, heart, and immune cells.^519–522^ Notably, this increased gene expression noise is an intrinsic mechanism of cellular aging, independent of cellular composition.^520,521^ Furthermore, some aVMPs are associated with gene expression in trans, which may affect fundamental aging mechanisms including DNA repair, apoptosis, and cellular metabolism.^518^ These results underscore the close correlation between DNA methylation and age, and the deconvolution of individual CpG sites facilitates the development of age-tracking clocks (see below).

Not only correlation but also DNA methylation change is a causative factor for aging and rejuvenation. DNMT1 haploinsufficiency-mediated insufficient DNA methylation promotes age-related diseases in mouse model.^523^ Ectopic DNA methylation interferes with the expression and function of pro-longevity transcription factors, such as FOXO3A and NRF2,^500,524,525^ indicating the essential role of DNA methylation homeostasis for lifespan. In line with this, centenarians and supercentenarians show younger-than-expected DNA methylation states at hundreds of CpG sites enriched for neuropsychiatric- and cancer-related genes,^526^ and prevention of age-related DNA methylation alteration contributes to successful age-delaying interventions, such as caloric restriction.^512,527^ More direct evidence that supports DNA methylation-mediated rejuvenation comes from the cellular reprogramming experiment in aged mice, in which ectopic expression of Yamanaka factors (OCT4, SOX2, KLF4) recovers youthful DNA methylation pattern and improves tissue function in a TET1/2-dependent manner.^528^ The expression and activity of TETs are also induced in other rejuvenating strategies in human cell and mouse models, including caloric restriction, heterochronic parabiosis, and metformin treatment.^512^ Moreover, a recent study showed that TET3-catalyzed 5hmC functions to inhibit intragenic transcription and assure transcriptional fidelity in airway smooth muscle cells, which prevents chronic inflammation in the lung.^529^ As loss of faithful transcription and increase in inflammation are drivers of aging,^498,530^ the discovery implies the additional benefit of TET expression for age-delaying.

DNA methylation is the best characterized age-associated epigenetic biomarker, and the DNA methylation clock (also known as epigenetic clock) is currently the most promising biological age predictor.^531^ It is built upon a linear regression model, usually, ElasticNet regression (Fig. 6). In the principle of the minimal cost function, informative CpG sites are selected and endowed with positive or negative coefficients, while non-informative CpG sites get the coefficient value of 0. The sum of each product of individual methylation level with its coefficient gives the estimated age. There are a variety of DNA methylation clocks developed with different characteristics and purposes, which can be grouped into three categories: chronological clocks, biological clocks, and hybrid chronological-biological clocks^532^ (Fig. 6). Chronological clocks, the first-generation DNA methylation clocks, are trained on exclusively on calendar age, such as Horvath’s pan-tissue clock and Hannum’s blood-specific clock which adopt 353 and 71 CpG sites, respectively.^533,534^ Profiling DNA methylation age at the single-cell level leads to the development of a statistical framework for the single-cell epigenetic clock, namely scAge, which recapitulates the chronological age of tissues and exhibits heterogeneity among cells.^535^ These chronological clocks predict calendar age with high accuracy (Pearson correlation between predicted age and chronological age can be over 0.9 in test data of Horvath’s and Hannum’s studies), even near-perfectly when enough training samples are available.^533,534,536^ Since calendar age per se is a strong predictor of organismal functional state and has biological meaning, residual values of estimated DNA methylation age by these chronological clocks and chronological age can be used to predict all-cause mortality to a certain degree.^537^ Lower DNA methylation age than chronological age was observed in centenarians and supercentenarians.^526,538^ Nevertheless, the capacity of chronological clocks to distinguish between healthy versus unhealthy individuals of the same calendar age is still limited and unsatisfactory, and increased prediction of chronological age accompanies decreased association with mortality.^532,536,539^ To deal with the problem, biological clocks, such as GrimAge and DunedinPACE, focus on biological phenotypes rather than calendar age, which enables them to predict age-related morbidity and all-cause mortality better than chronological clocks.^540,541^ GrimAge integrates DNAm surrogates (containing 1030 CpG sites) of seven plasma proteins and smoking pack-year to predict lifespan,^542^ and DunedinPACE trains DNA methylation data (containing 173 CpG sites) on a composite estimator of aging that reflects 19 biomarkers of organ-system integrity, including cardiovascular, immune, metabolic, hepatic, renal, dental, and pulmonary systems.^541^ Hybrid clocks track both chronological and biological age, as exemplified by PhenoAge. To build PhenoAge, DNAm data were trained on a combined phenotypic age estimator consisting of chronological age and nine biomarkers related to lifespan and healthspan, and 513 CpG sites were selected.^543^ Compared with chronological and biological clocks, hybrid clocks display intermediate prediction power for age-related diseases and lifespan.^540,541^ The development of these DNA methylation clocks greatly facilitates the evaluation of the aging rate and screening of potential anti-aging treatments.

The selected CpG sites and epigenetic clock scores are mathematical products, while the biological meaning and mechanism of epigenetic clock ticks remain elusive. Indeed, many CpG sites of epigenetic clocks are not located within genes, and their methylation changes are not strongly associated with gene expression.^544^ In addition, the linear models of epigenetic clocks presume that DNA methylation changes with age at a constant rate. In contrast, there are at least two nonlinear patterns where the rate of age-related DNA methylation changes is variable and age-dependent. The first pattern describes that some CpG sites exhibit rapid DNA methylation changes in early life and the change rate is stabilized in later life, which may be associated with the rapid development in early life.^533,545^ The second pattern describes a reverse mode: stable DNA methylation level in early life followed by rapid change in late life.^546^ CpG sites with a second pattern may be more pertinent to senescence and age-related diseases as a result of biological roles performed by the resident genes. For example, two CpG sites whose DNA methylation levels increase with age at an increasing rate are identified in the promoter region of *KLF14*.^546^ Since KLF14 directly regulates Treg cell differentiation via suppressing *FOXP3* expression,^547^ hypermethylation and silence of *KLF14* may contribute to increased levels of Treg cells and immunosenescence.^546,548^ Understanding what drives DNA methylation clocks tick and reconciling the current linear model with the non-linear aging process may shed light on the aging mechanisms and encourage the design of new age-tracking clocks.

#### Histone and non-histone protein methylation in aging

**Fig. 7:**
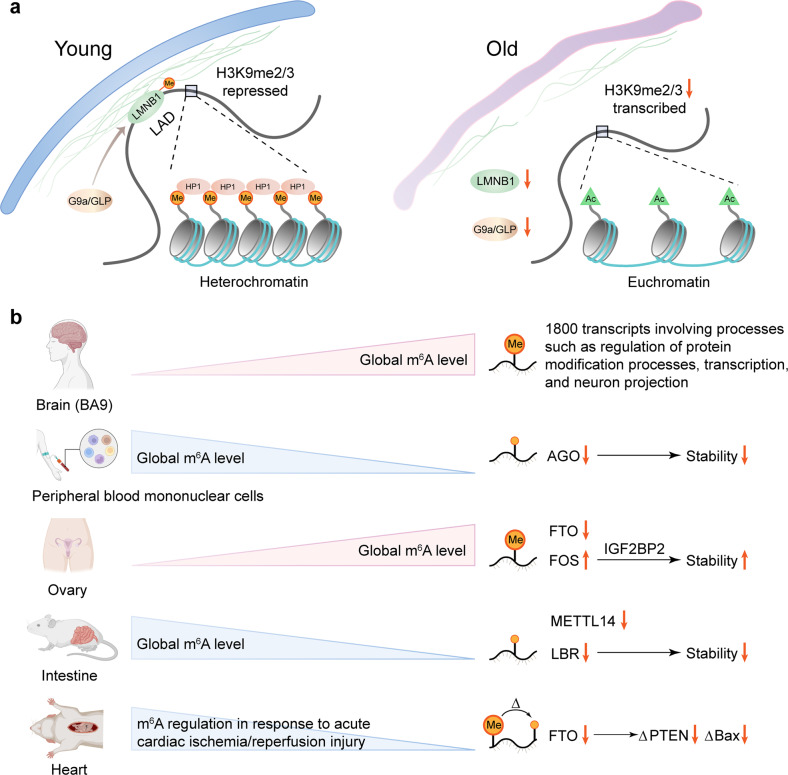
Histone, nonhistone protein, and RNA methylation changes with age. **a** Alterations in histone and nonhistone protein methylation and 3D genome structure with age. G9a/GLP-mediated methylation of Lamin B1 (LMNB1) promotes heterochromatin assembly at the nuclear periphery and formation of lamina-associated domains (LADs) in young cells, which ensures transcriptional silence; whereas defects in both histone methylation (H3K9me2/3) and nonhistone protein methylation are associated with chromatin detachment from the nuclear lamina and a shift to a euchromatin state. **b** Overall changes of m^6^A modification with age in different tissues including brain, peripheral blood mononuclear cells, ovary, and intestine, and crucial age-related targets. Changes in the expression of writers or erasers account for the methylation fluctuation. Although the m^6^A level and expression of modifiers do not differ significantly between young and old mouse hearts, they do show aging-related differences in response to acute cardiac ischemia/reperfusion injury, which may be associated with reduced tolerance to ischemic injury with age

Intertwined with DNA methylation, histone methylation, especially at H3 lysines 4, 9, 27, and 36, changes and play a role during aging. The sum of these methylation changes links to common alterations in 3D genome structure with age, featuring loss of heterochromatin in repressive compartments, weakened euchromatin in active compartments, interfacing topological compartment switch, and elevated epigenetic disorder^549^ (Fig. 7a). Both H3K9me2 and H3K9me3 are histone marks for constitutive heterochromatin, and their levels change during aging. In human fibroblasts, the expression of methyltransferases for H3K9me2, G9a/GLP heterodimer, is reduced in an age-dependent manner, resulting in global reduction of H3K9me2 in the physiologically aged cells^550^ (Fig. 7a). Tethering H3K9me2-marked heterochromatin to the nuclear lamina, a structure called lamina-associated domain (LAD), is essential for gene regulation and lifespan.^549,551^ In addition to histone substrate, G9a/GLP methylate Lamin B1 (LMNB1) at K417, a component of the nuclear lamina, to facilitate the LAD formation by modulating the stability and localization of LMNB1 in young human fibroblasts, whereas this process is dampened in the old cells due to G9a/GLP deficiency^550^ (Fig. 7a). Studies in worms demonstrate H3K9me2 is required for lifespan extension induced by mitochondrial stress or specific gene mutations in components of the H3K4 methyltransferase complex (i.e., *set-2*, *ash-2*, and *wdr-5*),^552,553^ supporting a positive role of H3K9me2 in longevity regulation. However, a recent study showed H3K9me2 and its methyltransferases restrict the lifespan of long-lived worms with a mutation in insulin-like receptor gene *daf-2*.^554^ It will be intriguing to see whether such genomic context-dependent regulation of longevity by H3K9 di-methylation works in mammals. A decline in the global level of H3K9me3 was observed during normal mammalian aging, as well as in patients with premature aging diseases, such as Hutchinson-Gilford progeria syndrome (HGPS) and Werner syndrome.^500,555,556^ The expression of SUV39H1, an H3K9me3 methyltransferase, decreases with age in hematopoietic stem cells (HSC), contributing to perturbed heterochromatin and decreased B lymphopoiesis in the elders.^557^ Paradoxically, SUV39H1 is stabilized by progerin in a progeria mouse model (*Zmpste24* mutation) that mimics HGPS, leading to increased H3K9me3, which dampens DNA repair and shortens lifespan.^558^ A decrease in global H3K9me3 level is involved in the α-KG-mediated rejuvenation of bone marrow MSCs, which ameliorates age-related osteoporosis in mice.^559^ Region-specific conversion of H3K9me2 to H3K9me3 and loss of H3K9me3 occur in somatic tissues of aged worms, potentially contributing to aging phenotype.^560^ Considering the distinct role of H3K9me2 and H3K9me3 in genomic structural organization,^561^ these results suggest the different role of the two repressive histone marks in the regulation of lifespan, as well as the complex mechanism of action for H3K9me3. Consistently, the H3K9me3 level decreases in the aging intestine of flies,^562^ while its level increases in the aging brain, accompanied by changes in its distribution.^563^ Single-cell epigenome analysis of mouse brain shows age-related loss of H3K9me3 occurs in the excitatory neurons but not in inhibitory neurons or glial cells, that is, cell-type specific alteration of this mark during the organ aging.^564^ Both H3K9me2 and H3K9me3 are implicated in the repression of senescence-associated secretory phenotype (SASP) genes.^565^ Therefore, further studies are needed to untangle to what extent the function of H3K9me2 and H3K9me3 is overlapped and divergent, as well as to clarify their tissue-specific and context-dependent mechanism in longevity regulation.

In line with the heterochromatin loss theory of aging, H3K27me3 which marks facultative heterochromatin acts as a positive regulator of lifespan. In worms, increasing the global H3K27me3 level by knockdown of H3K27me3 demethylase UTX-1 extends lifespan.^566,567^ A global higher level of H3K27me3 contributes to a stable epigenome and exceptional longevity in naked mole rats, compared with that in mice.^568^ The total H3K27me3 level is reduced with age in worms, senescent mammalian fibroblast cells, and progeroid mammalian cells (e.g., HGPS patients and engineered ICE mice).^505,556,567,569^ Down-regulation of the methyltransferase EZH2 is implicated in the decline of H3K27me3 level in these normal and pathological aging mammalian cells.^556,569^ Loss of H3K27me3 facilitates transcriptional activation of multiple pro-aging genes/pathways, such as SASP genes, insulin/IGF-1 signaling pathway, cell identity-related genes, and p16^INK4A^.^505,566,569,570^ In contrast, there’s plenty of evidence for a negative role of H3K27 tri-methylation in longevity regulation. H3K27me3 increases with age in flies, murine HSCs and quiescent satellite cells, and brain tissues of senescence-accelerated prone mouse 8.^571–574^ Deposition of H3K27me3 dampens a battery of stress responses (e.g., heat shock response, oxidative stress response, and mitochondrial unfolded protein response) and is detrimental to lifespan in worms and flies, which is rescued by overexpressing the H3K27me3 demethylases or downregulating PRC2 complex, leading to a longer lifespan.^575–577^ The correlation of the murine demethylase homologs (PHF8 and KDM6B) with longevity and mitochondrial stress response was also observed.^575^ Furthermore, enhancing glycolysis is helpful for longer and healthier lifespans in flies and humans, whereas the expression of glycolytic genes is suppressed with age by H3K27me3 deposition in *Drosophila*.^571,578^ Therefore, the positive or negative regulatory role of H3K27 tri-methylation in longevity is dependent on the specific loci and cell types. Since both genome-wide gain and loss of H3K27me3 can occur in aging mammalian cells,^570,573^ it is conceivable the net impact of the redistribution on aging relies on the specific context. In addition, lifespan extension in worms by overexpressing the H3K27 demethylase JMJD-3.2 does not require its catalytic activity.^579^ Similarly, upregulation of KDM6A ameliorates age-induced cognitive deficits in male mice independent of its demethylase activity,^580^ providing an additional regulatory layer in age and age-related diseases by H3K27 methylation modifiers.

Like H3K27me3, the role of H3K4me3 in lifespan regulation is highly context-dependent, and both positive and negative roles have been observed. In *C. elegans*, downregulation of H3K4me3 level, by disrupting the methyltransferase complex (e.g., SET-2), transient exposure to ROS during early development, or metformin treatment, extends lifespan, while upregulation of the mark via inactivating the demethylases (RBR-2 or SPR-5) shortens lifespan.^581^ The pro-longevity effect of H3K4me3 deficiency in worms is dependent on the presence of an intact adult germline, germline-intestine communication, accumulation of mono-unsaturated fatty acids, and increased stress resistance.^581–584^ Similarly, the global H3K4me3 level is sensitive to ROS and negatively regulates the stress resistance in mammalian cells.^584^ Elevation of H3K4me3 level with age is observed in murine HSCs and human immune cells.^573,585^ Furthermore, the H3K4me3 level and its methyltransferases are upregulated in the prefrontal cortex of patients and mouse models with Alzheimer’s disease (AD), and pharmacological targeting of the SET1/MLL HMTs ameliorates cognitive and synaptic deficits in AD mice.^586^ Specific gene targeted for H3K4 methylation during mammalian aging is exemplified by *WTAP*: downregulation of the demethylase KDM5A in senescent nucleus pulposus cells facilitates H3K4me3 deposition at the *WTAP* promoter and enhances the gene expression, promoting the progression of intervertebral disc degeneration (IVDD), one of the most strongly age-associated degenerative diseases.^587^ Paradoxically, knockdown of H3K4 demethylases (RBR-2 or LSD-1) is sufficient to extend longevity in worms,^588,589^ and a recent study showed a decrease in H3K4me3 level by mutation of the methyltransferase SET-2 shortens lifespan, accompanying with loss of fertility but normal intestinal fat stores.^590^ In humans, the level of H3K4me1 and H3K4me3 is decreased in aged HSCs,^591^ and the number of genes with H3K4me3 loss surpasses that of genes with H3K4me3 gain (in a 6:1 ratio) during postnatal development and aging of prefrontal neurons,^592^ implying a pro-aging effect of H3K4me3 deficiency. Multiple sources of variation, such as genetic backgrounds, uncontrollable daily fluctuations in the microenvironment, and quality and purity of reagents, can cause stochasticity in aging phenotype and potentially contributes to the different lifespan extension outcomes in the model organisms.^590,593,594^ In addition, studies in yeast revealed accumulation of H3K4me3 with age contributes to loss of rDNA heterochromatin and genome-wide increase in pervasive transcription that potentially impairs lifespan, meanwhile maintaining normal lifespan by facilitating the expression of many genes crucial for healthy aging, such as NAD+ biosynthesis genes and histone genes.^595,596^ Therefore, beneficial and detrimental effects of H3K4 methylation on aging phenotype are potentially influenced by the multiple variable sources and disproportionally manifested at cell, tissue, and organismal levels, the combination of which biases longevity outcomes.

H3K36me3 was initially reported to be a pro-longevity histone mark in yeast, worms, and flies. Genetic studies in these simple organisms showed that loss of H3K36me3 by inactivating the methyltransferase shortens lifespan, while gain of H3K36me3 by deleting the demethylase extends lifespan.^597,598^ Mechanically, H3K36me3 at gene bodies prevents cryptic transcription^597^ and/or limits transcriptional change with age.^598^ A similar mechanism was recently demonstrated in murine hematopoietic and neural stem cells and human mesenchymal stem cells, in which loss of H3K36me3 within gene bodies during aging compromises transcriptional fidelity.^530^ Specifically, the recruitment of H3K4me3 demethylase and de novo DNA methyltransferases is suppressed in the absence of H3K36me3, generating intragenic permissive chromatin state (i.e., accumulation of H3K4me3 and unmethylated CpGs) that supports cryptic transcription in the aged mammalian stem cells. The level of H3K36me3 also declines with age in brain tissues of a senescence-accelerated mouse model.^574^ SETD2-mediated H3K36 trimethylation promotes osteogenesis of bone marrow mesenchymal stem cells (MSCs) by facilitating the transcriptional initiation and elongation of the *Lbp* gene in mouse models, and such axis is decreased in aged bone marrow, which may contribute to age-related osteoporosis.^599^ The DNA methylation age is substantially accelerated in patients with loss-of-function mutations in the H3K36 methyltransferase NSD1.^600^ These results indicate H3K36 methylation-mediated transcriptional regulation is an important and conserved anti-aging mechanism from yeast to humans.

In addition to protein lysine methylation, protein arginine methylation and methyltransferases (PRMTs) are involved in lifespan regulation. PRMT1 is a positive regulator of stress tolerance and lifespan in worms and moths by asymmetrically methylating the crucial pro-longevity transcription factors, DAF16/FOXO and SKN-1/NRF.^601–603^ Asymmetric arginine methylation that is catalyzed by type I PRMTs is downregulated during replicative and H_2_O_2_-induced premature senescence in human fibroblasts.^604^ The expression of three PRMT family members (PRMT1, PRMT4, and PRMT5) in rats is tissue-specific and age-dependent, suggesting tissue-specific role of PRMTs in the aging process of different tissues.^605^ Consistently, tissue-specific expression of PRMT8 and proper accumulation of asymmetric dimethyl arginines in murine postmitotic neurons are required for protection against the age-related increase in DNA damage and cell death.^606^ As PRMT activity against histone mixtures changes when tissues age,^605^ future studies should determine whether PRMTs transcriptionally regulate age-related genes directly by histone arginine methylation.

#### RNA methylation and aging

The global m^6^A RNA modification is diminished in peripheral blood mononuclear cells of elders and in replicative senescent human fibroblasts, which is associated with changes in the expression of AGO2 and the abundance of miRNAs^607^ (Fig. 7b). The protein level of m^6^A methyltransferases (METTL3 or METTL14) is reduced in the fibroblasts and mesenchymal stem cells (MSCs) derived from HGPS patients, due to the loss of Lamin A-mediated protection from proteasomal degradation.^608,609^ Cell cycle-related genes, including *MIS12*, are enriched in the subset of demethylated mRNAs, contributing to premature aging.^608^ Depletion of METTL3 and RNA m^6^A was also observed in MSCs of individuals with Werner syndrome,^608^ suggesting dysfunction of the m^6^A modification pathway is common during normal and premature aging with different etiological factors. In line with this, de-repression of endogenous retroviruses (ERVs) is common during aging and contributes to cellular senescence, while the m^6^A-YTHDF pathway directly restricts mRNAs of ERVs (e.g., intracisternal A-particles) to maintain cellular integrity.^610^ Overexpression of METTL14 alleviates replicative senescence and premature senescence in human skin fibroblasts and *Zmpste24*^−^/^−^ mouse embryonic fibroblasts, respectively,^609^ while upregulation of demethylase FTO is implicated in the IL-17A-induced cellular senescence of human endothelial cells.^611^ On the other hand, m^6^A modification is enhanced during senescence and contributes to senescence and age-related diseases. For example, METTL3 expression and m^6^A level are elevated in fibroblast-like synoviocytes (FLS) of patients with osteoarthritis, which inhibits autophagy and promotes senescence of the cells by targeting ATG7 mRNAs (an enzyme crucial for the formation of autophagosomes) for degradation.^612^ Inhibition of METTL3 ameliorates the FLS senescence and osteoarthritis progression in mouse models. WTAP- or METTL14-mediated m^6^A modification of non-coding RNAs, such as lncRNA NORAD and miRNA miR-34a-5p, promotes senescence of nucleus pulposus cells (NPCs) and development of IVDD.^587,613^ Things are more complex. Low fluid shear stress induces redistribution of more than one thousand m^6^A peaks, in which hyper-/hypomethylated genes are enriched in aging-related (e.g., mTOR, insulin, and ERRB) and oxidative stress-related (e.g., HIF1A, NFE2L2, and NFAT5) pathways, which mediates the cellular response to oxidative stress and senescence.^614^ ALKBH5-mediated demethylation of DNMT3B transcripts increases DNMT3B expression, which in turn downregulates E4F1 expression by DNA methylation, contributing to NPC senescence and IVDD degeneration.^615^ These results suggest that m^6^A RNA modification works in a cell-type-specific and context-dependent manner to promote or delay cellular senescence.

Unlike the relatively well-studied cellular senescence, the studies about the role of m^6^A RNA methylation, especially mRNA methylation, in the aging of organs and organisms are in their infancy. This is partly due to the absence of genes encoding mRNA m^6^A methyltransferases METTL3/METTL14 in *C. elegans*, the most used models for organismal aging. However, two rRNA m^6^A methyltransferase genes *ZCCHC4* and *METTL5* in their genome regulate lifespan.^616,617^ *zcchc-4* mutation extends worm longevity under homeostatic conditions while *mettl5* mutation does it under stress conditions, which is associated with translational adjustment to cope with endogenous and environmental stresses.^616–618^ METTL5-deficient mice show abnormal morphology and behavior,^619^ nevertheless, whether mammalian rRNA m^6^A methyltransferase homologs regulate aging and lifespan remains unknown.

The correlation of mRNA m^6^A modification pathway with the aging of certain mammalian organs, including intestine, ovary, heart, and brain, has been recorded (Fig. 7b). The levels of m^6^A modification decrease in mouse intestine as a consequence of aging.^620^ Further study demonstrated METTL14-mediated m^6^A methylation is required for intestinal integrity and normal lifespan of *Drosophila melanogaster*, partly through the downstream target, Lamin B receptor (LBR).^620^ Reduced expression of FTO and ensuing elevated m^6^A level occur in the aged mouse ovary, as well as in granulosa cells of the aged human ovary.^621,622^ Consequently, the degradation of a key downstream target mRNA, FOS, is interrupted with the help of reader protein IGF2BP2, contributing to ovarian aging.^621^ Although the m^6^A level and expression of modifiers do not show significant differences in young and old mouse hearts, they exhibit aging-related differences in response to acute cardiac ischemia/reperfusion injury, which might be associated with reduced tolerance to ischemic injury with age.^623^ The potential role of m^6^A in brain aging acquires the most attention, as m^6^A methylation strongly associates with brain development and function.^624^ The level of m^6^A methylation is increased with age in the human brain tissue region BA9.^625^ A similar trend occurs in the mouse cerebral cortex and hippocampus.^625,626^ In contrast, a recent study reported an opposite trend across mouse brain regions.^627^ Abnormal m^6^A methylation has been observed in neurodegenerative diseases, such as Parkinson’s disease (PD) and AD.^625,628^ The precise mechanisms, including directionality, magnitude, and functional consequences of m^6^A alterations, are controversial,^625,627,629–631^ with the latest studies supporting human AD is associated with loss of m^6^A methylation.^627,632^ Moreover, upregulation of METTL3 alleviates AD in both in vitro and in vivo models,^632^ indicating m^6^A methylation matters in brain aging and neurodegenerative diseases and serves as a potential therapeutic target. In addition, an intron sequence polymorphism of the *YTHDF2* gene with enhanced transcription is associated with exceptionally long lifespan in humans, implying high expression of YTHDF2 is beneficial for longevity.^633^ Consistently, YTHDF2 is responsible for the degradation of m^6^A-modified mRNAs encoding inflammatory pathway genes and maintains HSC function upon aging in mice,^634^ and its upregulation underlies the anti-senescence effects of melatonin or senolytics cocktail (dasatinib and quercetin) in human cells by suppressing the MAPK-NF-κB pathway.^611,635^ The above preliminary results warrant future studies to clarify whether changes in the m^6^A methylation pathway have a causative relationship with mammalian aging.

The role of RNA m^5^C methylation in senescence and aging is emerging, implicating nearly all types of RNAs, i*.*e., rRNAs, tRNAs, miRNAs, mRNAs, and lncRNAs. Two rRNA m^5^C methyltransferases, NSUN-1 and -5 that each methylate one of the two known m^5^C sites in the large ribosomal subunit, play an important role in the lifespan regulation in simple organisms. Deficiency of NSUN5-mediated rRNA m^5^C modification extends lifespan in yeast, worms, and flies, which is associated with the enhanced translation of stress-responsive mRNAs as a result of altered local ribosome structure and translational fidelity.^636^ Soma-specific rather than whole-animal knockdown of NSUN-1 in worms prolongs lifespan, accompanied by reduced body size and impaired fecundity.^637^ While NSUN5 knockout mice show a decrease in body weight, lean mass, and protein synthesis in several tissues, whether NSUN-1 and −5 regulate the lifespan of mammals remains future clarification.^638^ The two tRNA methyltransferases, DNMT2 and NSUN2, stabilize m^5^C-modified tRNAs by protecting them from angiogenin-mediated cleavage and promoting stress tolerance.^639^ Overexpression of DNMT2 extends the lifespan of flies,^640^ while the silence of DNMT2 suppresses proliferation and induces senescence in human fibroblasts.^641^ Loss of NSUN2-mediated tRNA m^5^C modification contributes to neurodevelopmental disease as a result of an accumulation of tRNA-derived small RNA fragments.^642^ Beyond m^5^C methylation and tRNA substrates, NSUN2 plays protective roles in preventing neurodegeneration (e.g., AD) and delaying replicative senescence, through targeting miRNAs (miR-125b) and mRNAs (p27 and CDK1) for m^6^A and m^5^C modification, respectively.^643,644^ Unlike generally downregulated during replicative senescence, the NSUN2 expression is stimulated in the stress conditions (e*.*g., oxidative stress and high glucose level), leading to premature senescence of cells.^645,646^ Such processes involve downstream senescence-related mRNAs, including SHC, p21, and p16, and the m^5^C methylation promotes their stability and/or translation.^645–647^ Interestingly, NSUN2-mediated-m^5^C and METTL3/14-mediated m^6^A modifications mutually promote each other at the 3′UTR of p21, which synergistically enhances the translation of p21, promoting oxidative stress-induced cellular senescence.^645^ In addition, m^5^C modification at the lncRNA subunit TERC of telomerase holoenzyme is necessary for telomerase function,^648^ and interruption of the RNA methylation causes telomere attrition, driving replicative senescence of mouse and human cells.^648,649^ The RNA methyltransferase responsible for the methylation of lncRNA TERC is yet to be investigated.

## Targeting methylation for therapy

### Targeting aberrant DNA methylome

Two types of small molecular inhibitors are developed to inhibit DNMTs: nucleoside analogs and non-nucleoside molecules. The nucleoside DNMT inhibitors, including azacitidine (5′-azacytidine) and decitabine (5-aza-2′-deoxycytidine), are incorporated into replicating DNA, resulting in covalently sequestering DNMTs (DNMT1, DNMT3A, and DNMT3B), degradation of these enzymes by proteasome, and DNA damage. Azacytidine is also integrated into RNA, inhibiting protein synthesis. Therefore, these inhibitors cause direct cytotoxicity at high doses and are used at low concentrations to induce genome-wide DNA demethylation. Azacytidine and decitabine were approved in the early 2000s for the treatment of myelodysplastic syndrome (MDS), acute myeloid leukemia (AML), and chronic myelomonocytic leukemia.^650^ The anti-cancer activities of the DNMT inhibitors involve both directly targeting tumor cells and enhancement of antitumor immunity. A direct consequence of DNA demethylation is the reactivation of silenced tumor suppressor genes, leading to cell-cycle arrest, differentiation, and apoptosis.^651^ De-repression of MHC-I genes and cancer/testis antigens promote T cells to recognize cancer cells, and de-repression of ERVs induces a state of viral mimicry in cancer cells, which induces type I interferon signaling and facilitates immune elimination. As mentioned above DNA methylation dynamics is important for the function of nontumor cells within TME, and the anti-cancer effects of DNMT inhibitors are associated with the modulation of these cells. For example, decitabine treatment promotes CD8^+^ T cell activation and effector function, which is due in part to the overexpression of NFATc1 short isoforms and reversal of exhaustion-associated DNA methylation program.^318,652^ Demethylation-mediated expression reprogramming rather than direct cytotoxicity is consistent with the prolonged time for initial response.^653^ Although a positive response to azacytidine treatment is well-documented in patients with MDS, the hypomethylating agent causes demethylation and upregulation of an oncogene (*SALL4*) in 30–40% of patients, associated with a worse outcome.^653,654^ That is, DNMT inhibition activates not only tumor suppressor genes but also oncogenes, discounting the therapeutic effect of global demethylation. Moreover, these nucleoside DNMT inhibitors show limited clinical efficacy as monotherapy in solid cancers. DNMT inhibition stimulates the recruitment of MDSCs to TME, dampening antitumor immunity.^655^ Besides the tumor-intrinsic mechanisms, low chemical stability and poor pharmacokinetic properties of the nucleoside DNMT inhibitors also account for the limited clinical efficacy.

To overcome the poor pharmacokinetic properties and dose-limiting toxicity of nucleoside analogs, non-nucleoside DNMT inhibitors that directly block the catalytic activity are pursued. RG-108, one of the first nonnucleoside inhibitors, can effectively inhibit bacterial M.SssI CpG methyltransferase (half maximal inhibitory concentration (IC_50_) = 0.012 μM),^656^ while was reported later with reduced potency against human DNMT1 (half maximal effective concentration (EC_50_) = 390 μM).^657^ Quinoline-based inhibitors, such as SGI-1027 and its analog MC3343, inhibit DNMT1 and DNMT3A far more potent than RG-108.^657^ The antitumor effect of MC3343 was confirmed in an osteosarcoma patient-derived xenograft.^657^ Intriguingly, there is an inverse relationship between MC3343 sensitivity and DNMT1/3A expression (*r* > 0.6), but not for the traditional inhibitor decitabine, nevertheless this relationship needs further validation.^657^ GSK3685032 which contains a dicyanopyridine moiety is a potent first-in-class selective inhibitor of DNMT1 (IC_50_ = 0.036 μM). It works by competing with the active-site loop of DNMT1 for penetration into hemimethylated DNA between two CpG base pairs.^658^ Compared with decitabine, GSK3685032 exhibits improved tolerability and efficacy in mouse models of AML and may provide more benefit in the clinic.^658^ Following studies show the demethylation and antitumor effect of GSK3685032 in peripheral nerve sheath tumor and colorectal cancer in vitro or in vivo,^659,660^ and identify several additional dicyanopyridine-containing DNMT1-selective, nonnucleoside inhibitors.^661^ Some old drugs, such as hydralazine, procainamide, and epigallocatechin gallate, are repurposed to inhibit DNMT activity and reactivate silenced genes in cancer or noncancer cells.^662–664^ Epigallocatechin gallate, a major polyphenol from green tea, competitively represses DNMT activity possibly by forming hydrogen bonds with the five key amino acids in the catalytic pocket.^662^ A different study shows epigallocatechin gallate possesses significant cytotoxicity and genotoxicity in human cancer cell lines, but fails to cause significant demethylation of genomic DNA.^665^ Epigallocatechin gallate also can inhibit HDACs and regulate noncoding RNA networks in different types of cancer.^666^ A similar pleiotropic effect likely applies to other repositioned old drugs.

While DNMT inhibitors have been used to target aberrant DNA methylation for the treatment of TET2, IDH1, and IDH2 mutant diseases,^667,668^ specific agonists or inhibitors are developed (Table 1). Vitamin C is crucial for the TET-mediated oxidation of 5mC via physical interaction with the TET catalytic domain and providing an electron to reduce Fe^3+^ to Fe^2+^.^669^ Vitamin C treatment mimics the effects of TET2 restoration in the *Tet2*-deficient mouse model of leukemia, which promotes DNA demethylation and suppresses leukemia progression.^670^ The majority of TET2 mutations in AML are heterozygous, making vitamin C a potent hypomethylating agent for therapy.^671^ In a 1-year clinical trial with oral 1 g/day vitamin C supplementation, the proportion of hypermethylated loci is reduced, accompanied by reduced gene expression divergence between lymphoma predisposition *TET2*^*+/−*^ and control *TET2*^*+/+*^ individuals.^672^ Two clinical trials are engaged using vitamin C alone or in combination with azacytidine in MDS patients with *TET2* mutations, i*.*e., NCT03433781 (Recruiting) and NCT03397173 (Completed, yet no result has been published). TET2 loss-of-function mutations and IDH1/2 neomorphic mutations are mutually exclusive in the AML cohort, and they show similar DNA methylation alteration.^673^ IDH mutants produce oncometabolite D-2-hydorxyglutarate (D2-HG) that competitively inhibits α-KG-dependent TET enzymes. In addition to disturbed DNA demethylation, JMJC domain demethylase-mediated histone demethylation and FTO-mediated RNA demethylation are also impaired.^674–676^ Therefore, it is not surprising that treatment of IDH mutant tumors with DNA demethylating agents works modestly,^282,650,677^ and inhibitors targeting mutant IDH proteins are developed. Enasidenib and ivosidenib were approved by FDA in 2017 and 2018 for adult patients with relapsed or refractory (R/R) AML with IDH2 and IDH1 mutations, respectively. The clinical trials showed the overall response rate is 41.6% for ivosidenib in IDH1-mutated patients with R/R AML (NCT02074839) and 38.8% for enasidenib in IDH2-mutated patients (NCT01915498).^678,679^ Clearance of IDH mutant clones is correlated with the complete remission.^678,679^ The frequency of *IDH1* mutations is high in patients with glioma (~70% of lower-grade gliomas), therefore, the development of inhibitors capable to penetrate the blood-brain barrier is essential for therapy in these patients.^680^ Vorasidenib is the first-in-class, brain-penetrant dual inhibitor of mutant IDH1/2 for glioma therapy.^681^ In a first-in-human phase I trial (NCT02481154), vorasidenib is well tolerated, effectively reduces D2-HG level by about 93% in patients with recurrent or progressive IDH1-mutant glioma, and displays a preliminary antitumor effect in the subset of patients with non-enhancing glioma.^682,683^ DS-1001, another new brain-penetrant mutant IDH1 inhibitor, exhibits well distribution to the mouse brain and the ability to reduce D2-HG and suppress tumor growth in preclinical studies.^684^ The first-in-human phase I study of DS-1001 (NCT03030066) reported patients with recurrent or progressive IDH1-mutant gliomas respond to the treatment with well tolerance.^680^ A study of the inhibitor in patients with chemotherapy- and radiotherapy-naïve IDH1-mutated glioma is ongoing (NCT04458272).

**Table 1:** Selected clinical trials targeting DNA/RNA/protein methylation

Target	Inhibitor/agonist (name)	Condition	Phase	Year	Recruitment Status	Clinical trial number
**DNA methyltransferases**						
DNMT	Decitabine	AML with complex and/or monosomal karyotype	II	2017	Recruiting	NCT03080766
DNMT	VTD-101 ointment (decitabine ointment)	HPV-induced vulvar intraepithelial neoplasia (grade 2/3)	I	2023	Recruiting	NCT05717621
DNMT	Azacitidine + decitabine	Myeloid malignancies	I	2019	Recruiting	NCT04187703
DNMT	Guadecitabine (SGI-110)	AML	III	2016	Completed	NCT02920008
DNMT	5-aza-4′-thio-2′-deoxycytidine (NTX-301)	AML and MDS	I	2019	Recruiting	NCT04167917
DNMT + PD1	Decitabine + cedazuridine + paclitaxel + pembrolizumab	Metastatic triple-negative breast cancer	I	2023	Recruiting	NCT05673200
DNMT + BCL-2	Decitabine (or azacitadine) + venetoclax + cladribine + cytarabine	AML or high-grade MDS	II	2023	Not yet recruiting	NCT05766514
**TET2 (DNA demethylase)**						
TET2	Vitamin C	Intermediate or high risk MDS with TET2 mutations	I/II	2018	Recruiting	NCT03433781
TET2 + DNMT	Vitamin C + azacitidine	MDS and AML with TET2 mutations	II	2018	Completed	NCT03397173
**IDH mutations**						
IDH1	Ivosidenib (AG-120)	Advanced hematologic malignancies with an IDH1 mutation	I	2014	Recruiting	NCT02074839
IDH1	DS-1001b	IDH1-mutated gliomas	I	2017	Active, not recruiting	NCT03030066
IDH1	DS-1001b	Chemotherapy- and radiotherapy-naive IDH1-mutated WHO grade II glioma	II	2020	Active, not recruiting	NCT04458272
IDH1	Ivosidenib (AG-120)	Advanced cholangiocarcinoma with IDH1 mutations	III	2016	Completed	NCT02989857
IDH2	Enasidenib (AG-221)	Advanced hematologic malignancies with an IDH2 mutation	I/II	2013	Active, not recruiting	NCT01915498
IDH1/2	Vorasidenib (AG-881)	Advanced solid tumors, including gliomas, with an IDH1 and/or IDH2 mutation	I	2015	Active, not recruiting	NCT02481154
IDH1/2	Vorasidenib (AG-881)	Residual or recurrent grade 2 glioma with an IDH1 or IDH2 mutation	III	2019	Active, not recruiting	NCT04164901
IDH1/2 + FLT3	Gilteritinib + ivosidenib or enasidenib	R/R AML with FLT3/IDH1 or FLT3/IDH2 mutations	I	2023	Not yet recruiting	NCT05756777
**PRC2 (H3K27 methyltransferases)**						
EZH2	Tazemetostat	Advanced solid tumors and B-cell Lymphomas	I/II	2013	Completed	NCT01897571
EZH2	Tazemetostat	INI1-negative tumors or R/R synovial sarcoma	II	2015	Active, not recruiting	NCT02601950
EZH1/2	HH2853	R/R non-Hodgkin’s lymphomas and advanced solid tumors	I/II	2020	Recruiting	NCT04390737
EZH1/2	HM97662	Advanced or metastatic solid tumors	I	2022	Recruiting	NCT05598151
EZH1/2	Valemetostat tosylate	R/R adult T-cell leukemia/lymphoma	II	2019	Active, not recruiting	NCT04102150
EZH2 + PD1	Tazemetostat + pembrolizumab	Advanced non-small cell lung cancer	I/II	2022	Not yet recruiting	NCT05467748
EZH2 + HDAC	Tazemetostat + belinostat	R/R lymphomas	I	2022	Recruiting	NCT05627245
EZH2 + CD20	Tazemetostat + rituximab +bendamustine	Follicular lymphoma	I	2022	Recruiting	NCT05551936
EZH2 + CDK4/6	Tazemetostat + palbociclib + CPX-351	R/R AML	I	2022	Not yet recruiting	NCT05627232
**DOT1L (H3K79 methyltransferase)**						
DOT1L	Pinometostat	Pediatric patients with R/R MLL-r leukemias	I	2014	Completed	NCT02141828
DOT1L	Pinometostat	R/R leukemias involving MLL-r or advanced hematologic malignancies	I	2012	Completed	NCT01684150
DOT1L + DNMT	Pinometostat + azacitidine	R/R or newly diagnosed MLL-r AML	I/II	2018	Completed	NCT03701295
DOT1L	Pinometostat + standard chemotherapy	Newly diagnosed MLL-r AML	I/II	2018	Terminated	NCT03724084
**PRMT5 (arginine methyltransferase)**						
PRMT5	GSK3326595	Early stage breast cancer	II	2020	Completed	NCT04676516
PRMT5	JNJ-64619178	Advanced solid tumors, non-Hodgkin Lymphoma, and MDS	I	2018	Active, not recruiting	NCT03573310
PRMT5	TNG462	MTAP-deleted solid tumors	I/II	2023	Not yet recruiting	NCT05732831
PRMT5	TNG908	MTAP-deleted solid tumors	I/II	2022	Recruiting	NCT05275478
**LSD1 (H3K4/H3K9 demethylase)**						
LSD1	TCP + ATRA	AML and MDS	I	2014	Completed	NCT02273102
LSD1	SP2577 + cyclophosphamide + topotecan	R/R ewing or ewing-related sarcomas	I	2018	Recruiting	NCT03600649
LSD1	SP2577	Advanced solid tumors	I	2019	Completed	NCT03895684
LSD1	SP2577	Sarcomas	I/II	2022	Enrolling by invitation	NCT05266196
LSD1 + DNMT	SP2577 + azacitidine	MDS or chronic myelomonocytic leukemia	I/II	2021	Active, not recruiting	NCT04734990
LSD1/HDAC6	JBI-802	Advanced solid tumors	I/II	2022	Recruiting	NCT05268666
LSD1	CC-90011 + rifampicin + itraconazole	R/R solid tumors and non-Hodgkin’s lymphomas	I	2016	Active, not recruiting	NCT02875223
LSD1	CC-90011 + cisplatin + etoposide	Small cell lung cancer	I	2019	Active, not recruiting	NCT03850067
LSD1 + BCL-2	Bomedemstat + venetoclax	R/R AML	I	2022	Recruiting	NCT05597306
LSD1 + PD-L1	Bomedemstat + atezolizumab	Small cell lung cancer	I/II	2022	Recruiting	NCT05191797
**KDM4C (H3K9 demethylase)**						
KDM4C	Caffeic Acid	ESCC	III	2017	Unknown	NCT03070262
KDM4C	Caffeic Acid	ESCC	III	2020	Unknown	NCT04648917
**METTL3 (mRNA methyltransferase)**						
METTL3	STC-15	Advanced malignancies	I	2022	Recruiting	NCT05584111
**Site-specific epigenome editing**						
MYC	OTX-2002	MYC-associated hepatocellular carcinoma and other solid tumors	I/II	2022	Recruiting	NCT05497453

Trial information is taken from ClinicalTrials.gov

### Targeting aberrant histone methylome

#### EZH2 and H3K27me3 methylome

EZH2 overexpression or gain-of-function mutation is associated with cancer progression and poor prognoses in many solid cancers and hematologic malignancies, such as prostate cancer, breast cancer, lymphoma, and AML.^685–687^ Inhibition of EZH2 or other PRC2 components diminishes the H3K27me3 level and derepresses the expression of tumor-suppressive genes, impairing cell proliferation and tumor growth in vivo. Multiple inhibitors are developed to target the SET domain of EZH2 to inhibit the catalytic activity, including EPZ-6438 (tazemetostat),^688^ CPI-1205,^689^ GSK126,^690^ C24,^691^ and UNC1999.^692^ Tazemetostat, a first-in-class oral EZH2 inhibitor, was approved by FDA in 2020 for the treatment of relapsed follicular lymphoma (Table 1). Supporting the approval, tazemetostat monotherapy exhibited meaningful and durable responses in patients with R/R follicular lymphoma in a phase II trial (NCT01897571).^693^ Specifically, the objective response rate is 69 and 35% and includes 13 and 4% complete response in the EZH2 gain-of-function mutant and wild-type cohorts, respectively.^693^ The antagonistic relationship between PRC2 and SWI/SNF chromatin remodeling complex provides a rationale for the use of EZH2 inhibitors in cancers with loss-of-function mutations in SWI/SNF complex members where unopposed EZH2 activity increases H3K27me3 level and causes excessive gene silence.^694^ Preclinical studies indicate inhibition of EZH2 is synthetic lethal with inactivation of SWI/SNF in a range of cancers with mutations in SWI/SNF subunits, such as *PBRM1*, *ARID1A*, *SMARCA2*, and *SMARCB1*.^695–698^ An international, open-label, phase II basket study (NCT02601950) in advanced epithelioid sarcoma with loss of *SMARCB1* showed tazemetostat is well tolerated with clinical activity (15% overall response);^699^ the results of this trial supported the FDA approval in 2020 for patients with metastatic or locally advanced epithelioid sarcoma not eligible for complete resection. Likewise, the pathognomonic SS18-SSX fusion protein in synovial sarcomas induces a state of SMARCB1-deficiency, conferring cancer cells with this chromosomal translocation sensitive to tazemetostat.^700^ Some H3K27me3-high malignancies are relatively tolerant to EZH2 inhibitors due to EZH1 compensation for EZH2 loss.^701^ This necessitates the development of EZH1/2 dual inhibitors, including valemetostat. Preclinical studies demonstrated that valemetostat is superior to an EZH2 selective inhibitor OR-S0 in terms of H3K27me3 depletion and antitumor efficacy.^702^ In a phase II trial of valemetostat in R/R adult T-cell leukemia/lymphoma (ATL) (NCT04102150), this inhibitor showed promising efficacy and tolerability, i*.*e., twenty-five patients have an overall response rate of 48%, including five complete remissions,^703^ which supports the drug approval in 2022 for treatment of aggressive ATL in Japan. Clinical trials of valemetostat in other cancer types (e.g., peripheral T-cell lymphoma, B-cell lymphoma, and tumors with SMARCB1 deficiency) and of two other dual inhibitors (HM97662 and HH2853) are underway^704^ (Table 1).

Despite these advances, the EZH2 enzymatic inhibitors cannot fully suppress the oncogenic function of EZH2, as non-catalytic or non-canonical activity of EZH2 also contributes to tumorigenesis.^705^ EZH2 acts as a coactivator for critical transcription factors to promote gene expression and oncogenesis, such as androgen receptor in prostate cancer,^706^ NF-κB in breast cancer,^707^ and MYC/p300 in *MLL1*-rearranged (*MLL-r*) leukemia.^708^ To repress the multifaceted activities of EZH2, an EZH2-targeting degrader MS177 was developed based on proteolysis-targeting chimera (PROTAC) technology.^708^ MS177 induces effective degradation of both canonical EZH2–PRC2 and noncanonical EZH2–MYC complexes, contributing to fast and more potent suppression of cancer growth compared with the enzymatic inhibitors.^708^

Careful monitoring of patients is required when targeting EZH2, as PRC2 can also function as a tumor suppressor. Loss-of-function mutations of EZH2 or other core PRC2 components (EED and SUZ12) are observed in multiple cancers, such as MDS,^709,710^ T cell acute lymphoblastic leukemia,^711,712^ malignant peripheral nerve sheath tumor (MPNST),^713^ and melanoma.^714^ Moreover, inactivating mutations appear to dominate all of the EZH2 mutations among 10,967 pan-cancer samples in The Cancer Genome Atlas.^715,716^ PRC2 inactivation leads to a global loss of H3K27me2/3 and aberrant transcriptional activation of carcinogenic signaling pathways (e.g., Ras signaling pathway) and developmentally silenced master regulators.^713,714^ Therapeutically targeting loss-of-function mutations of PRC2 members remains an unmet need. A recent RNAi screen targeting 565 known epigenetic/chromatin regulators identifies DNMT1 synthetic lethality with PRC2 inactivation in MPNST.^659^ Mechanistically, PRC2 inactivation enhances DNMT inhibition-mediated activation of retrotransposons and subsequent viral mimicry response.^659^ DNMT and EZH2 inhibitors synergize to amplify antitumor immune response in hepatocellular carcinoma with wild-type EZH2,^717^ consolidating DNMT1-targeted therapy can be used to treat PRC2-loss cancer. Additionally, PRC2 loss induces an epigenetic switch in *NF1* mutant cancers, sensitizing these cancers to BRD4-based therapies.^714^ Collectively, different strategies are developed to target cancers with EZH2 hyperactivity (inhibition of EZH2 enzymatic activity alone, EZH1/2 dual inhibition, or suppression of both canonical and non-canonical activity of EZH2) or with PRC2 loss (synthetic lethality with other targeted therapy), which provides an excellent paradigm to target a histone modifier with context-dependent functions and multiple acting mechanisms.

#### Inhibitors of other histone methyltransferases and demethylases

High-quality inhibitors have been developed for other histone methyltransferases, including DOT1L, PRMT5, G9a, GLP, SMYD2, SMYD3, SETD7, SUV420H1/2, and PRDM9.^651^ These inhibitors enable convenient evaluation of cancer cell dependency on the methyltransferases in different contexts and exploration of potential clinical use. The advancement of the DOT1L inhibitor into clinical trials provides a paradigm (Table 1). DOTIL drives the progression of multiple cancers, such as *MLL-r* leukemia,^718^ renal cell carcinoma,^719^ ovarian cancer,^720^ and breast cancer.^721^ DOT1L-mediated H3K79 methylation promotes transcriptional elongation and/or enhancer-promoter interaction,^722^ which is hijacked by oncogenic transcription factors, such as MLL-fusion proteins in *MLL-r* leukemia^718^ and estrogen receptor α in breast cancer,^721^ to activate downstream target genes. For this reason, DOT1L inhibitors were tested to treat *MLL-r* leukemia and antiestrogen-resistant breast cancer. Pinometostat (EPZ-5676), a first-in-class SAM competitive inhibitor, showed potent and selective inhibition with a subnanomolar affinity for DOT1L and encouraging curative effect in preclinical models.^723^ In a phase I trial in adult patients with advanced acute leukemias, particularly those bearing *MLL-r*, pinometostat diminishes H3K9me2 level and shows modest clinical activity, i.e., 2 of 51 patients bearing *MLL-r* experience complete remission.^724^ To further improve DOT1L inhibition-mediated therapy for *MLL-r* leukemia, the combination of pinometostat with existing standard-of-care drugs for acute leukemias, including DNMT inhibitors, was suggested according to synergistic anti-cancer activity observed in preclinical studies.^725^ Moreover, inhibition of DOT1L shows potent anti-cancer activity in a nude rat xenograft model of *Dnmt3a*-mutant AML.^726^ These results fueled two trials assessing pinometostat in combination with either standard-of-care chemotherapy (NCT03724084) or with azacytidine (NCT03701295) to treat R/R *MLL-r* leukemia. Currently, the former trial is terminated and the latter is completed, yet no result has been published.

Chemical interference of histone demethylases for cancer therapy also acquires great attention, with inhibitors of LSD1 and KDM4C advancing into clinical trials (Table 1). The FAD-dependent H3K4/H3K9 demethylase LSD1 is aberrantly expressed in multiple types of cancer, promoting cancer progression through regulating chromatin accessibility. Targeting LSD1 is becoming an emerging option for cancer therapy. There are two types of LSD1 inhibitors: irreversible and reversible inhibitors.^727^ Tranylcypromine (TCP), an inhibitor of monoamine oxidases (MAOs), has been identified as able to irreversibly repress LSD1, due to the sequence similarity between LSD1 and MAOs.^728^ Preclinical studies showed TCP treatment represses clonogenic potential and induces the differentiation of *MLL-r* leukemia stem cells and similarly the differentiation of all-*trans*-retinoic acid (ATRA)-insensitive AML cells through selective increase in H3K4me2 at related genes,^729,730^ which supports initiation of a phase I clinical trial to assess the safety and activity of ATRA plus TCP in patients with R/R AML and MDS(NCT02273102). Encouraging results that recapitulate preclinical studies were recorded, i.e., LSD1 inhibition sensitizes AML cells to ATRA, with an acceptable safety profile.^731^ The selective increase rather than a large-scale genome-wide increase of H3K4me2 may contribute to the low toxicity of LSD1 inhibition.^729,730^ Potential side effects of TCP as an LSD1 inhibitor, including drowsiness, dizziness, and orthostatic hypotension, are mostly attributed to the concomitant inhibition of MAOs.^732^ To improve selectivity and potency, multiple new inhibitors are developed using TCP as the lead compound, including ORY-1001, ORY-2001, GSK2879552, IMG-7289, and INCB059872. Clinical trials of these compounds for a range of cancers are ongoing, as well as other diseases including Alzheimer’s disease, multiple sclerosis, and myelofibrosis. Unlike irreversible inhibitors that covalent bind to the cofactor FAD of LSD1, reversible inhibitors act through competing for substrate or FAD-binding site or allosteric regulating LSD1 activity.^733^ Reversible inhibitors are clinically preferred due to potentially safer metabolism and lower toxicity. A large number of reversible LSD1 inhibitors are developed;^727^ two of them, CC-90011 (substrate competitive inhibitor)^734^ and SP2577 (allosteric inhibitor)^735^ have entered clinical trials for therapy of cancers including sarcomas, non-Hodgkin’s lymphomas, and small cell lung cancer (Table 1). Preliminary results of the phase I trial (NCT03600649) of SP-2577 in patients with R/R Ewing sarcoma exhibit a manageable safety profile with proof-of-concept preliminary antitumor activity, which supports the planned phase II expansion.^736^ KDM4C is an α-KG-dependent histone H3K9 demethylase that is amplified in various types of cancers, such as esophageal squamous cell carcinoma (ESCC), breast cancer, and medulloblastoma.^737,738^ Our lab and others demonstrated that KDM4C promotes proliferation and stemness of cancer cells by removing H3K9me3 from serine pathway genes (*PHGDH* and *PSAT1*) and stem cell-related genes (*NOTCH1*, *NANOG*, and *ALDH1A3*), respectively, which is disrupted by inhibition of KDM4C.^737,739–741^ Caffeic acid (CA), a micromolar KDM4C inhibitor,^742^ suppresses the demethylation activity in ESCC cells, accompanied by reduced cancer cell stemness,^739,743^ and phase III trials of targeting KDM4C with CA in ESCC patients are ongoing^744^ (NCT04648917 and NCT03070262).

### Targeting aberrant mRNA methylome

Targeting the mRNA methylation pathway presents a new direction for cancer therapy.^745^ METTL3 is the primary mRNA m^6^A methyltransferase, and the interest in the development of METTL3 inhibitors has been stimulated since the reported key oncogenic role of the gene in leukemia in 2017.^746,747^ STM2457 is a non-SAM analogous small molecule but competes for the SAM-binding site of METTL3 (IC_50_ = 16.9 nM), and is the first bioavailable METTL3 inhibitor that exhibits anti-cancer efficacy in a preclinical cancer model.^748^ Administration of tumors with STM2457 compromises AML growth and promotes differentiation and apoptosis, accompanied with selective depletion of m^6^A on known leukemogenic mRNAs and downregulation of their expression.^748^ Following, a derivative of STM2457, STC-15, was developed and showed activities in stimulating innate immune pathways, suppressing tumor growth, and augmenting the efficacy of anti-PD1 therapy with durable antitumor immunity in preclinical models of colorectal cancer and lymphoma.^749^ A phase I clinical trial of STC-15 in patients with advanced malignancies is ongoing (NCT05584111) (Table 1). Other SAM-competitive inhibitors of METTL3 include adenine derivatives,^750^ UZH1a,^751^ and UZH2.^752^ Among these, UZH2, a 1,4,9-triazaspiro[5.5]undecan-2-one derivative, shows the highest potency (IC_50_ = 5 nM) and favorable in vitro absorption–distribution–metabolism–excretion properties.^752^ Two allosteric inhibitors of METTL3, 43n (IC_50_ = 2.81 μM) and eltrombopag (IC_50_ = 3.65 μM), were recently discovered, and their effects on m^6^A level and cell proliferation were demonstrated in AML cells.^753,754^ Although current allosteric inhibitors have micromolar rather than nanomolar inhibitory efficacy of SAM competitive inhibitors, investment in the development of efficient allosteric inhibitors is warranted as most methyltransferases from DNA to proteins possess preserved SAM binding regions. Collectively, the development of these METTL3 inhibitors will greatly advance the functional study and therapeutic targeting of mRNA m^6^A methylation in cancer and other diseases.

### Site-specific correction of methylation for precise therapy

Although knowledge about the molecular and cellular mechanisms of DNA/RNA/protein methylation actions in various diseases increases explosively over the past decades, the translation of it into clinical practice is still challenging with only modest success in the cancer field. The methylation modifiers generally have genome/transcriptome/proteome-wide effects, regulating both tumor suppressor genes and oncogenes. Targeting these modifiers with current small-molecule compounds leads to global loss or gain of cognate methylation marks and global changes in gene expression, as well as in nonhistone protein function. This will inevitably introduce undesirable biological effects and cause cytotoxicity, restricting the use of these compounds. For instance, in hematologic malignancies where hypomethylating therapy is relatively more successful, demethylation and upregulation of oncogene occur in patients after DNMT inhibitor treatment.^654^ The risk would be much greater when administration of these compounds aims to reactivate a specific gene for the treatment of monogenic disorders, such as *FRM1* for FXS and *MeCP2* for RTT.^189,755,756^ In addition to potential off-target toxicity, monotherapy with DNA hypomethylating agents showed limited efficacy in terms of reactivation of these stable silenced target genes.^755,757^

**Fig. 8:**
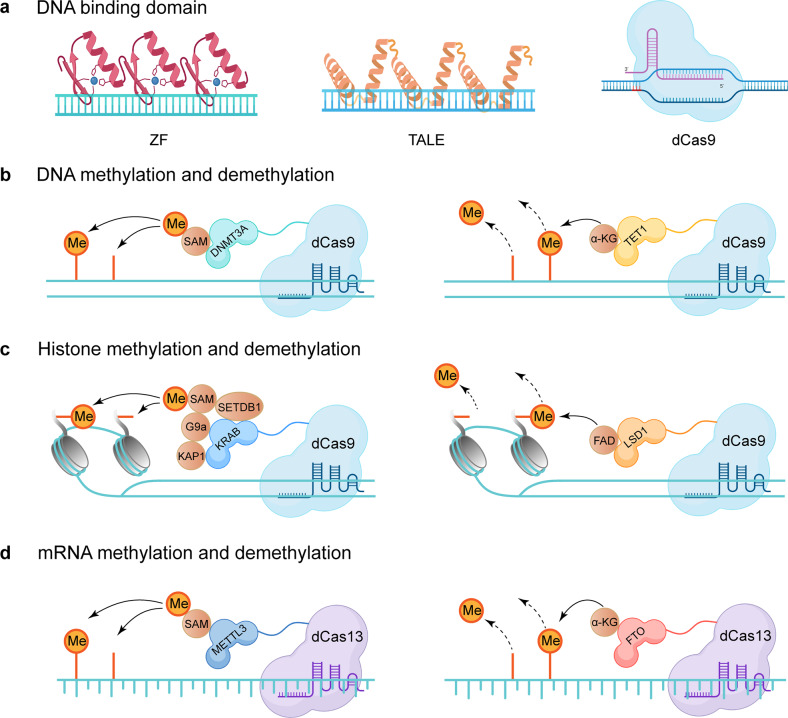
Principle of site-specific manipulating DNA, histone, and RNA methylation. **a** Three generations of programmable DNA-binding domain (DBD) platforms for site-specific chromatin methylation manipulation: zinc fingers (ZFs), transcription activator-like effectors (TALEs), and catalytically dead CRISPR-dCas system. **b** Fusion of dCas9 to DNA methyltransferases (e.g., DNMT3A) or demethylases (e.g., TET1) allows for targeted DNA methylation or demethylation, respectively. **c** Fusion of dCas9 to protein methylation machinery (e.g., KRAB) or demethylases (e.g., LSD1) enables artificial histone methylation or demethylation at defined loci, respectively. **d** Fusion of dCas13 to RNA methyltransferases (e.g., METTL3) or demethylases (e.g., FTO) allows for selective deposition or removal of m^6^A signals at specific transcripts, respectively

The development of tools (hereafter designated as methylation editors) capable of site-specific manipulating DNA and histone methylation status holds great promise to address the above challenges. Rather than methylome-wide alteration induced by directly targeting methylation machinery, chromatin methylation editors use sequence-specific DNA-binding domains (DBDs) to place the methylation machineries at the defined loci to perform DNA/histone methylation or demethylation, which in turn modulates the transcription of the targeted genes. Three generations of programmable DBD platforms are developed based on zinc fingers (ZFs), transcription activator-like effectors, and catalytically dead CRISPR-dCas system, with the CRISPR-based editors getting the most attention due to their easy reprogramming^758,759^ (Fig. 8a). Chromatin methylation editors are now available for the commonly studied methylation substrates, including CpG (by DNMT3A or TET1), H3K4 (by PRDM9, SMYD3, and LSD1), H3K9 (by KRAB, G9a, and HP1), H3K27 (by EZH2 and FOG1), and H3K79 (by DOT1L)^760^ (Fig. 8b, c).

**Fig. 9:**
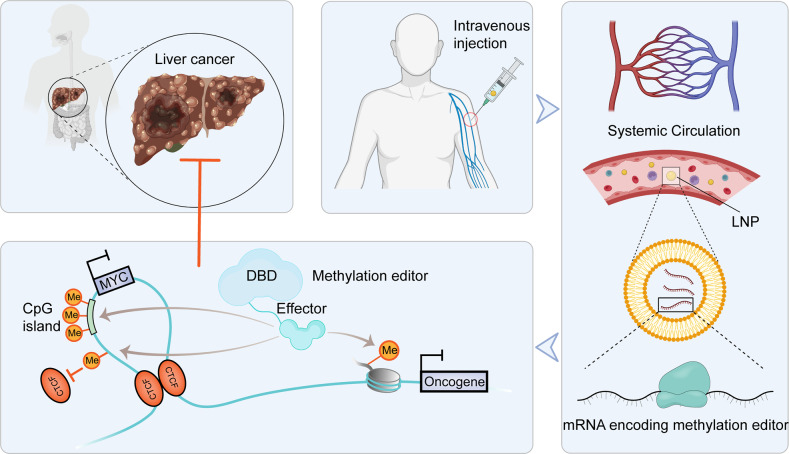
Paradigm of site-specific manipulating chromatin methylation for precise cancer therapy. mRNAs encoding a methylation editor are delivered to liver cancer cells by lipid nanoparticles (LNPs), which suppresses oncogene expression by catalyzing DNA and/or histone methylation at the promoters of oncogenes (e.g., MYC) or at the CTCF-binding sites to abolish their interactions with potential enhancers

The application of these tools for methylation and disease correction generates encouraging preclinical results. dCas9-TET1-mediated demethylation of the mutant *FMR1* gene reactivates the gene expression and rescues behavioral defects in the FXS patient-derived cells.^191^ By coupling dCas9-TET1-mediated demethylation with dCpf1-CTCF-mediated insulation, the healthy copy of *MECP2* on the inactive X chromosome is effectively activated in RTT human embryonic stem cells and derived neurons, which rescues RTT-related neuronal abnormality.^757^ In cancers, dCas9-TET1 has been adopted to unleash silenced tumor suppressor genes, e.g., *BRCA1* (in breast and cervical cancer),^761^ *SARI* (in colon cancer),^762^ and *NNT* (in lung cancer),^763^ to combat tumor progression and chemoresistance. On the other hand, dCas9-based repressors that contain DNA methyltransferases (e.g., DNMT3A and DNMT3L) and repressive histone methyltransferases (e.g., KRAB and EZH2) alone or in combination have been used to silence oncogenes in cancers, such as *GRN* in liver cancer,^764^ *BRAF* and *HER2* in colon cancer,^765,766^ *FGFR4* in breast cancer,^767^ and *KRAS* in pancreatic cancer.^765^ Targeted methylation of the promoter of amyloid precursor protein gene *APP* by dCas9-DNMT3A rescues neuron cell pathology in vitro and in vivo of mouse AD model.^768^ Alternative to directly target promoters or transcription start sites in the above cases, methylation editing of CTCF-binding sites can alter 3D genome structure, affect the interaction between the distal enhancer and promoters, and in turn, control gene transcription of targeted genes.^769,770^ Multiple oncogenes including *MYC*, *TERT*, and *CCND1*, are influenced by recurrent changes of the 3D chromosome architecture in diverse cancer types.^771–773^ Artificial methylation of a CTCF-binding site located 2 kb upstream of the *MYC* promoter by dCas9-DNMT prevents the docking of cancer-specific super-enhancers, which compromises *MYC* expression and cancer cell proliferation.^774^ Recently, an epigenomic controller (called OTX-2002) developed by Omega Therapeutics to target the CTCF-insulated loop domain-containing *MYC* has advanced to a clinical trial for *MYC*-associated hepatocellular carcinoma and other solid tumors (NCT05497453)^775^ (Fig. 9 and Table 1). These chromatin methylation editors can also be used to treat various diseases without methylation-based etiology, through upregulating or downregulating the expression of protective or pathogenic factors, respectively. For instance, ZFN-KRAB repressors that recognize the amplified CAG repeats within the mutant *HTT* are designed to selectively silence the mutant allele for the treatment of Huntington’s disease, and a one-time striatal AAV-mediated delivery can correct molecular, histopathological, and behavioral defects in the mouse model.^776^ Besides therapeutic efforts, methylation editors are helping dissect the causal relationship between methylation alteration and tumorigenesis, which will identify faithful cancer driver methylation changes that serve as potential therapeutic or diagnostic targets.^280,777^

With the help of PAMmer oligonucleotides, dCas9 can target mRNAs, and the first generation of site-specific m^6^A editors was developed in 2019 by fusing dCas9 with the methyltransferases (METTL3/METTL14) or demethylases (ALKBH5 or FTO).^778^ Subsequently, the m^6^A editors are optimized by substituting dCas9 with smaller Cas13 proteins (Fig. 8d), with lower off-target activity and PAMmer oligonucleotide-independence.^779–782^ Furthermore, chemically or light-inducible versions of m^6^A editors are developed to manipulate mRNA methylation spatiotemporally.^783,784^ Editing individual transcripts in cancer cells inhibits proliferation, migration, or therapy resistance, such as *EGFR* and *MYC* in cervical cancer,^780^ *FOXM1* and *MYC* in glioblastoma,^779^ *FGFR4* in HER2-positive breast cancer,^767^ *ZNF677* and *BTG2* in renal cell carcinoma (RCC),^785,786^ as well as lncRNA *NEAT1* in RCC.^787^ Temporal removal of m^6^A from the *SOX2* transcripts is sufficient to modulate the differentiation of human pluripotent stem cells.^788^ Therefore, the implementation of RNA methylation editors represents a potentially new and effective way to specifically alter the expression of targeted genes for cell fate and disease control.

## Conclusion and outlook

Reversible tagging methyl groups on DNA, RNAs, histones, and nonhistone proteins finetunes gene expression and function. Dysregulation of the process caused by genetic mutations or environmental stimuli promotes various diseases and accelerates aging. This general outline is right but not nearly enough. We need to delve deeply into the details and deconvolute the complex networks to find faithful disease-driver methylation targets,^280^ which is a formidable task. Advances in the technologies, including CRISPR-assisted library screening,^777,789^ spatial transcriptomics,^790,791^ and DNA/RNA/protein methylation assays,^792–794^ will undoubtedly help, however, the field has its intractable problems. The function of methylation pathways is highly context-dependent, making annotation of their role in diseases complex and difficult. Limitation in animal models, such as failure to recapitulate intratumor heterogeneity seen in humans in genetically engineered mouse models, lack of effective immune system in patient-derived xenograft models, and/or shortage of models to study cancer and aging simultaneously,^795^ is a commonplace problem when translating the preclinical finding into the clinic, which is likely be more severe in the methylation field due to the plastic roles of methylation pathways. Consistently, predictive biomarkers for patient selection remain elusive when targeting methylation.

Another intractable problem is the global changes in methylomes caused by current inhibition strategies (e.g., chemical or genetic interference of methylation modifiers), which confounds the interpretation of results and compromises the therapeutic effects. The root cause of even monogenic diseases (e.g., FXS and RTT), let alone multigenic diseases involving different directions of methylation changes (e.g., cancer and aging-related diseases), can hardly be effectively and safely tackled by simple interference with the modifiers. While monotherapy targeting methylomes has not generated the expected clinical outcomes, combining the epigenetic drugs with other therapies, including chemotherapy, radiation therapy, molecular targeted therapy, and immunotherapy, shows encouraging anti-cancer synergistic effects,^796^ which is associated with the important role of methylation remodeling in the regulating tumor immunogenicity, DNA repair, therapy resistance, among others. A number of clinical trials are ongoing to test different combination strategies (Table 1), and whether the combination therapy represents the direction of the future yet has no conclusion. However, given the context-dependent role of methylation pathways, lack of predictive biomarkers, and unidirectional methylome perturbance, rational design of combination drug regimens for individual cancer patients remains challenging.

The development of precise methylation editing technologies raises boundless opportunities to meet the above challenges. One key opportunity for the future will be cross-disciplinary cooperation to find appropriate methods to perform in vivo methylation editing for therapy. The expression changes of some genes, especially those involving gain of DNA and repressive histone methylation, can be long-lasting and heritable, which is clinically preferred as a single dose of administration can induce durable therapeutic effects.^200,797,798^ Using mRNA-lipid nanoparticle (mRNA-LNP)-based therapeutics that has been adopted to combat the COVID-19 pandemic during past years and liver-accumulation feature of LNPs, an epigenome editor (OTX-2002) targeting *MYC* for the treatment of liver cancer recently received clearance of Investigational New Drug by US FDA to start a phase I/II clinical trial (Fig. 9 and Table 1). This marks an exciting beginning, however, to realize the therapeutic promise of methylation editors, several hurdles related to science and technology need to be addressed. First, many genes show modest and transient gene expression alteration using the current tools. Therefore, the efficacy of methylation editors needs to be improved and the contextual cues for persistent changes to gene regulation require to be clarified.^799–801^ Second, the efficacy, specificity, and safety of in vivo delivery remain to be a bottleneck in the treatment of diseases, and specific requirements for delivery vehicles vary with diseases.^802–805^ Third, immune response to methylation editors in humans.^806^

Another key opportunity is that the causal relationship between the methylation alteration of specific sites or genes with disease phenotypes and its underlying mechanism could be finally answered, which cannot be achieved by current gene-deletion/-overexpression mimic models. The future diagnosis and selection of the most promising therapies for individual patients will rely on this knowledge. Particularly, DNA methylation-based non-invasive liquid biopsy (e.g., plasma cell-free DNA) tests hold great promise in cancer early detection, prognosis, and therapy monitoring, which would transform cancer survival.^807–811^ The sensitivity of individual gene-based methylation models or the generalization ability of genome-wide methylation models needs to be further improved for clinical implementation as a standard of care.^812^ Finding and validating the most informative DNA methylation biomarkers is crucial for future success when they are used alone or combined with other cancer markers (e.g., cell-free DNA mutations).^813,814^ In addition, like we can’t merely focus on DNA methylation and ignore the important role of RNA and protein methylation in the progression of mIDH-driven cancers, RNA and protein methylation may serve as orthogonal predictors, and synchronous detection of methylation across the central dogma might improve diagnostic power (especially in the diseases with disordered metabolism) based on tissue biopsies and even liquid biopsies.^815^
